# Filling the gap: brief neuropsychological assessment protocol for glioma patients undergoing awake surgeries

**DOI:** 10.3389/fpsyg.2024.1417947

**Published:** 2024-08-09

**Authors:** Juliana Bastos Ohy, Cleiton Formentin, Daniel Andrade Gripp, Joab Alves Nicácio Jr, Maíra Cristina Velho, Larissa Núbia Vilany, Gabriel Frizon Greggianin, Beatriz Sartori, Ana Carolina Pinheiro Campos, Silvia Mazzali Verst, Marcos Vinicius Calfat Maldaun

**Affiliations:** Department of Neuro-Oncology, Sírio Libanês Hospital, Sao Paulo, Brazil

**Keywords:** neuropsychological assessment, awake craniotomy, glioma, language mapping, maximal safe resection

## Abstract

**Introduction:**

The literature lacks a concise neurocognitive test for assessing primary cognitive domains in neuro-oncological patients. This study aims to describe and assess the feasibility of the Ohy-Maldaun Fast Track Cognitive Test (OMFTCT), used to pre- and post-operatively evaluate patients undergoing brain tumor surgery in language eloquent areas. The cognitive diagnosis was used to safely guide intraoperative language assessment.

**Methods:**

This is a prospective longitudinal observational clinical study conducted on a cohort of 50 glioma patients eligible for awake craniotomies. The proposed protocol assesses multiple cognitive domains, including language, short-term verbal and visual memories, working memory, praxis, executive functions, and calculation ability. The protocol comprises 10 different subtests, with a maximum score of 50 points, and was applied at three time points: preoperative, immediately postoperative period, and 30 days after surgery.

**Results:**

Among the initial 50 patients enrolled, 36 underwent assessment at all three designated time points. The mean age of the patients was 45.3 years, and they presented an average of 15 years of education. The predominant tumor types included Glioblastoma, IDH-wt (44.1%), and diffuse astrocytoma, IDH-mutant (41.2%). The tumors were located in the left temporal lobe (27.8%), followed by the left frontal lobe (25%). The full test had an average application time of 23 min.

**Conclusion:**

OMFTCT provided pre- and postoperative assessments of different cognitive domains, enabling more accurate planning of intraoperative language testing. Additionally, recognition of post-operative cognitive impairments played a crucial role in optimizing patient care.

## Introduction

Diffuse gliomas are the most common primary malignant brain tumors in adults (Ostrom et al., 2022). Recent studies have provided strong evidence that the extent of tumor resection, as well as postsurgical tumor volume, are associated with further progression and overall survival in diffuse glioma patients (Brown et al., 2016; Wijnenga et al., 2018; Molinaro et al., 2020). Currently, the concept of resection has changed from arbitrary categories to a new model of maximal and safely achievable volumetric resection, in a delicate balance between achieving maximal tumor resection and inducing new deficits (Duffau, 2023).

Since the original description of awake craniotomy by Horsley, this technique has been used for different indications with the overarching goal of enhancing safety (Horsley, 1887). Intraoperative mapping is essential to prevent postoperative neurological deficits, and has been associated with improved neurological, functional, cognitive, radiological and survival outcomes in both low-grade and high-grade glioma patients (Sanai et al., 2008; De Witt Hamer et al., 2012; Hervey-Jumper et al., 2015; Bu et al., 2021; Motomura et al., 2021; Fukui et al., 2022; Gerritsen et al., 2022).

Suitable patient selection and perioperative neuropsychological assessment are essential to ensure intra- and postoperative success with awake mapping (Rofes et al., 2017; Ruis, 2018). In this context, preoperative neuropsychological evaluation is of paramount importance for identifying subtle deficits, since around 50% of diffuse glioma patients present neurocognitive impairments before surgery, which may have gone unnoticed due to slow tumor growth and neuroplasticity. Which have been linked to negative impacts on both quality of life and survival rate (Aaronson et al., 2011; Schei et al., 2022; Sierpowska et al., 2022; Gasa-Roqué et al., 2024). Preoperative neuroimaging reveals various forms of functional redistribution, such as persistent function within the tumor or recruitment of contralateral hemisphere areas (Duffau, 2014). Traditional language tests like the Aachen Aphasia Test or Boston Naming Test may not fully capture these deficits (Satoer et al., 2014). However, there are few concise and comprehensive neurocognitive tests published in the literature for evaluating primary cognitive domains affected in neuro-oncological patients, including an absence of this type of test in Portuguese language.

After gaining expertise with the creation and validation of the intraoperative test Verst-Maldaun Language Assessment (VMLA; Verst et al., 2020), we decided to address the gap of a concise and comprehensive preoperative test for native Portuguese speakers, developing the Ohy-Maldaun Fast Track Cognitive Test (OMFTCT). OMFTCT is the second step of our ongoing Language Tracking Project and focuses on assessing production and comprehension at main linguistic domains such as phonology, semantics, and morphosyntax. As a low-income country, there is either lack of specialized neuropsychologists in neuro-oncology or the few available are centered in main cities. Normally, the currently used perioperative cognitive tests demand many hours for complete application, require licenses and are expensive for most of the Brazilian population. The development of OMFTCT required the expertise of the team involved in the creation of the VMLA (MVCM and SMV), who was involved in tasks selection and application dynamics. OMFTCT used VMLA images database, which is available elsewhere (Verst et al., 2020). The main researcher (JBO) had already experience with on-site and online neuropsychological cognitive rehabilitation for elderly.

This study aims to simplify neurocognitive evaluation in the context of perioperative assessment for patients undergoing awake craniotomies, focusing on language production and comprehension at key linguistic levels such as phonology, semantics, and morphosyntax.

## Materials and methods

This is a prospective longitudinal observational clinical study conducted on a cohort of 50 patients eligible for awake craniotomies for brain tumors, who were followed by a specialized neuro-oncology team at *Sírio-Libanês Hospital* in Sao Paulo, Brazil, from September 2019 to June 2022.

Sample eligibility was based on the following inclusion criteria: adults aged 18 years or older, underwent awake surgery, with a highly suspicious neuroimaging of unifocal glioma in the dominant hemisphere, as determined by functional MRI. The exclusion criteria included: prior cognitive disturbance, temporospatial disorientation, prolonged hospitalization in the preoperative period (more than 15 days), seizures within the last 24 h before surgery, and failure to complete the assessments and follow-up of the study.

### OMFTCT protocol conception

OMFTCT domains and tasks were chosen based on Wechsler Intelligence Scales, Raven’s Progressive Matrices, Rey’s Complex Figures, Benton’s Visual Retention Test and the Token Test (Costa et al., 2018). A systematic review identified the most used tests in patients with glioma, aiming to detect impairments in memory, attention, and executive functions. Therefore, OMFTCT was designed to match them, and the WAIS-III scales (do Nascimento, 1993; Lopes et al., 2012). WAIS-III scales are a set of 14 subtests normally applied to groups aging from 16 to 89 years. Their average application time is 90 min. WAIS-III is divided into three scales: verbal, execution and total, and four factor indices: verbal comprehension, perceptual organization, operational memory and processing speed. OMFTCT encompasses 10 subtests, focusing on language, memory, attention, executive functions and praxis.

The OMFTCT cognitive protocol was methodologically developed in the following steps: (1) identification of demands related to cognitive functions during the evaluation process of patients undergoing awake surgery; (2) review of the literature addressing the instruments used in the context of cognitive assessment for patients undergoing awake craniotomies; (3) multidisciplinary team discussion and conception of the protocol, which included selection of cognitive domains to be assessed and set up of the tests; (4) selection of images according to a validated protocol tailored to Brazilian native speakers. Images had been purchased from Shutterstock.com to compose the database of Verst-Maldaun Language Assessment by one of the authors (SMV). The receipts numbers are SSTK-0CA8F-1358 and SSTK-0235F-6FC2 (Verst et al., 2020); (5) analysis of the applicability of the protocol, taking into consideration patients’ cooperation; and (6) establishment of the dynamics of the cognitive protocol, including the duration of the test application.

### OMFTCT subtests

OMFTCT assesses multiple cognitive domains, including language, short-term verbal and visual memories, working memory, praxis, executive functions, and calculation ability (Table 1). The protocol comprises 10 different subtests, scoring zero to five for any subtest. The maximum total score is 50 points (Supplementary material – full version of the test). Each subtest comprises 5 items that are presented separately and require immediate answer after each item. Each item is presented separately, and the patient should answer immediately after each one.

1. Naming: Five colored images from both living and nonliving categories, including animals, objects, and food, are presented. The first three images should be named individually, while the last two should be named in combination with a dual task (alternately opening and closing the hands), to address any deficiencies in working memory. The tester asked, “what is this?” and the patient should answer “this is a/an (object).” Correction and scoring: the patient must respond within 20 s. If the patient provides a correct answer after the allotted time, it should be noted as qualitative data, but not scored.
2. Verbal memory: The tester presented separately two sequences of phrases, one sequence of words, one sequence of letters, and one sequence of numbers are dictated, and the patient must repeat them after each sequence. Correction and scoring: To receive a score for this subtest, the dictated sequence must be repeated without any alterations to its elements. Items examples:
  - Gray is the color of the mouse that got into the yellow house
  - The gray mouse entered the yellow house which had a red door in the laundry room
  - Shoe–swallow–plum–glove–branch
  - 6–0–9–0–3–5–2
  - Q–J–D–I–V–O–P–W–L
3. Semantics: In this subtest, both verbal and nonverbal semantics are employed. The patient is required to identify the correct combination of three images (based on VMLA-Semantics), or identify the object’s utility, or identify the material it is made of, or identify the shape of a specific figure. Correction and scoring: the patient should name or point to the proper combination figure give its utility, material or shape. If the patient is unable to provide the name (for the first three items), but points to the figure, the answers should be scored as correct, and the difficulty of naming should be recorded as qualitative data. Items examples:

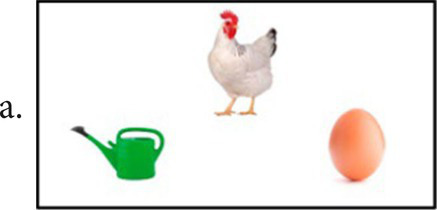

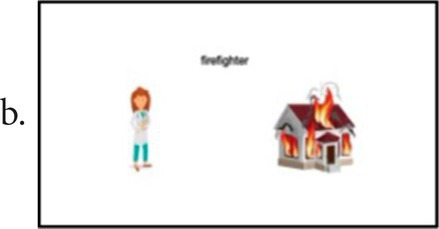

c. “What is it for?” On the tablet screen is the word: NAIL
d. “What material is it made of?” On the tablet screen is the picture of: GLOVES
e. “What is the object’s shape? On the tablet screen is the picture of: PEN

**Table 1:** OMFTCT subtests and evaluated cognitive domains.

Subtest	Main cognitive domain	Associated cognitive domain
Object naming (with dual task)	Language	Language, visual perception, attention, context memory
Verbal memory	Short-term verbal memory	Verbal memory, attention, auditory perception
Semantics (with dual task)	Language	Language, semantic memory
Calculation	Logical-mathematical reasoning	Logical-mathematical reasoning attention
Writing	Praxia, linguagem	Praxia, Language attention, hand-eye coordination
Visual memory	Visual memory	Visual memory, attention, object naming, visual perception
Reading	Language	Language, visual perception, attention, logical reasoning.
Word formation	Language	Language, executive function
Drawing	Praxia	Praxia, attention, visuospatial organization, hand-eye coordination, executive function
Spelling	Working memory	Language, working memory, attention

For items c, d, e the patients is asked to touch his left ear with his right hand while answering

1. Calculation: Patients are verbally presented with three mathematical operations and two abstract-logical reasoning problems, and they are required to mentally solve them. Calculations must be performed mentally, and the use of paper, pen, or a calculator is not allowed. Items examples:

- 5 + 6=
- 12–7=
- 25×6=
- Which of the following sequences of numbers add up to 13? On the tablet screen is the picture of: 5 8 3 10 2 9
- “How many quarter coins are needed to get 4 dollars?” Correct answer: 16

1. Writing: This subtest is divided into two separate stages: the first stage involves the patient copying two words and one sentence, which are presented in the tablet screen. After, a word and a sentence are separately dictated, and the patients should write them on an answer sheet. Correction and scoring: the patient must write the words and sentences correctly. Errors such as changing, omitting, or adding letters are not scored. Incorrect accentuation and punctuation are not considered errors. Items examples:

- ANALOGY
- CAREER
- CARRIED A TON OF ORANGES IN A LARGE BAG
- Dictation: RAILROAD
- Dictation: THERE WAS NO ALTERNATIVE BUT TO FIND HIM

1. Visual memory: A picture A is presented to the patient to memorize for 15 s. After memorization, a picture B, which contains the same images as picture A and additional distracting images, is presented. The patient is then required to identify and verbalize/ point which images were originally presented in Picture A. There is progressively addition of objects to be memorized in subsequent tasks. Initially, there are 2 items, to be memorized among 4 shown (2/4). Subsequently, a ratio of presented to total (presented and distractors) objects is 4/ 8, 6/9, 8/12, 10/18. Correction and scoring: The patient must correctly identify and name all the images that appeared in Picture A. Item example:

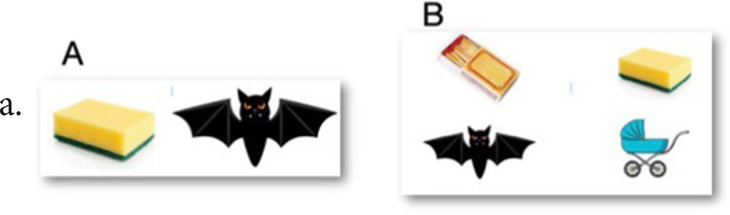

1. Reading: The patient is instructed to read a set of 5 sentences, commonly referred to as “tongue twisters,” to evaluate their reading ability and logical reasoning. Correction and scoring: the patient is expected to read all the items correctly. Example: *She sells seashells by the seashore* (English); “*Trazei três pratos de trigo para três tigres tristes comerem”* (Portuguese).
2. Word formation: The patient is presented with items related to word formation, grammar, and vocabulary. Correction and scoring: the patient is required to provide exact answers to all the items. Items examples:

- Form the word: VI–TE–SI–ON–LE.
- Form the word: PLA-NE-AIR
- Form the word: LA-CHO-TE-CO
- Syntax: The word LOYAL, can be added to which prefix? A) UN. B). DIS. C). MIS.
- “Where can we buy books?

1. Drawing: The patient is instructed to spontaneously draw three geometric shapes that are described verbally and to copy two other shapes that are presented graphically. Correction and scoring: the patient must draw all items with the correct number of sides and angles to receive a score. Items examples:

a. Draw a circle
b. Draw a triangle
c. Draw a cube
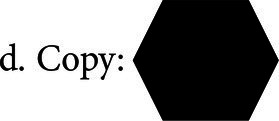
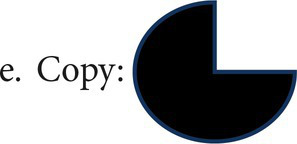

1. Spelling: The patient is required to spell five words, with the first three in regular order and the last two in reverse order. Correction and scoring: the patient must provide the exact answer for all items without writing the words on paper to receive a score.

- “Spell in direct order”: CHAIR
- “Spell in direct order”: SHARK
- “Spell in direct order”: SCREEN
- “Spell in inverted order”: HAMMER
- “Spell in inverted order”: INCOME

### Data collection

Patient data were collected by an OMFTCT-trained tester, who could be one of the following team members: the neurosurgeons, neurosurgery residents, neurosurgical oncology fellows, or neuropsychologists. The neuropsychologist (J.B.O.) explained the protocol application, provided a 10-min video tutorial on test administration and was available for clarifying any doubt. OMFTCT is available at vemotests.com. Patient data is filled in the platform, including age, gender, educational level, location of the lesion. Patients’ data were also collected in a paper form, with additional information on occupation, cognitive, emotional, and clinical symptoms, tumor characteristics and date of diagnosis.

The tester explained the test to the patient and sat in front of him so that the tablet could be directly in front of patient’s eyes. Patient’s glasses were used whenever needed for proper execution of the tasks. Dual task was included as part of subtests 1 and 3 either to check on praxis, attention or working memory and was further included in the report as a qualitative data. For all subtests a screen was presented to the patient, except or subtest 10. Scores of subtests 5 and 8 were written down into a blank sheet. All the subtests’ scores were analyzed and discussed with the main researcher, who was responsible for the final report.

The OMFTCT protocol was applied at three different time points: preoperatively (up to 1 week before surgery, T1), immediately after surgery (up to 48 h after surgery, T2), and late after surgery (30 days after surgery, T3). Pre-. immediate, and late postoperative tests were performed by the same person. A slightly different version of the protocol, containing the same subtests, was applied, aiming to avoid patient’s learning and bias.

T1 was conducted either in-person at a clinic or following hospital admission. If necessary, remote on-line assessment was carried out, particularly at T3, for patients living in different states of the country. Then, the Zoom app was used, allowing screen sharing for specific tasks. Subtests 5 and 9 were performed on a paper sheet, which was photographed and sent for the tester for scoring evaluation and further assessment. During the study, the neuropsychologist was responsible for qualitative evaluation of all applied tests and for writing the test report.

This study was performed according to the principles of the Declaration of Helsinki, and approved by the Research Ethics Committee of the *Sírio-Libanês Hospital* (number 1134). All patients signed the Informed Consent Form.

### Statistical analysis

The descriptive analysis of the collected data included variables such as age, sex, education in years, tumor type and location, histological grade, and preoperative cognitive, emotional, and clinical symptoms. We performed comparisons between the three assessment times using the Friedman test, and after dichotomization (with a score of 40 or more considered a good result), we used Cochran’s Q test.

## Results

### Sample characteristics

From the 50 patients initially recruited, 36 completed the evaluation at all three distinct time points. However, 14 patients skipped the T3 assessment due to either withdrawal or because they were beginning adjuvant treatment and could not find the time to undergo the testing. Table 2 presents demographic and preoperative data for the patients who completed the research protocol (n = 36). The mean age of the patients was 45.3 years, and educational level translated an average of 15 years of education. There was an equal distribution of 50% male and 50% female patients. Most tumors were glioblastoma, IDH-*wt* (44.1%), and diffuse astrocytoma, IDH-*mutant* (41.2%). The majority were located in the left temporal lobe (27.8%), followed by the left frontal lobe (25%). Clinical, cognitive, and emotional symptoms information were collected from patients’ medical reports. Cognitive impairment (30%), seizure (22.2%), headache (16.7%), and emotional complaints (11.1%) prevailed as clinical presentation. Cognitive symptoms included deficits of memory (38.9%), language (25%), attention (13.9%), apraxia (8.3%), and executive function (8.3%).

**Table 2:** Preoperative data.

Variable	Mean	Median
Age (years)	45.3	41.0
Educational level (years)	15.5	16.0
	*n*	%
Tumor type (WHO Classification 5th ed.)		
Astrocytoma, IDH-mutant	14	41.2
Astrocytoma, IDH-mutant (grade II)	9	64.3
Astrocytoma, IDH-mutant (grade III)	1	7.1
Astrocytoma, IDH-mutant (grade IV)	2	14.3
Glioblastoma, IDH-wildtype	15	44.1
MGMT promoter methylation	5	33.3
Oligodendroglioma, IDH-mutant, 1p19q codel	5	14.7
Tumor site		
Right frontal lobe	1	2.8
Left frontal lobe	9	25.0
Left fronto-temporal	1	2.8
Insula	4	11.1
Left occipital lobe	1	2.8
Left parietal lobe	8	22.2
Right temporal lobe	2	5.6
Left temporal lobe	10	27.8
Signs and symptoms		
Auditory deficit	1	2.8
Hyposmia	1	2.8
Headaches	6	16.7
Seizure	8	22.2
Motor deficit	3	8.3
Sensory deficit	1	2.8
Nausea/vomiting	2	5.6
Asymptomatic	13	36.1
Preoperative cognitive deficits		
Language	9	25.0
Memory	14	38.9
Executive function	3	8.3
Praxis	3	8.3
Attention	5	13.9
Asymptomatic	15	41.7
Emotional symptoms		
Anxiety	2	5.6
Irritability	2	5.6
Asymptomatic	32	88.9

The average application time for the full test was 23 min, with a minimum of 14 min and a maximum of 35 min.

### Evaluation of the different domains of cognitive tasks proposed by the OMFTCT protocol

**Figure 1:**
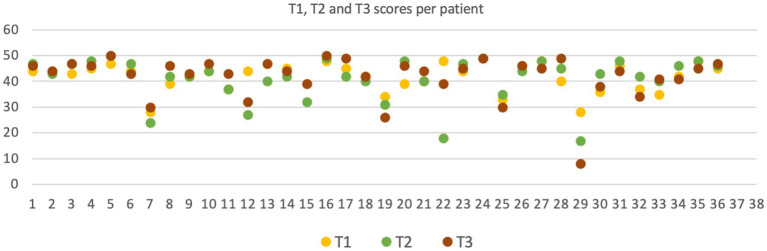
OMFTCT scores analysis at T1, T2 and T3.

Figure 1 shows the cognitive scores at T1, T2 and T3. At T1, the median score was 43. Most of the cohort (75th percentile) achieved scores higher than 47, indicating an excellent cognitive performance. Comparisons between the three assessment times were performed, and no significant differences were found (*p* = 0.417). Dichotomization of the scores, with a score of 40 or more considered good, did not alter the result (*p* = 0.717).

### OMFTCT related to tumor location

**Figure 2:**
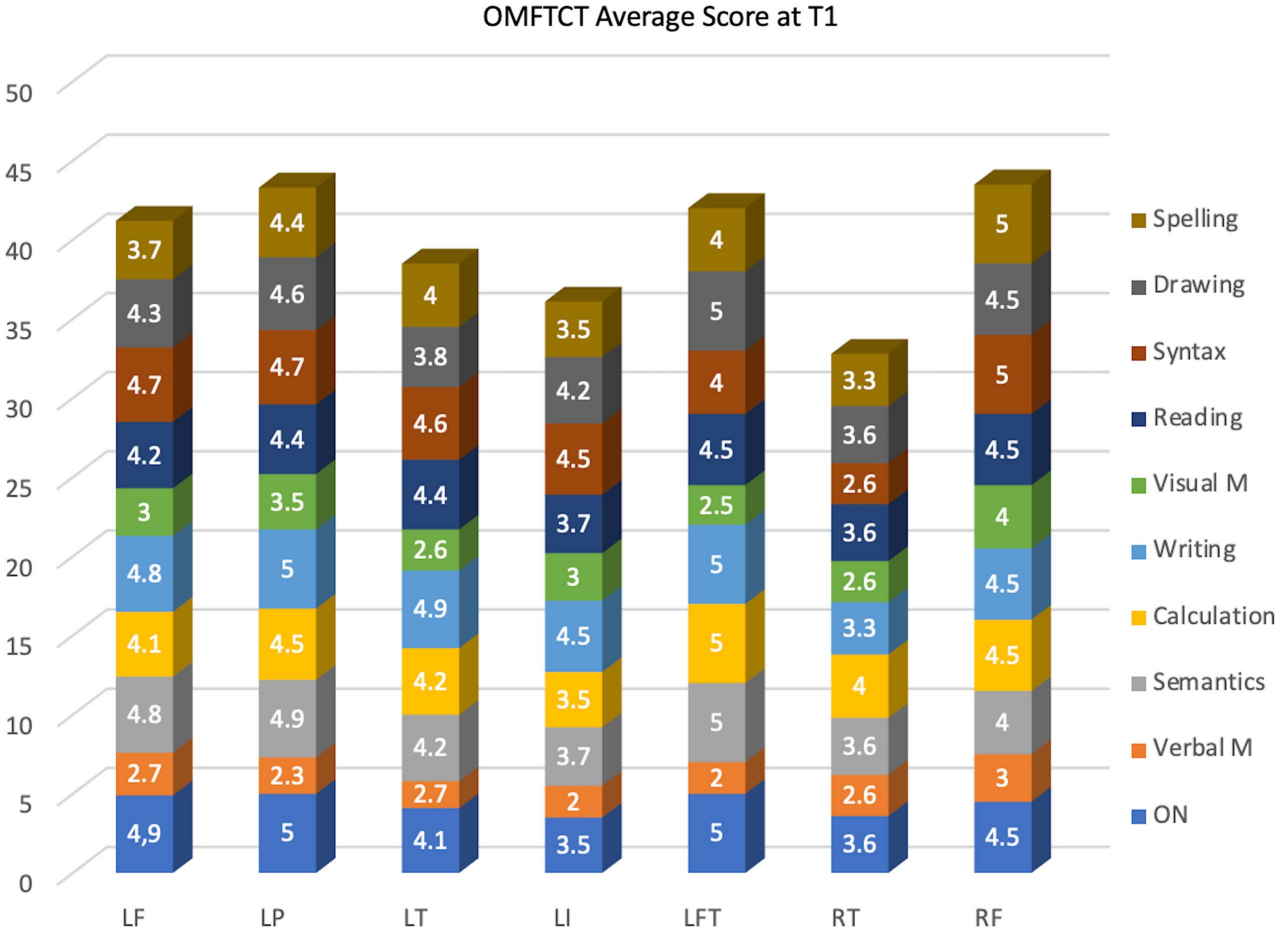
Average OMFTCT scores in each subtest during the preoperative period, categorized by tumor location. LF: left frontal lobe; LP: left parietal lobe; LT: left temporal lobe; LI: left insula; LFT: left frontotemporal region; RT: right temporal lobe; RF: right frontal lobe.

Figure 2 shows a graphic representation of the average OMFTCT scores recorded preoperatively, categorized by tumor location. Object naming and semantics were mostly affected in patients with insular tumors, while verbal and visual memory were predominantly affected in patients with temporal lesions. Interestingly, patients with right temporal lesions (2 patients) exhibited notably low average scores.

## Discussion

Neurocognitive deficits are common among individuals diagnosed with low-grade glioma, due to the adjuvant treatment and the slow tumor growth (van Kessel et al., 2017). Most frequently, executive functions (higher-level cognitive processes responsible for planning, organizing, and problem solving) and memory are involved (van Kessel et al., 2019). Hence, it is essential to diagnose previous cognitive deficits to correctly select patients to undergo awake craniotomy. The patient should be able to effectively communicate, perform the requested task, and report any experienced discomfort. Additionally, individuals should maintain the ability to retain instructions during surgery and preserve visual abilities, particularly when performing tasks involving object naming and semantics (Szelényi et al., 2010). Therefore, a straightforward preoperative neurophysiological assessment is fundamental for evaluating these cognitive domains before awake surgery and tracking the results postoperatively.

When choosing the neuropsychological assessment, an important practical consideration in both research and clinical settings is the trade-off between specificity and brevity (Lageman et al., 2010; Wefel et al., 2011; Dwan et al., 2015). Given that individuals with brain tumors often experience fatigue and psychological distress, administering a lengthy test battery not only burdens the individual but also risks compromising the validity of the test results (Dwan et al., 2015). Hence, it is crucial to strike a balance between specificity and brevity in neuropsychological testing to ensure the generation of valid and meaningful information (Dwan et al., 2015). OMFTCT could be fully applied within a maximum of 35 min and was easily conducted at the patient’s bedside.

Naming ability plays a pivotal role in surgical protocols due to its association with different language areas distributed across different brain lobes and tracts. Yet, naming is limited to language production domain and cannot identify semantic or executive function deficits, for example. Therefore, OMFTCT was designed to encompass additional cognitive domains typically assessed in more comprehensive neuropsychological evaluations. Subtests were designed to reduce application time and minimize patient fatigue. Conducting a more extensive preoperative diagnosis enables safer and more comprehensive intraoperative testing. In our practice, we do not test intraoperatively a task that has not been checked at T1 because of the higher risk of false-positive mapping. Yet, there is a lack of published data suggesting the optimal preoperative testing score for ensuring safe intraoperative assessment. Berger and colleagues. Have suggested that a 25% error rate is acceptable for safe intraoperative testing (Gogos et al., 2020; Al-Adli et al., 2023). In our practice, we test functions with previous OMFTCT scores in the range of 3 to 5. Clearly, untested functions are more susceptible to damage during tumor resection.

The optimization of the intraoperative language protocol is crucial because awake testing normally lasts 1.5 to 2 h before the patient becomes distressed and fatigued. Therefore, it should be tailored to the most probable functions in the vicinity of the manipulated brain area, and the previous patient’s ability to perform that function. The intraoperative tests selection is determined by 1) anatomical planning; 2) neurosurgeon’s interpretation of neuronavigation system during resection; 3) patient’s performance in preoperative cognitive assessment, and 4) tester experience in applying different tasks. Patient’s symptoms point to probable structures affected by tumor growth. Pre-operative cognitive testing identifies impaired function, defining tasks that can be safely assessed intraoperatively. In our practice, we test tasks that attend criteria 1 and 2 and that have scored at least 3 points, translating 30% deficit. It is easy to perform tests that have scored 4 or 5 but it can be challenging with score 3. Score 3 means that a significative amount of function is compromised and that there may be a high risk for intraoperative further damage. Yet, it is expected 30% error rate when performing that test, needing an experienced tester to identify the increase in the error ratio during intraoperative assessment. Errors that become more frequent point to eloquent structure in the vicinity of resection site.

OMFTCT cognitive domains included language, memory, attention, executive functions and praxis, which are commonly assessed in a complete neuropsychological evaluation. The selected tasks were capable of indirectly analyzing other associated functions (Table 1). It is known in neuropsychology that limiting the test to the specific impaired function could lead to a bias and result in misdiagnosing cognitive deficits (Costa et al., 2018). For example, if a patient with temporal brain tumor is tested only for semantics, memory deficits could go unrecognized (Bosma et al., 2008; Blonski et al., 2012). Therefore, OMFTCT aimed to evaluate different cognitive domains to identify different impairments. Additionally, the tasks presented a medium degree of difficulty, demanded short execution time and encompassed direct and indirect cognitive assessment. The overall analysis process was based on measures described by Giovagnoli (2012).

Response time in neuropsychological tests is a critical measure for assessing cognitive function, including processing speed, attention and executive function. Response time can vary widely depending on the test applied. Changes in response time can indicate cognitive difficulties that require intervention. To assess processing speed, using Trail Making Test (TMT), the average time to complete tasks A and B varies from 20 to 90 s (Guo, 2022). However, patients with glioma may show significantly longer times due to impaired processing speed and cognitive flexibility (Taphoorn and Klein, 2004).

For the Stroop Test, which assesses attention and executive function, the average execution time ranges from 1 to 3 s but patients with glioma may show prolonged response times, indicating difficulty in cognitive inhibition and selective attention (Taphoorn and Klein, 2004; Scarpina and Tagini, 2017). For verbal and working memory tests, such as the Digit Span (Wais Subtest), the immediate response is considered for direct order and a few seconds longer for reverse order due to the greater processing load. Patients with glioma have prolonged response times, indicating difficulty with working memory and information processing (Tucha et al., 2000).

Patients with glioma can show a significant reduction in the number of words and a longer hesitation between the words during verbal fluency tests (Tucha et al., 2000; Robinson et al., 2006). To evaluate and interpret results in glioma patients, it is important to analyze response times, strategies used by patients (for example, circumlocution), errors and behavior during the test (Pilarska et al., 2023). In the OMFTCT test, we measured the total application time instead of the response time for each subtest because for most of them there is no standard. Yet, we used the limit of 20 s for naming test because it is the current measure (Bilotta et al., 2014). Slowing of responses, reverberation, and phonemic paraphasias were described as qualitative aspects.

Currently, periodic neuropsychological assessments can identify changes in response time and are fundamental to adjust care plans (Klein et al., 2002; Meyers and Brown, 2006). It enhances interpretation of the scores within the patient’s individual clinical context aiming to improve early diagnosis and rehabilitation (Dwan et al., 2015). Interestingly, during intraoperative testing, the development of slowing can be identified as progressive prolongation of the response times.

Among the cognitive symptoms observed in the preoperative period, memory deficits were the most common, affecting 38.9% of the sample. In general, glioma patients receiving treatment are likely to experience medium to long-term deficits in the memory and attention domains (van Kessel et al., 2019). Of note, OMFTCT could easily identify visual memory impairment. Among the 36 evaluated patients, 20 exhibited visual memory deficits, with most of these lesions located in the left hemisphere. This characteristic should be checked in a future validation process in a larger sample.

OMFTCT protocol provides a significant practical advantage by not demanding the mandatory presence of a specialized neuropsychologist for its application. The testers could be neurosurgeons or neurosurgery residents or fellows since they have the expertise needed to recognize anomia, speech arrest, paraphasias, reverberation, etc. Those clinical errors are within the scope of neurological training. Furthermore, qualitative analysis has shown consistent results across different testers. These attributes render the test highly reliable, facilitating preoperative cognitive assessments even in facilities lacking specialized neuropsychologists. OMFTCT could be particularly useful in hospitals and centers that cannot count with a neuropsychologist, especially in Latin America. The protocol is user-friendly and can be administered at the patient’s bedside.

Yet, OMFTCT was developed by a neuropsychologist and the team could rely on her for training, doubts and scores qualitative analysis. Besides, the neurosurgeon group where it was developed is a glioma dedicated team, with experience in awake intraoperative mapping. We believe that it could have influenced the results. Therefore, it is advisable to count with neuropsychologists with expertise in cognitive assessment, to build a learning curve in the overall management of cognitive assessment in the context of awake craniotomy. Finally, the neuropsychologist can assess and address any emotional issues related to the illness, establishing a strong rapport with the patient, ultimately enhancing adherence to cognitive rehabilitation.

As the preservation of neurocognitive function plays a crucial role in quality of life, it has become the primary goal in surgical approaches for glioma patients (Wolf et al., 2016; Altieri et al., 2019; Chen et al., 2020; Schiavolin et al., 2020; Parsons and Dietrich, 2021; Sierpowska et al., 2022). Therefore, preoperative impairment diagnosis and consequent accurate intraoperative mapping are milestones and enhance the safety of the procedure (Dwan et al., 2015). The severity and type of cognitive impairment play crucial roles in quality of life of individuals with glial tumors (Noll et al., 2015; Gasa-Roqué et al., 2024). Acquiring precise information about a person’s neuropsychological condition is essential for designing appropriate rehabilitation and supportive care plans (Ownsworth et al., 2009; Dwan et al., 2015). Neuropsychological functioning has been shown to have implications for prognosis and tumor recurrence, and it may even be more sensitive than imaging techniques for predicting early tumor recurrence (Armstrong et al., 2003; Meyers and Hess, 2003).

## Limitations of the study

The sample was shortened because only 36 patients completed T3. It is worth noting potential biases associated with this issue. Despite of being excluded, we correlated the T1 score with tumor location (Figure 2).

The lack of comparison of each OMFTCT task with an equivalent validated test. This study was limited to check the feasibility of a new fast-track protocol in recognizing pre- and post-operative cognitive deficits. Yet, we could improve the intraoperative tasks selection based on identified pre-operative OMFTCT and it improved the dynamics of awake craniotomy. Before using OMFTCT, and in the absence of a previous language assessment, the interpretation of intraoperative anomia, circumlocution, paraphasia, etc. were unsafe and stressful. In an extreme context, it may have influenced the extent of resection in many patients.

## Conclusion

The pre-operative cognitive assessment improves the overall management of the glioma patient undergoing awake craniotomy. It enhances intraoperative language testing planning, mightily improving extent of tumor resection. In the post-operative context, it allows recognition of persistent deficits that demand neuropsychological rehabilitation.

The OMFTCT provided pre- and postoperative assessment of a broad variety of cognitive domains, enabling the effective planning of intraoperative cognitive testing. Moreover, immediate diagnosis of neurocognitive deficits plays a crucial role in guiding patient care and rehabilitation. Future studies with larger and more diverse populations, including patients with lower educational levels, may yield more comprehensive data. Future validation will enhance its credibility. Additionally, we aim to give free access online on our group’s website, vemotests.com, to facilitate broader usage in various clinical settings. This will allow other researchers and clinicians to implement and benefit from OMFTCT protocol, further contributing to the field of preoperative cognitive assessment.

## Data availability statement

The original contributions presented in the study are included in the article/Supplementary material, further inquiries can be directed to the corresponding authors.

## Ethics statement

The studies involving humans were approved by Research Ethics Committee of the Sírio-Libanês Hospital (CAEE: 14601619.7.0000.5461). The studies were conducted in accordance with the local legislation and institutional requirements. The participants provided their written informed consent to participate in this study. Written informed consent was obtained from the individual(s) for the publication of any potentially identifiable images or data included in this article.

## Author contributions

JO: Conceptualization, Data curation, Investigation, Writing – original draft. CF: Data curation, Investigation, Methodology, Project administration, Writing – original draft, Writing – review & editing. DG: Data curation, Writing – original draft. JN: Data curation, Methodology, Writing – original draft. MV: Data curation, Investigation, Writing – original draft. LV: Data curation, Investigation, Writing – original draft. GG: Investigation, Writing – original draft. BS: Data curation, Investigation, Writing – original draft. AC: Data curation, Investigation, Writing – original draft. SV: Data curation, Investigation, Methodology, Writing – original draft, Writing – review & editing. MM: Investigation, Methodology, Writing – original draft, Writing – review & editing.
