# Supplementary material for: Filling the gap: brief neuropsychological assessment protocol for glioma patients undergoing awake surgeries
Source: Front Psychol. 2024 Aug 9;15:1417947. doi: 10.3389/fpsyg.2024.1417947 (PMC11342098; doi:10.3389/fpsyg.2024.1417947)
Supplement: Supplementary file 1 [file Data_Sheet_1.PDF]

Protocolo
Avaliação Neuropsicológica

Cirurgia *Awake* – *PRÉ-OPERATÓRIO*

NOMEAÇÃO

1

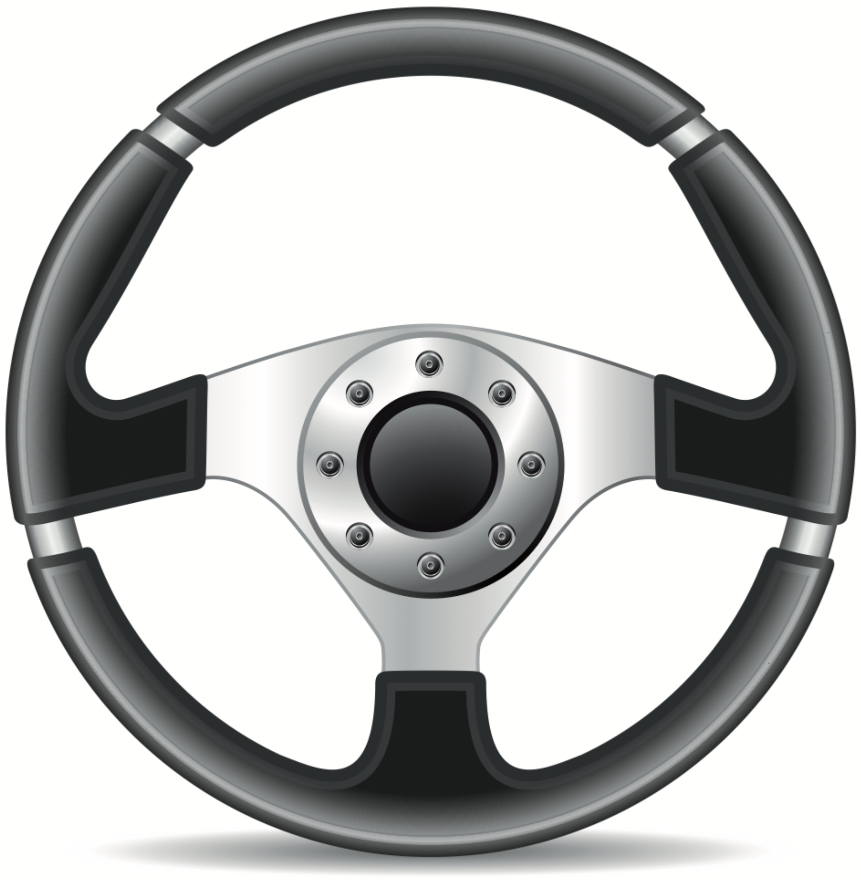

2

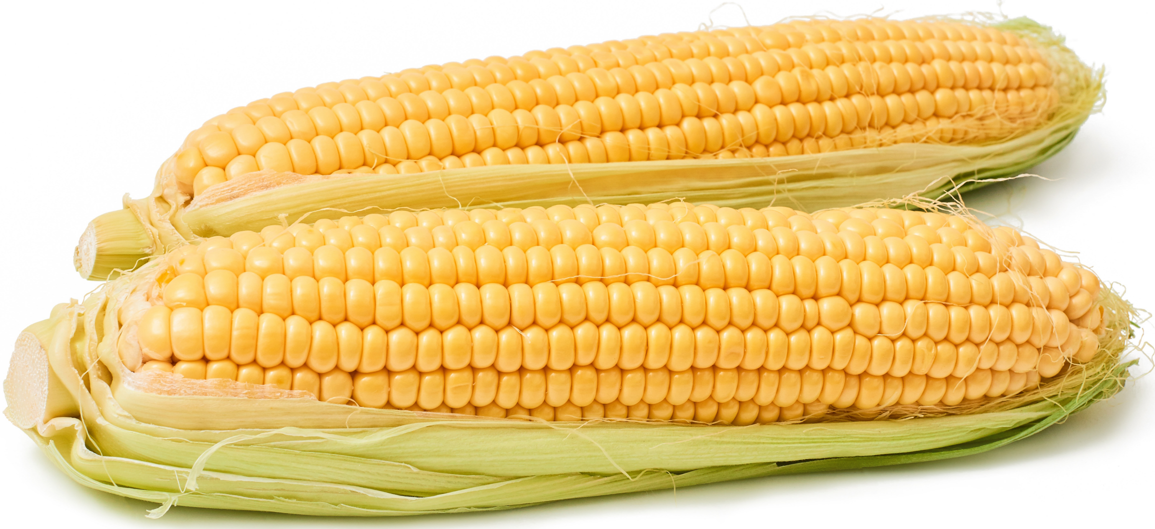

3

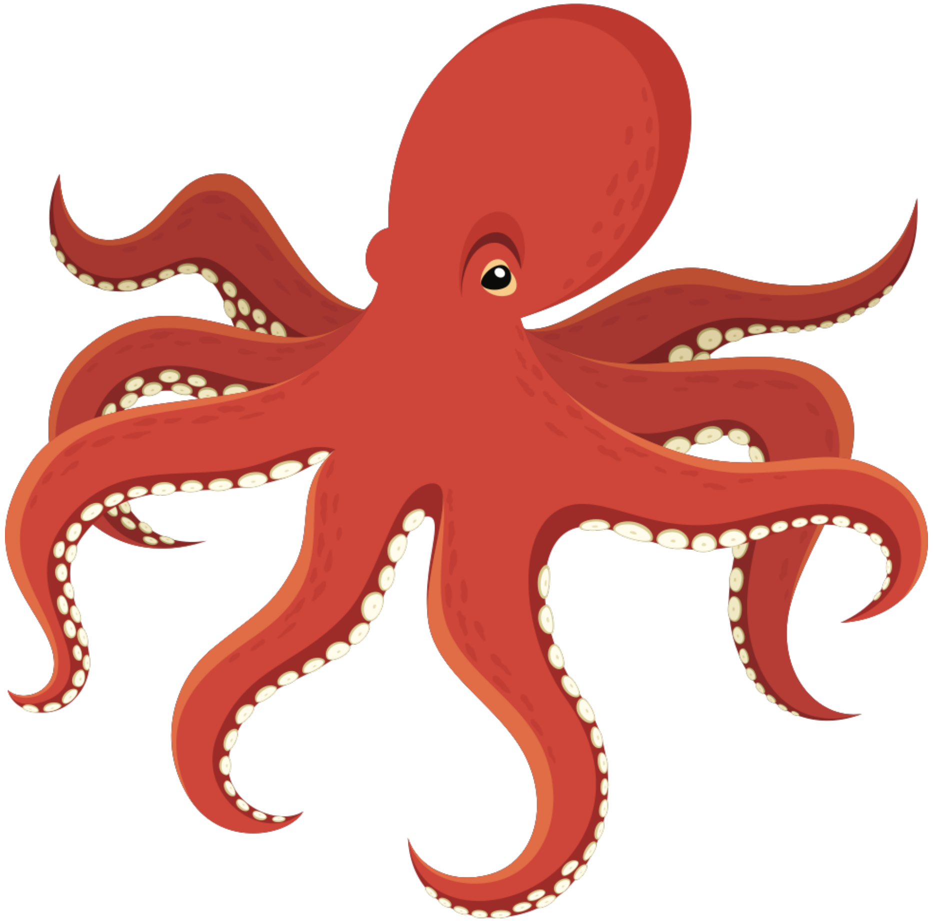

Nomeação com dupla tarefa
(Abrir e fechar as mãos, alternadamente enquanto nomeia a figura)

4

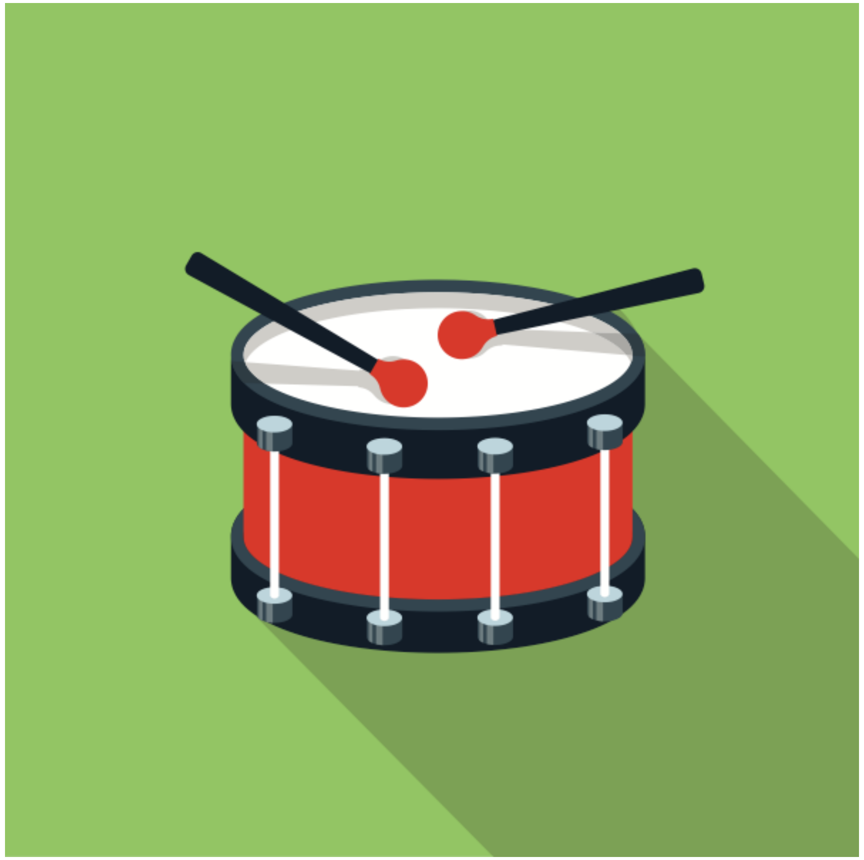

5

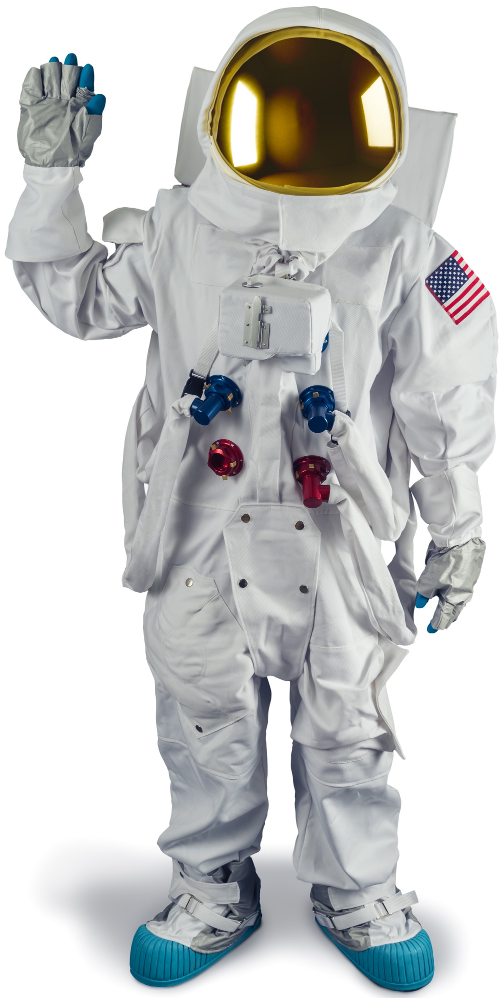

MEMÓRIA VERBAL

Memória Verbal

Memorizar e repetir o que é ditado:

1. Cinza é a cor do rato que entrou na casa amarela.
2. O rato cinza entrou na casa amarela que tinha uma porta vermelha na lavanderia.
3. Sapato – andorinha – ameixa – luva – galho
4. 6 – 0 – 9 – 0 – 3 – 5 – 2
5. Q – J – D – I – V – O – P – W – L

SEMÂNTICA

A figura/ palavra de cima combina com qual figura de baixo?

1. A figura de cima combina com qual figura de baixo?

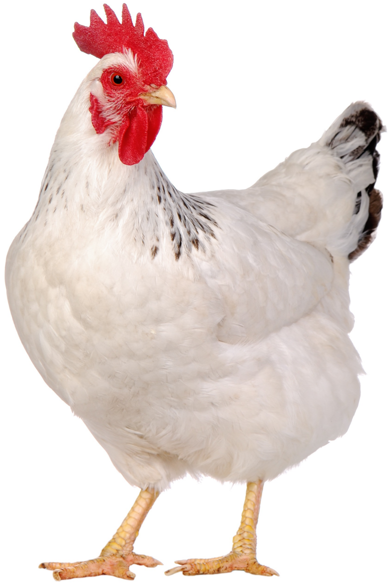

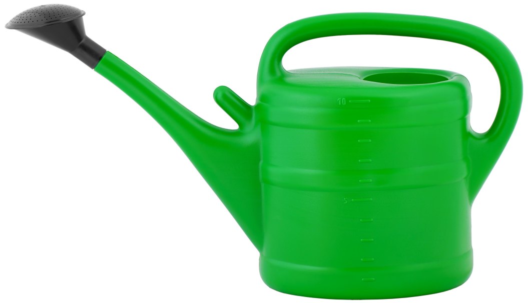

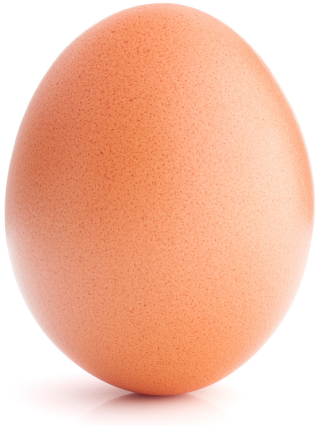

2. A palavra de cima combina com qual figura de baixo?

bombeiro

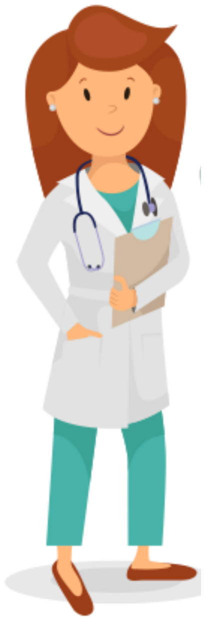

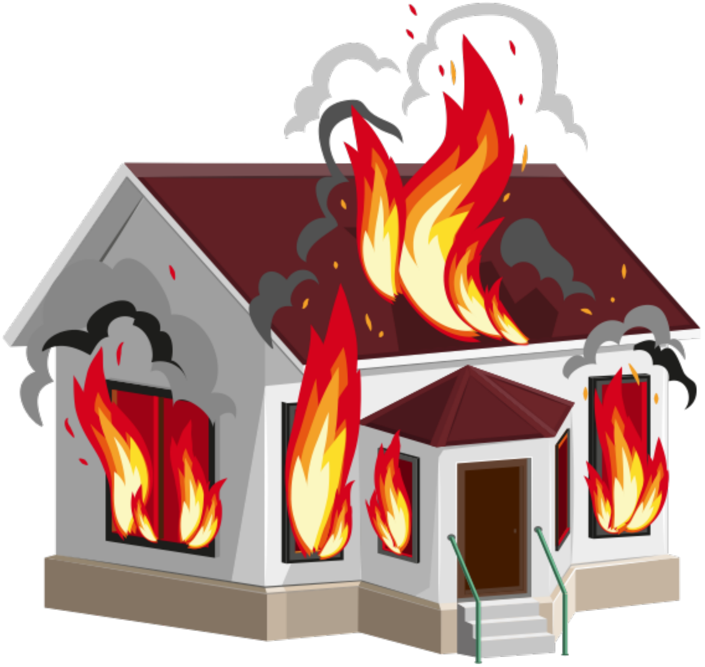

Semântica com dupla tarefa
(Tocar a orelha direita com a mão esquerda e vice-e-versa, alternadamente, enquanto responde o exercício)

3. Para que serve?

PREGO

4. De que material é feito?

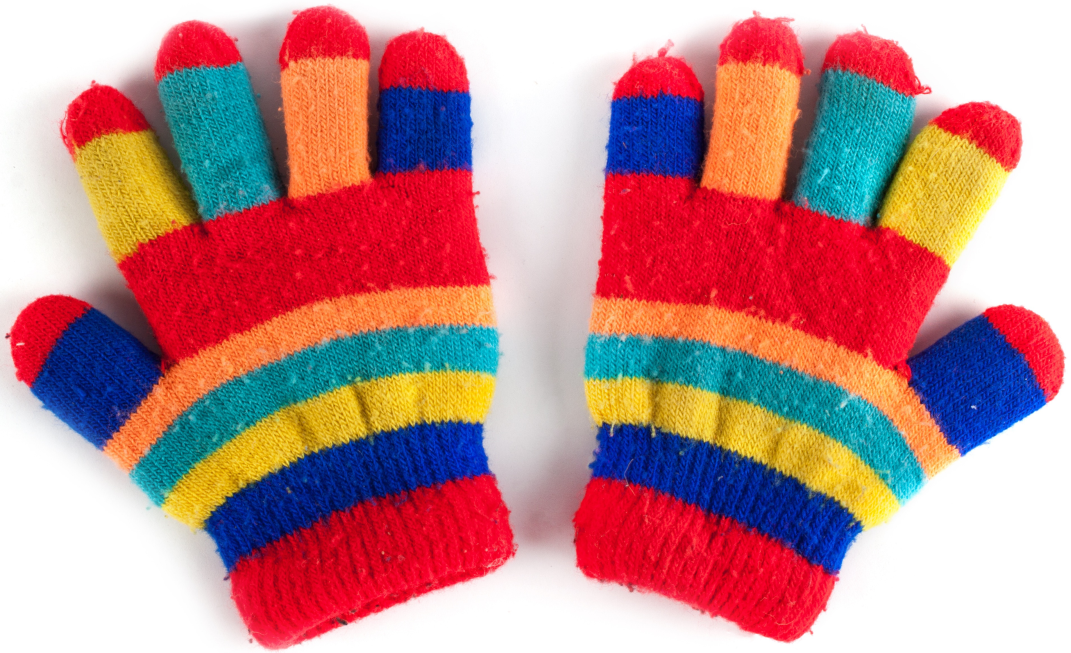

5. Que forma tem?

CANETA

CÁLCULO

CALCULE E DIGA O RESULTADO

1. 5 + 6 =
2. 12 – 7 =
3. 25 x 6 =

4. Dentre a sequência de números a seguir, diga os três números que somados resultam em 13.

5 – 8 – 3 – 10 – 2 – 9

5. Quantas moedas de 0,25 são necessárias para se obter R$ 4,00?

GRAFIA

COPIE AS PALAVRAS NA FOLHA DE RESPOSTA:

1. Analogia
2. Carreira

COPIE A FRASE NA FOLHA DE RESPOSTA:

3. Levou dentro da sacola larga uma tonelada de laranjas.

DITADO

DITADO DE PALAVRA

4. Manutenção

DITADO DE FRASE

5. Não havia outra alternativa a não ser presenteá-lo.

MEMÓRIA VISUAL

Memória Visual

Observe as figuras do quadro A e a seguir, escreva na folha de respostas quais imagens do quadro B apareceram no quadro A

1. Quadro A

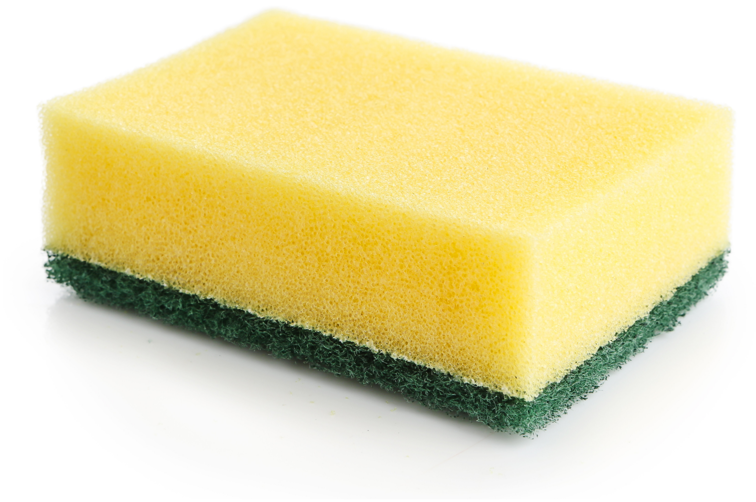

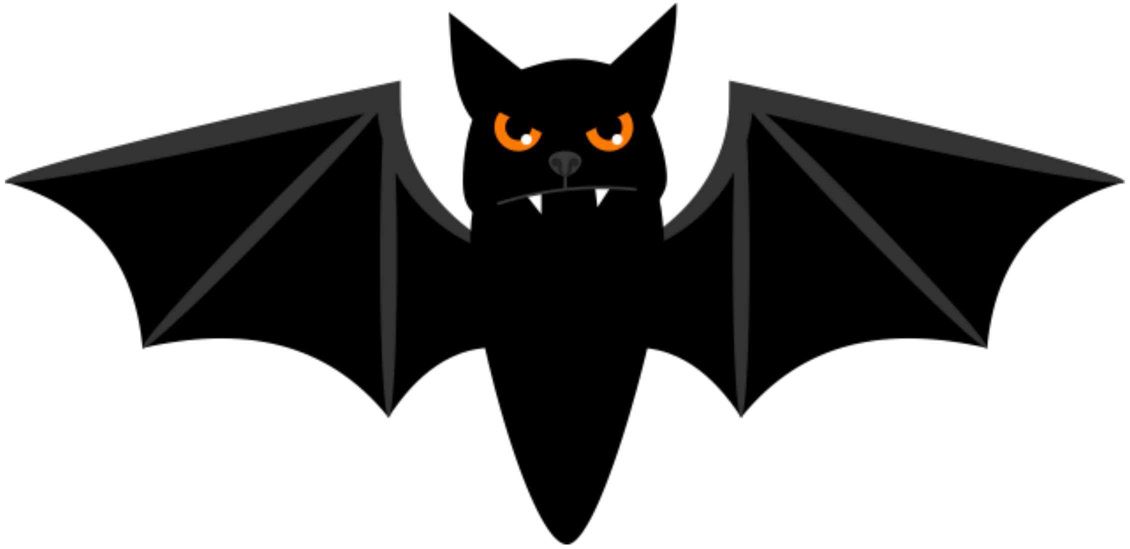

1. Quadro B

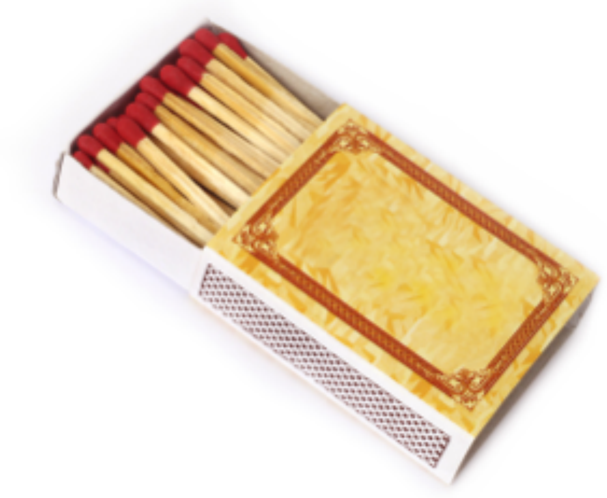

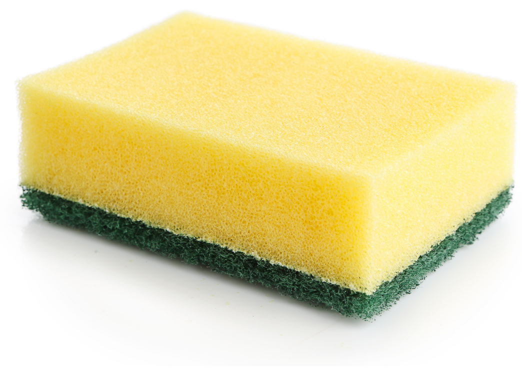

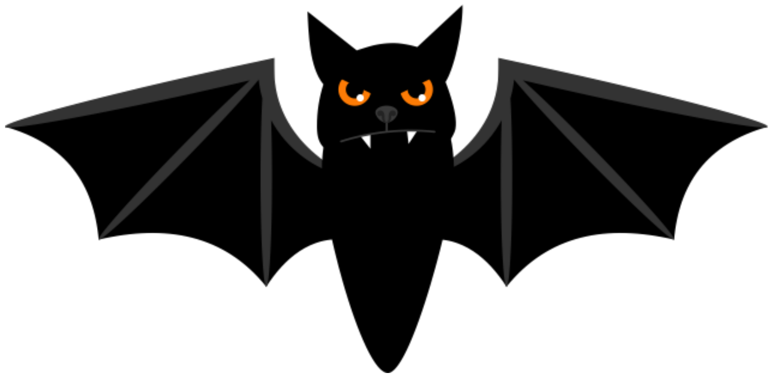

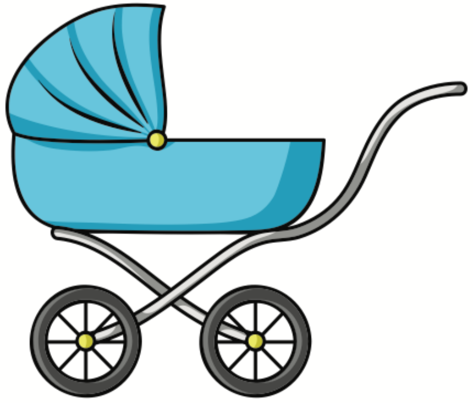

2. Quadro A

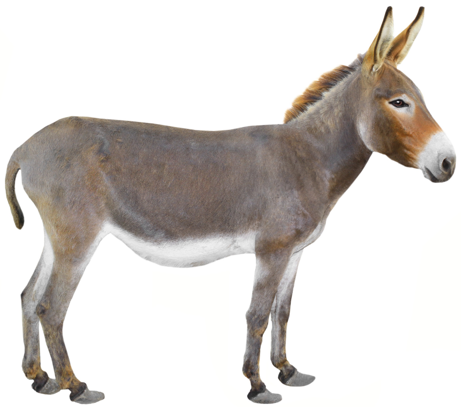

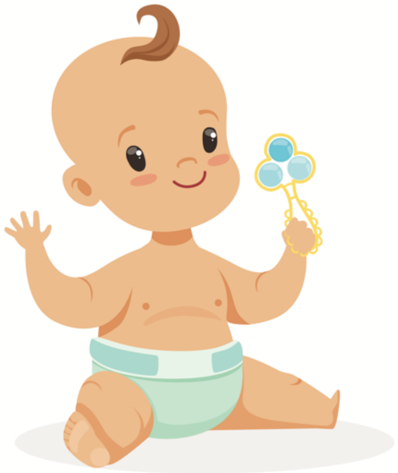

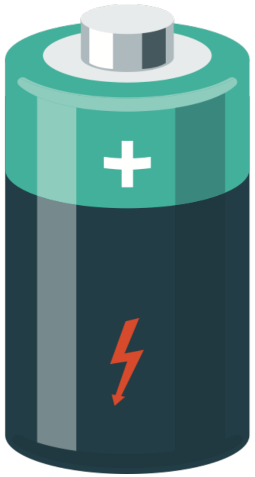

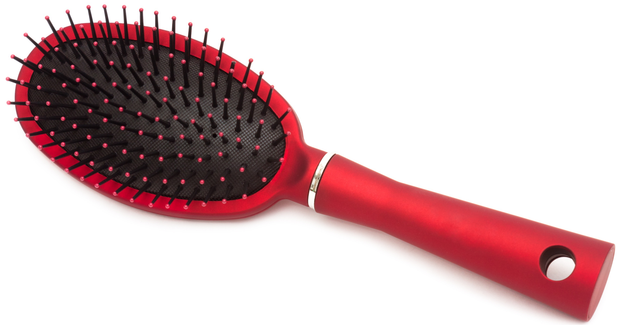

2. Quadro B

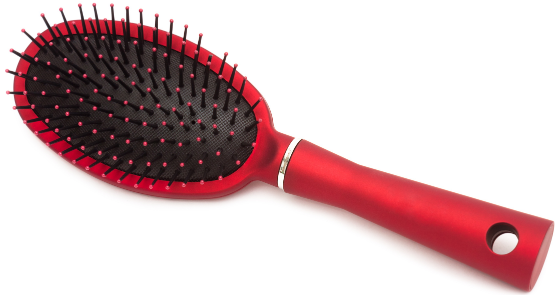
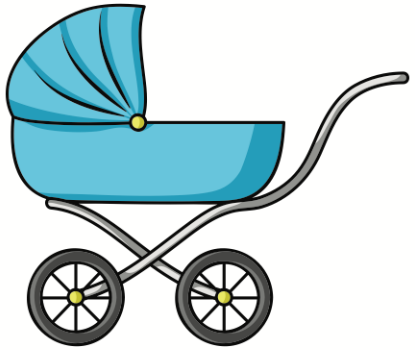
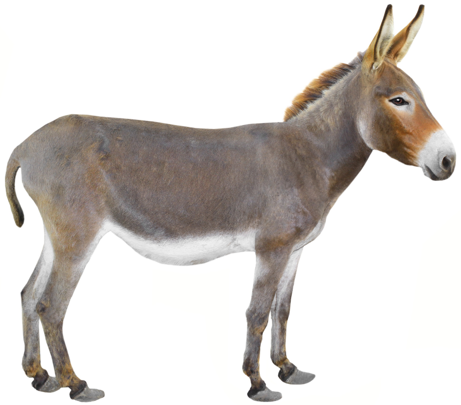
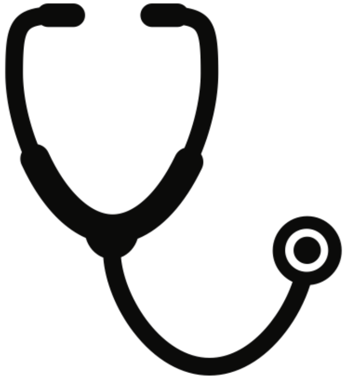
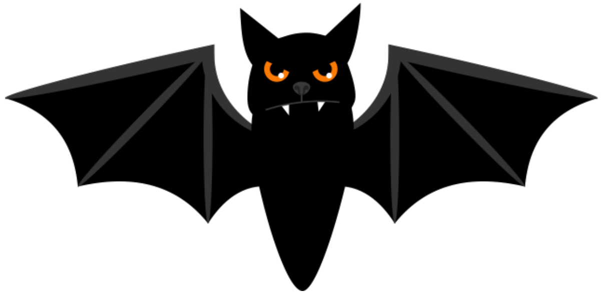
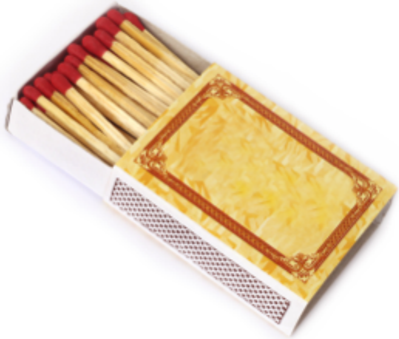
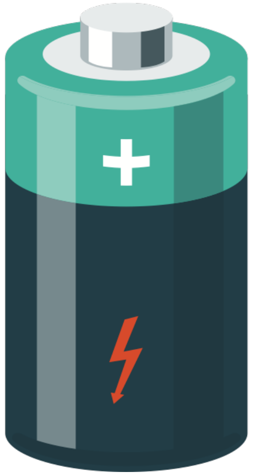
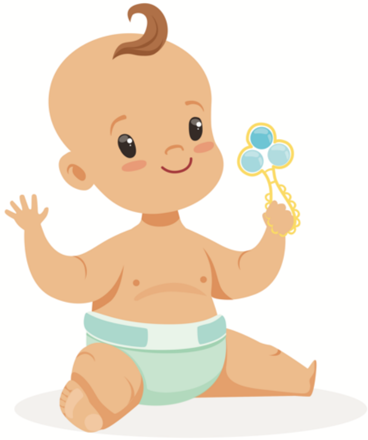

3. Quadro A

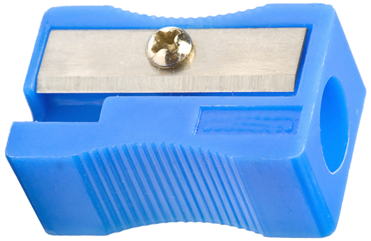

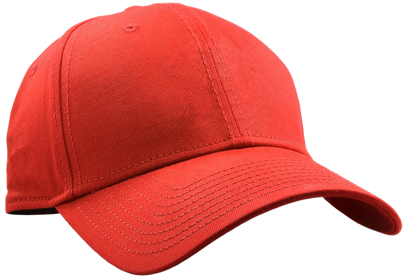

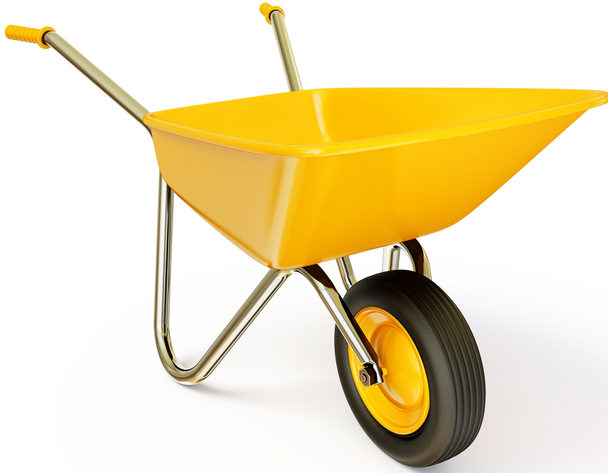

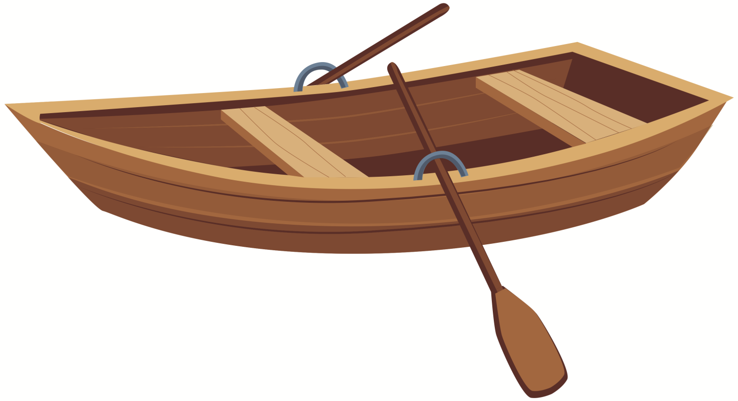

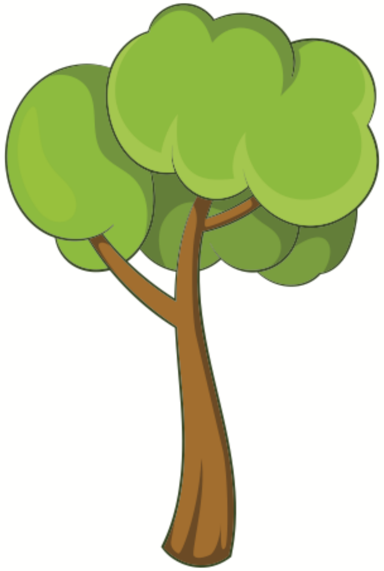

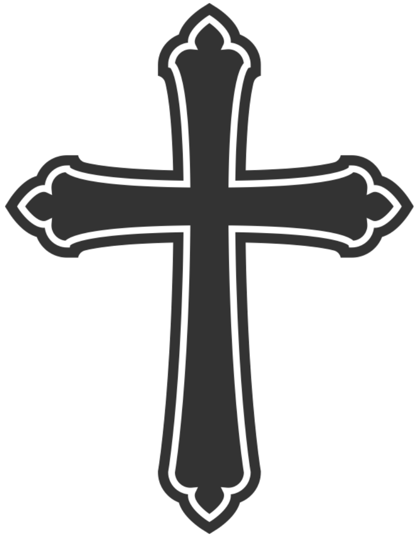

3. Quadro B

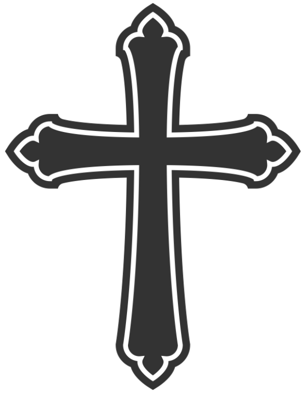
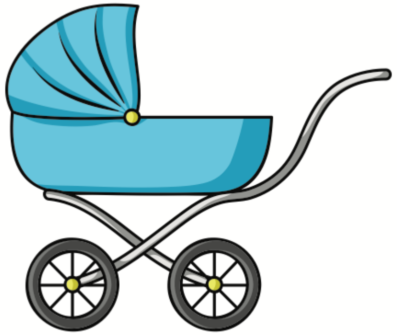
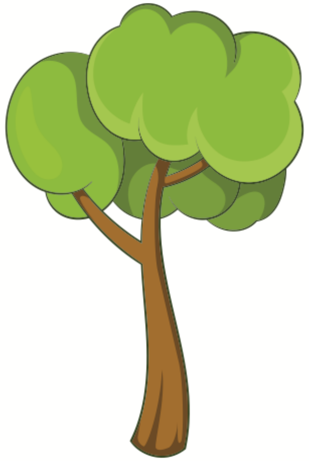
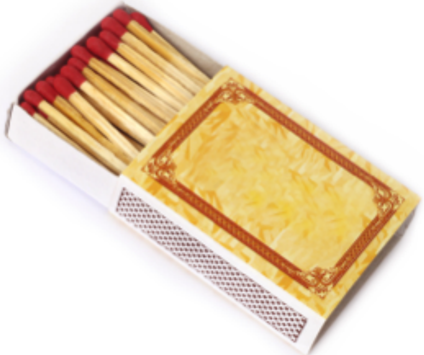
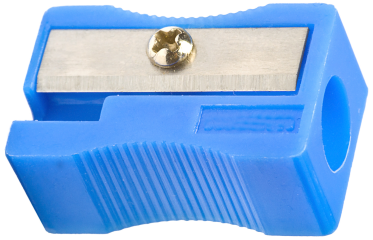
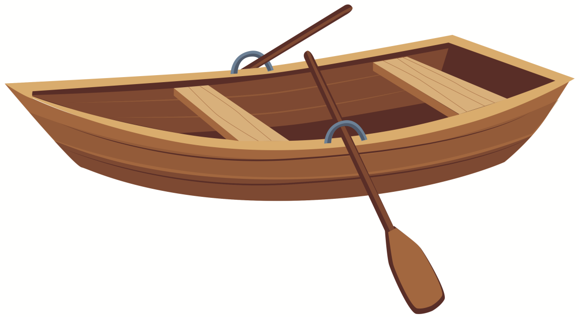
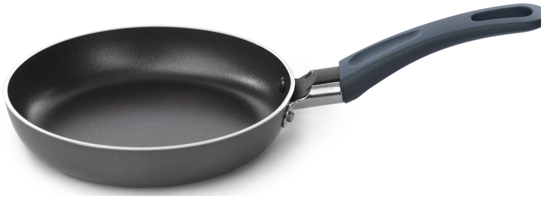
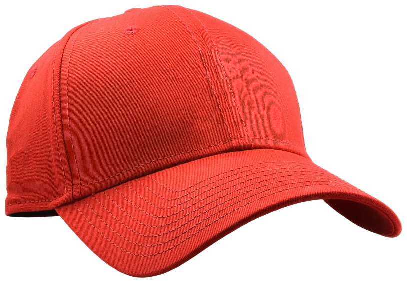
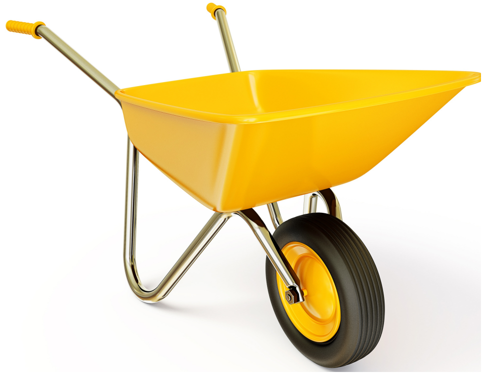

4. Quadro A

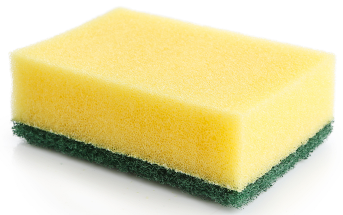
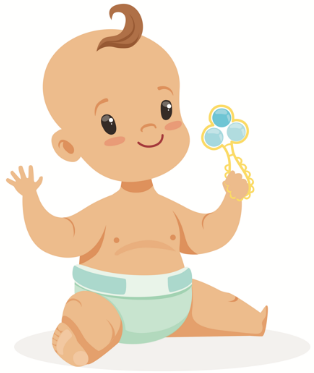
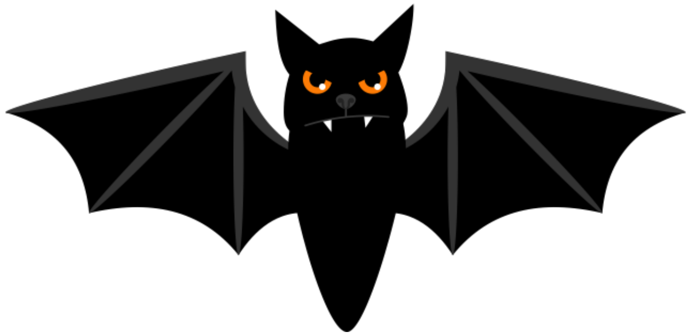
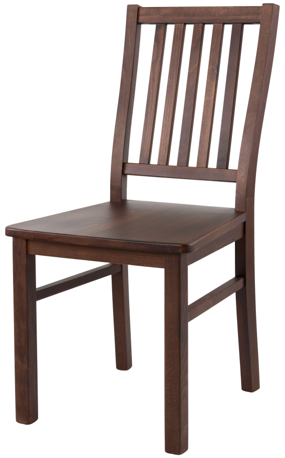
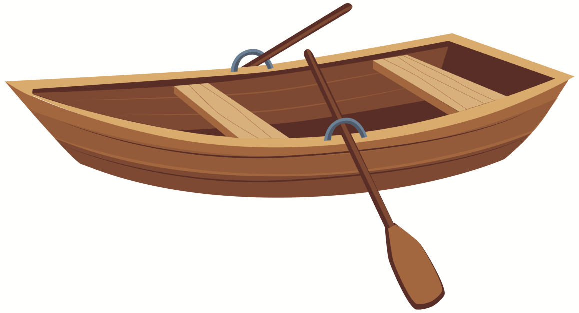
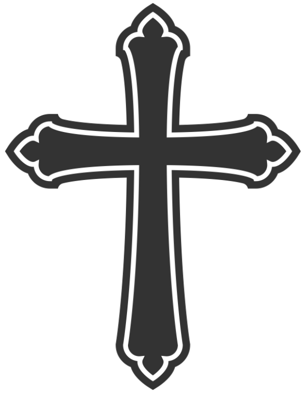
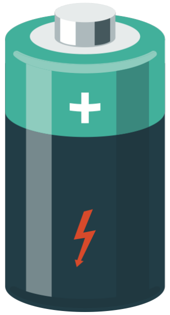
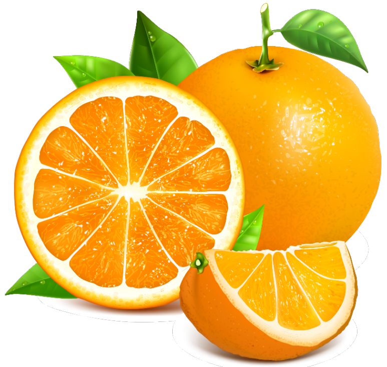

4. Quadro B

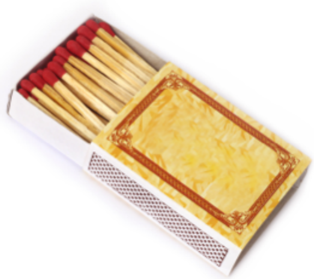
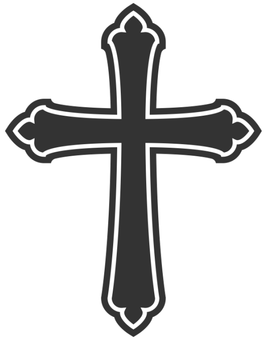
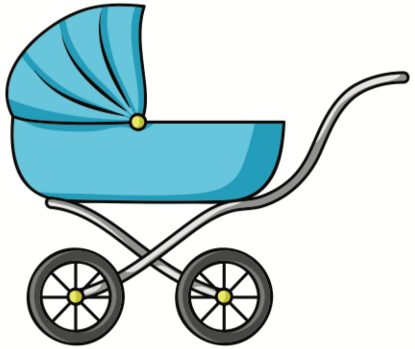
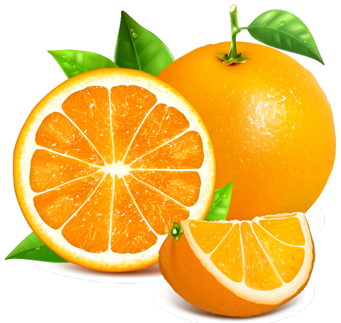
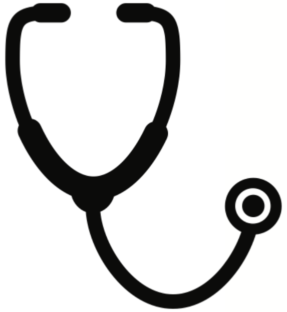
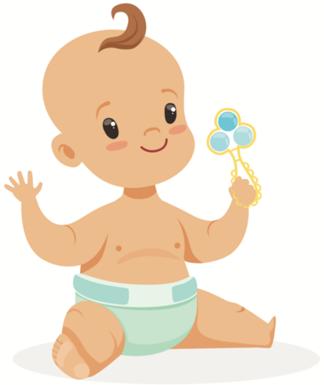
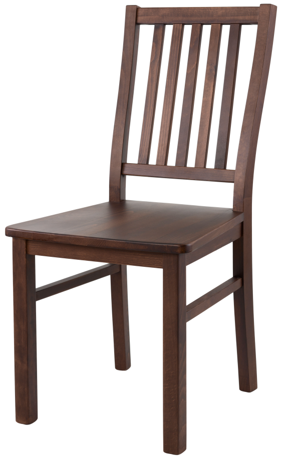
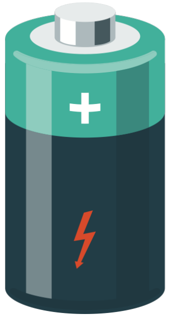
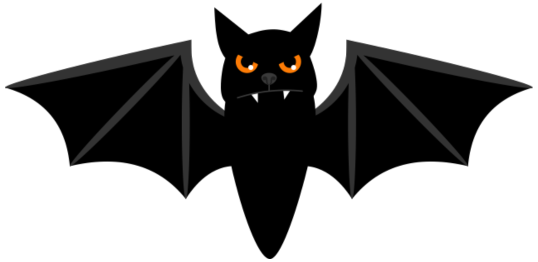
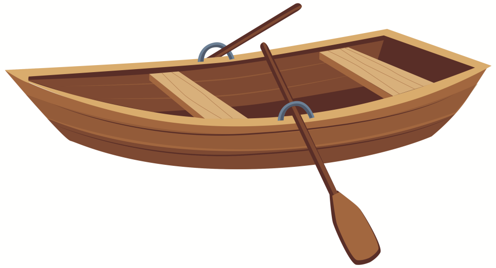
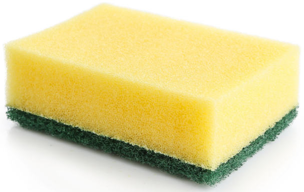

5. Quadro A

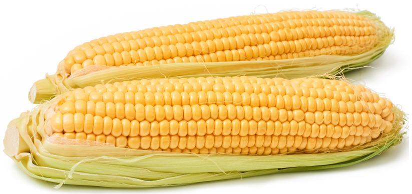
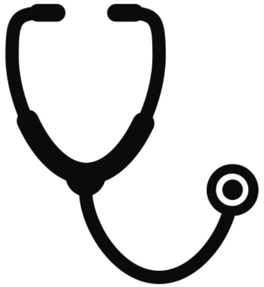
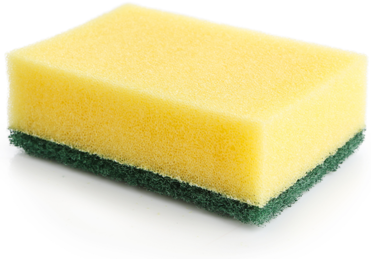
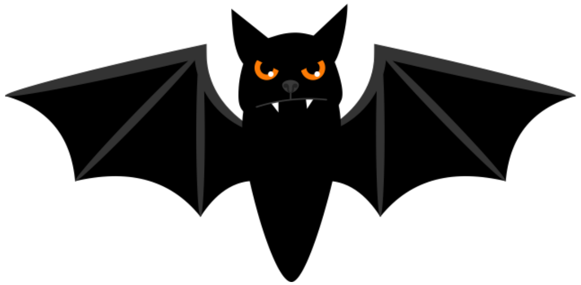
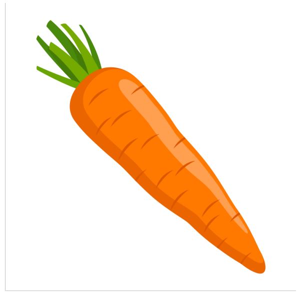
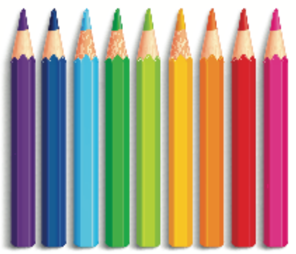
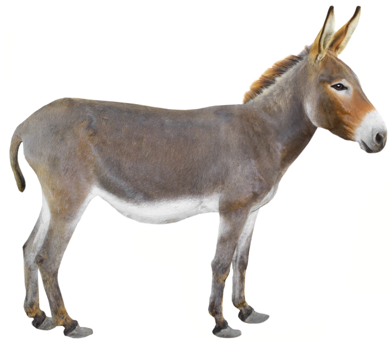
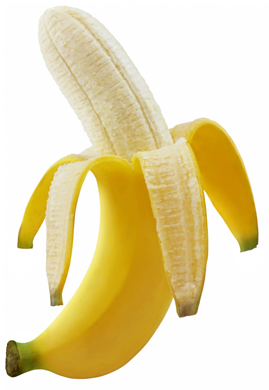
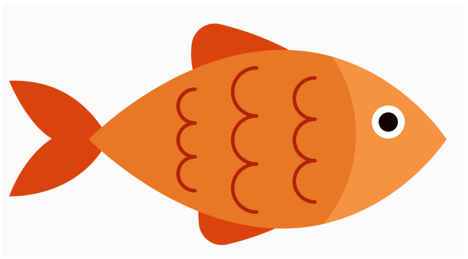
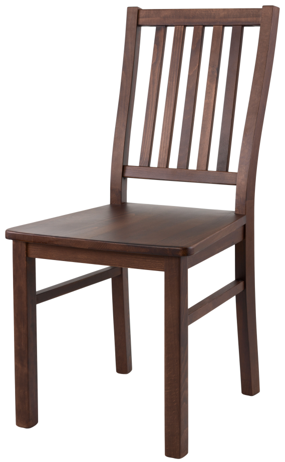

5. Quadro B

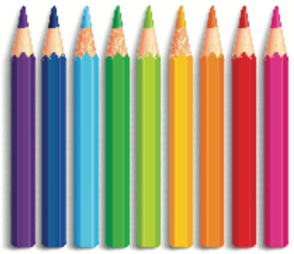
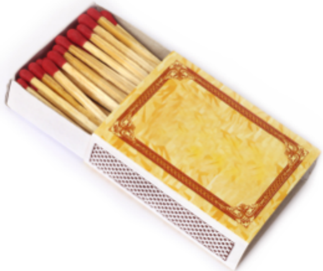
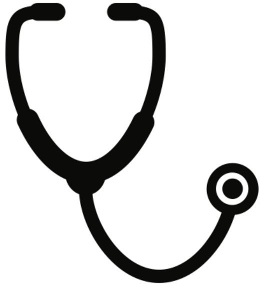
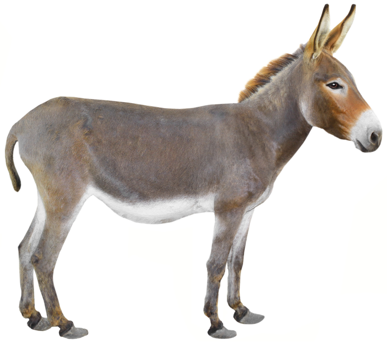
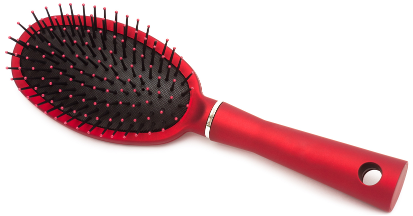
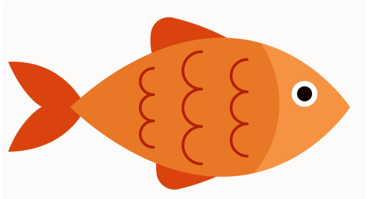
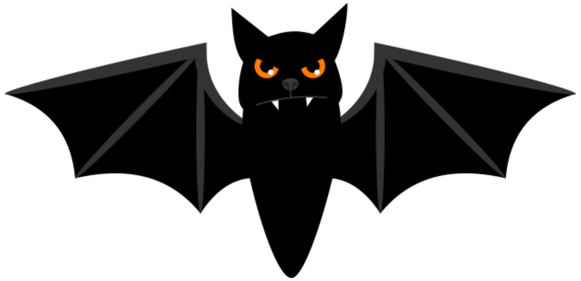
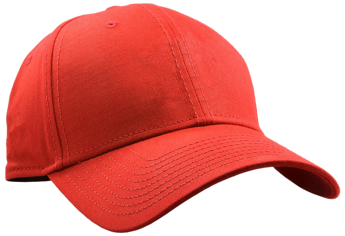
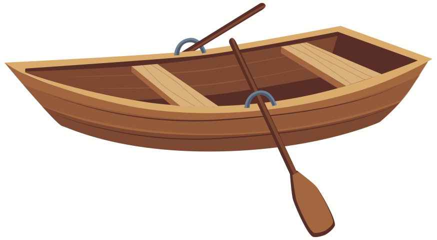
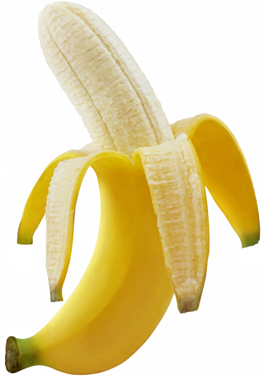
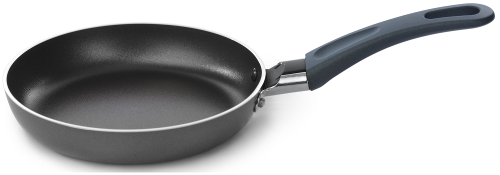
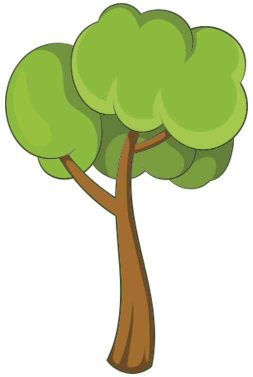
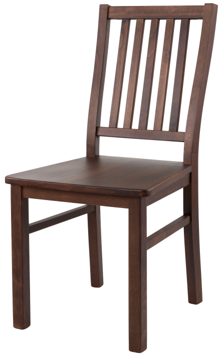
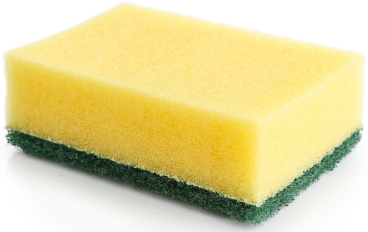
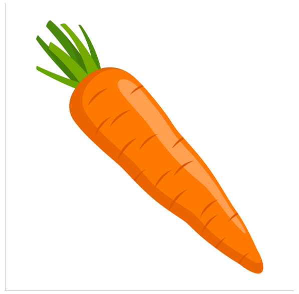
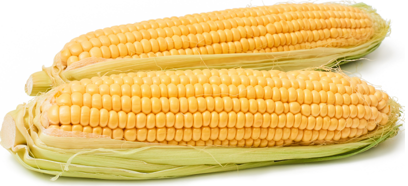

LEITURA E REPETIÇÃO

Leia e repita em voz alta as frases a seguir

1. Teto sujo, chão sujo.
2. Caixa de graxa grossa de graça.
3. Trazei três pratos de trigo para três tigres tristes comerem.
4. A Iara agarra e amarra a rara arara de Araraquara.
5. Quem a paca cara compra, paca cara pagará.

SINTAXE

Sintaxe – Formação de palavras

Descubra a palavra:

1. NO – PLA
2. SOU – TE – RA
3. NE – TE – FO – LE

4. A palavra LEAL, só não se encaixa em:

a) dis b)des c)dade

5. Local onde se compra PAPEL:

DESENHO

Desenhe as formas a seguir, na folha de resposta.

1. Círculo
2. Triângulo
3. Cubo

Copie as formas a seguir, na folha de resposta.

4.

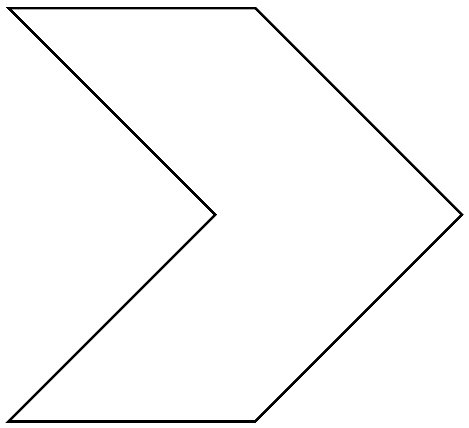

5.

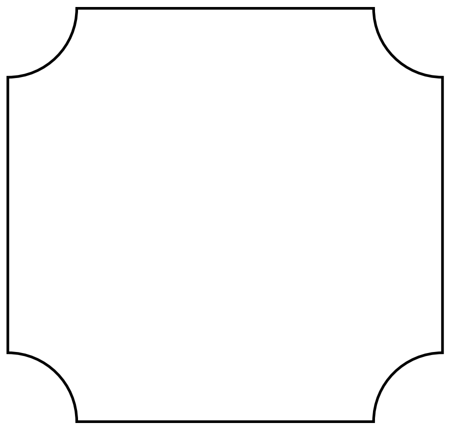

SOLETRAÇÃO

Soletrar na ordem direta

1. ARCO
2. BALEIA
3. SANDUÍCHE

Soletrar na ordem inversa

4. ADORAR
5. ESTREIA
