# Supplementary material for: Filling the gap: brief neuropsychological assessment protocol for glioma patients undergoing awake surgeries
Source: Front Psychol. 2024 Aug 9;15:1417947. doi: 10.3389/fpsyg.2024.1417947 (PMC11342098; doi:10.3389/fpsyg.2024.1417947)
Supplement: Supplementary file 3 [file Data_Sheet_3.PDF]

Protocolo
Avaliação Neuropsicológica

Cirurgia *Awake – PÓS 30 DIAS*

NOMEAÇÃO

1

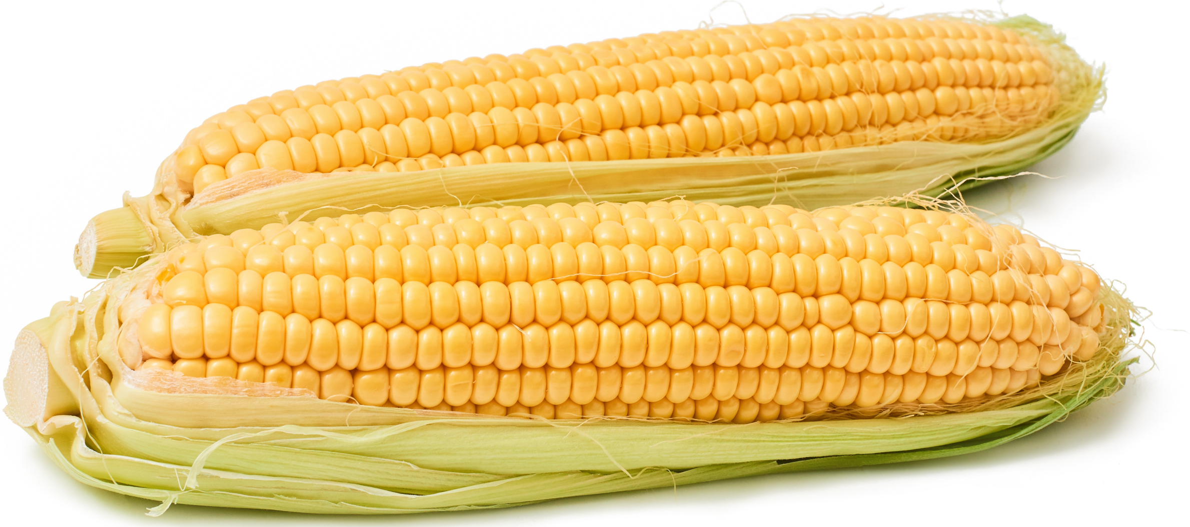

2

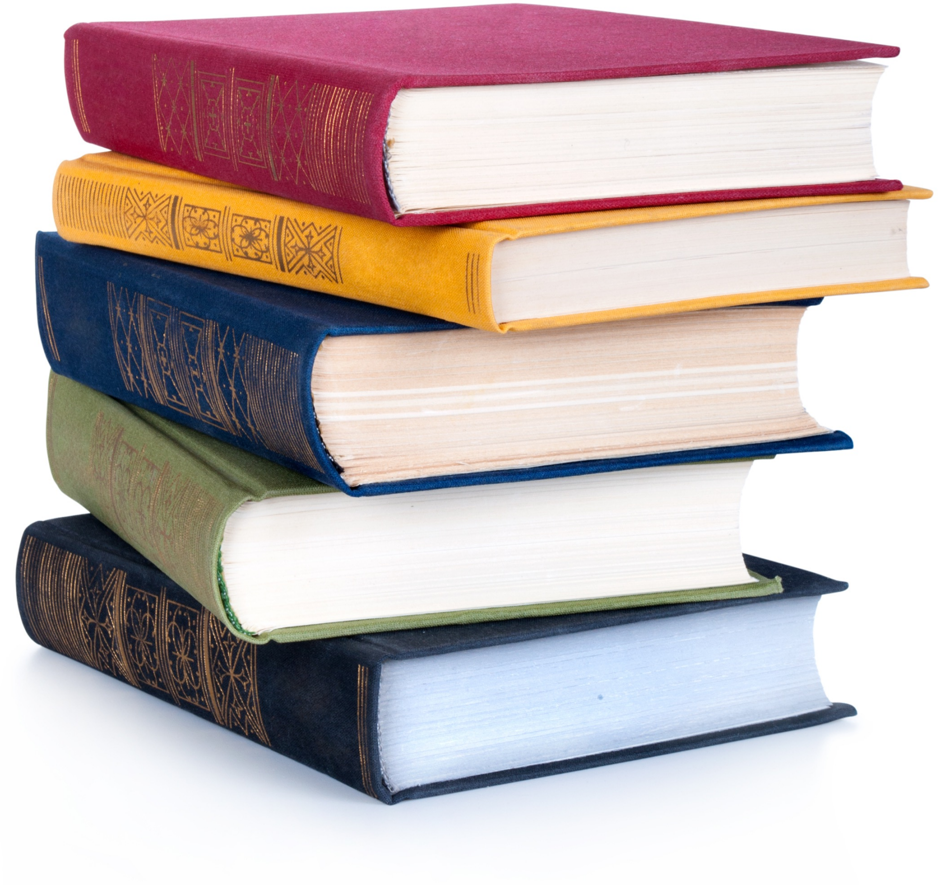

3

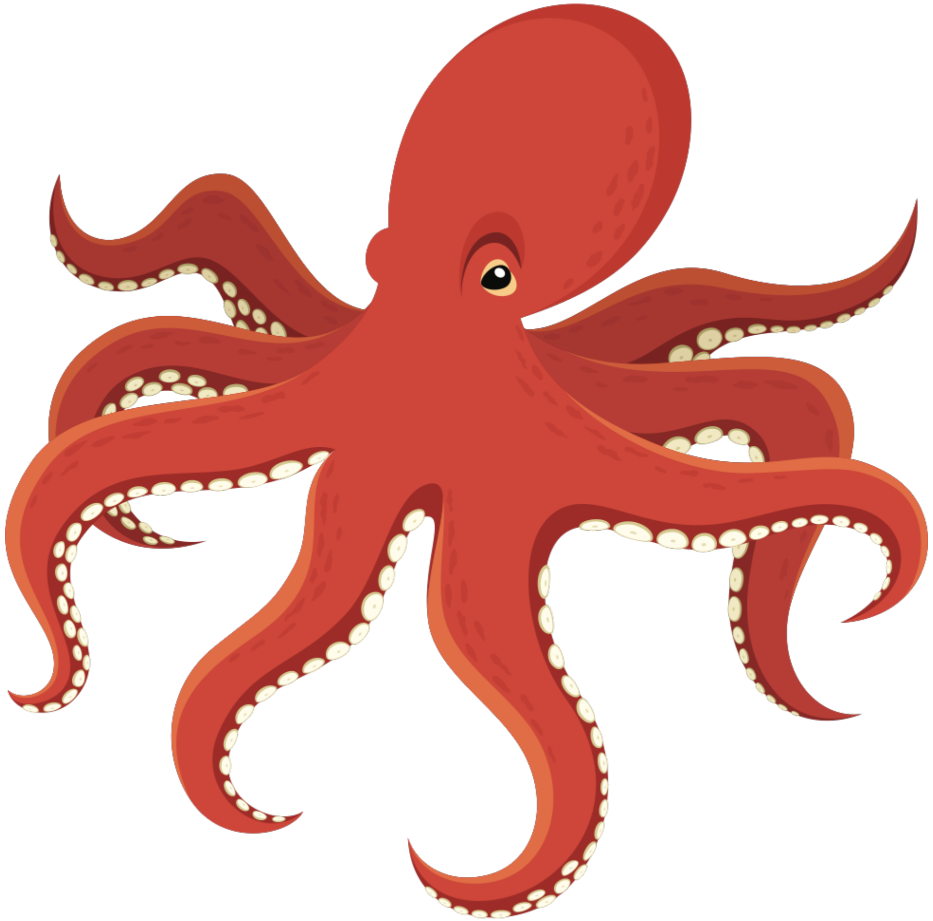

Nomeação com dupla tarefa
(Abrir e fechar as mãos, alternadamente enquanto nomeia a figura)

4

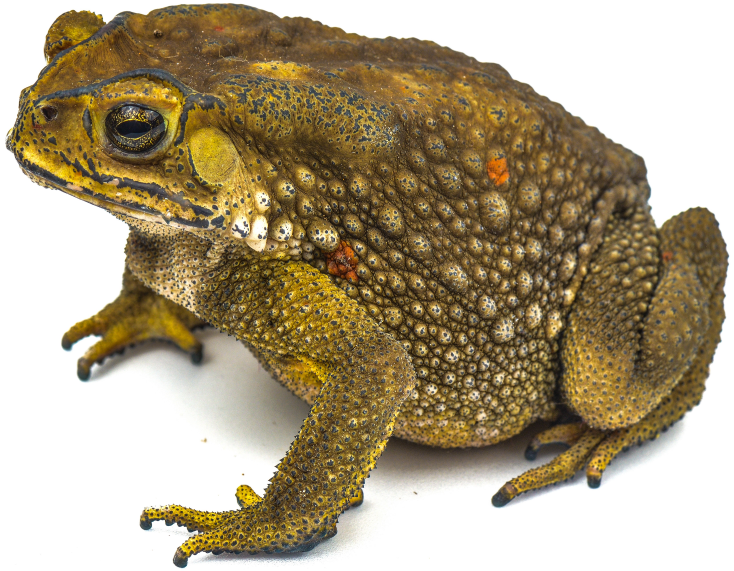

5

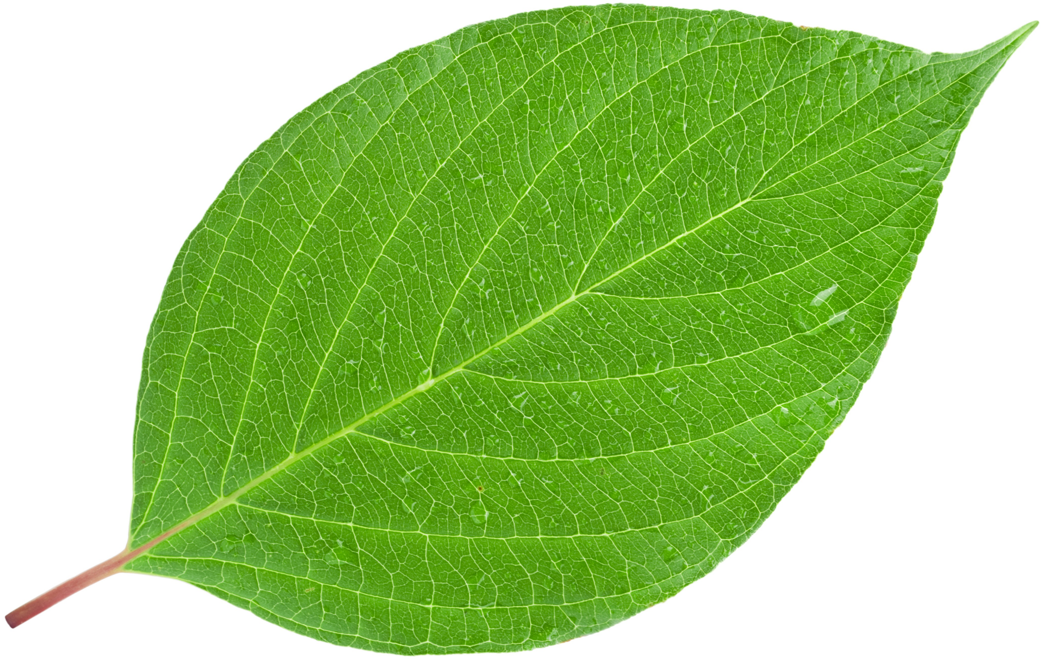

MEMÓRIA VERBAL

Memória Verbal

Memorizar e repetir o que é ditado:

1. Cinza é a cor do rato que entrou na casa.
2. O rato cinza entrou na casa amarela que tinha uma porta.
3. Sapato – andorinha – ameixa – luva
4. 4 – 0 – 1 – 9 – 7
5. Q – J – D – I – V – O

SEMÂNTICA

A figura/ palavra de cima combina com qual figura de baixo?

1. A figura de cima combina com qual figura de baixo?

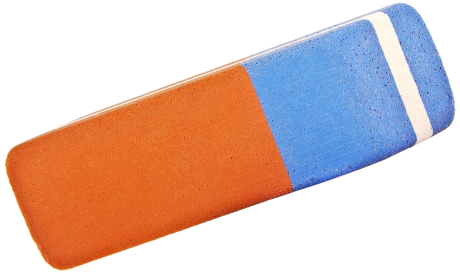

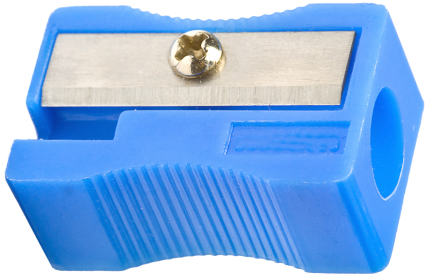

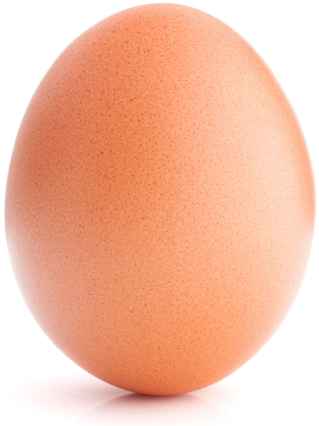

2. A palavra de cima combina com qual figura de baixo?

água

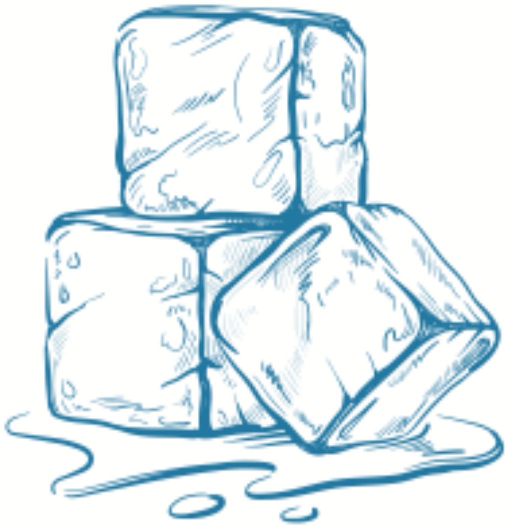

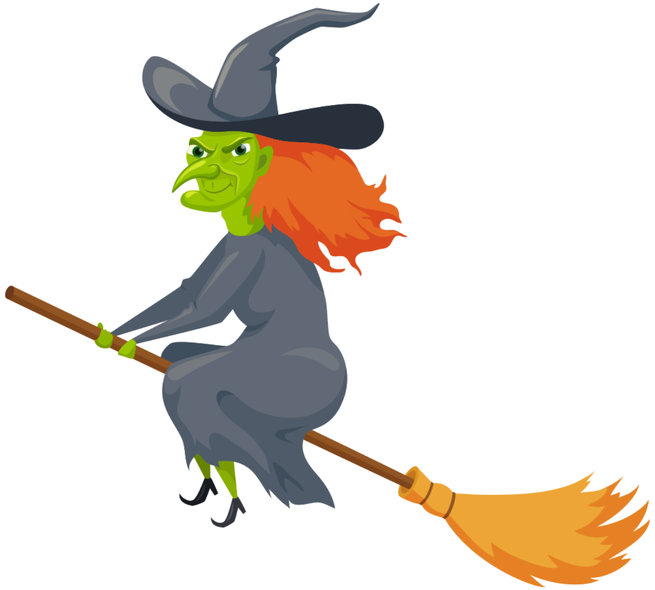

Semântica com dupla tarefa
(Tocar a orelha direita com a mão esquerda e vice-e-versa, alternadamente, enquanto responde o exercício)

Para que serve?

3. Para que serve?

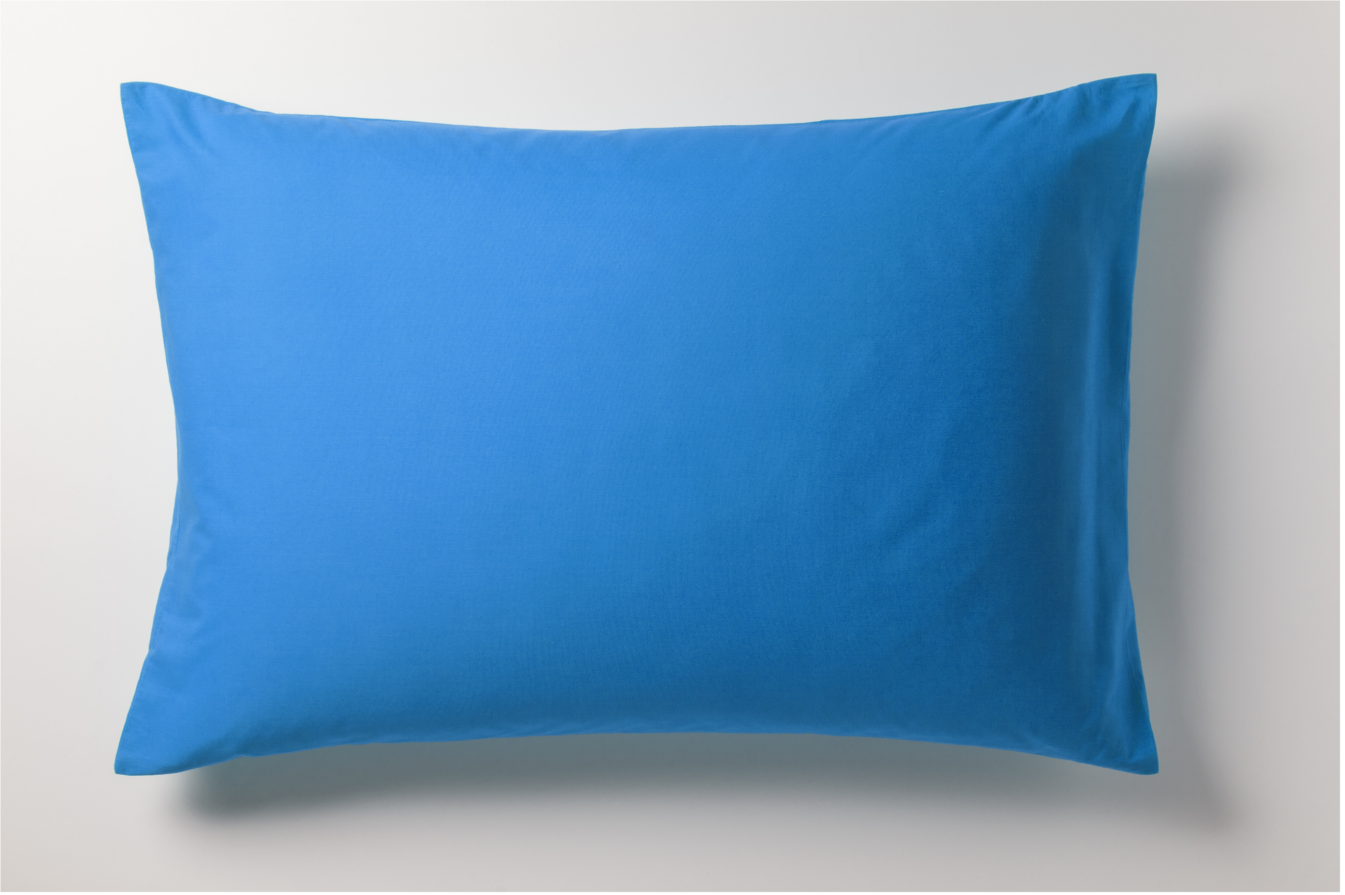

Semântica

De que material é feito?

4. De que material é feito?

chave

Semântica

Que forma tem?

5. Que forma tem?

canudo

CÁLCULO

CALCULE E DIGA O RESULTADO.

1. 7 + 4 =

2. 15 – 8 =

3. 23 X 2 =

Cálculo

4. Some APENAS os números não repetidos e subtraia 3 do resultado final.

1 – 7 – 3 – 2 – 1 – 8

5. Subtraia 3 do número 20 e siga subtraindo 3 dos resultados encontrados por mais 4 vezes seguidas.

GRAFIA

Grafia

Cópia de palavras:

1. Fantasia
2. Borracha

Cópia de frases:

3. Atualmente, cumpre seus compromissos de forma organizada.

DITADO DE PALAVRA

4. Salamandra

DITADO DE FRASE

5. A viagem aconteceu há muito tempo, mas deixou boas lembranças.

MEMÓRIA VISUAL

Memória Visual

Observe as figuras do quadro A e a seguir, diga quais imagens do quadro B apareceram no quadro A.

1. Quadro A

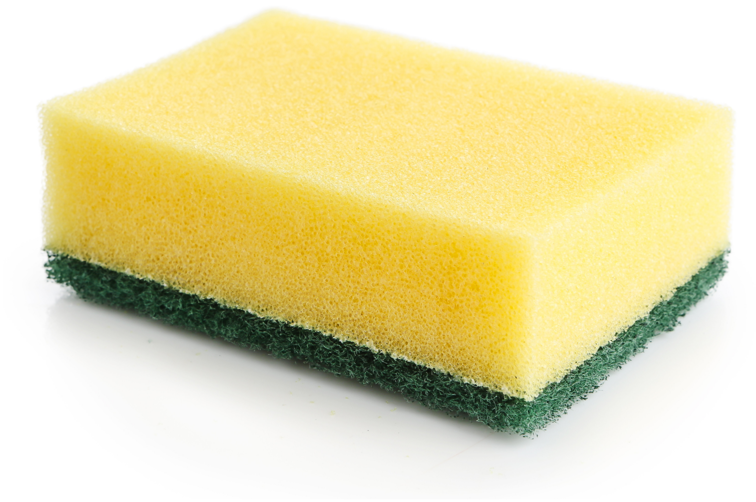

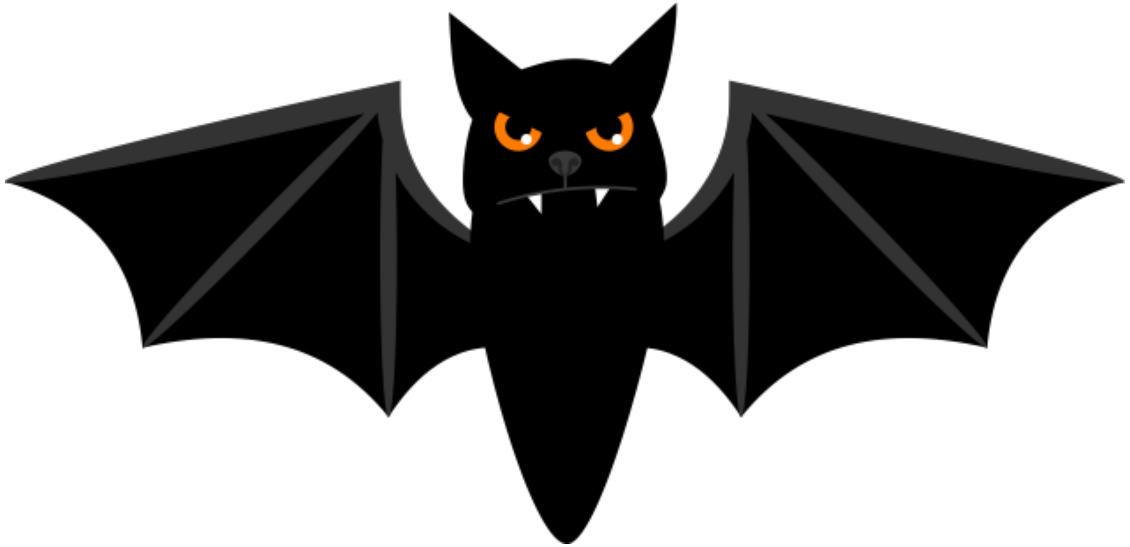

1. Quadro B

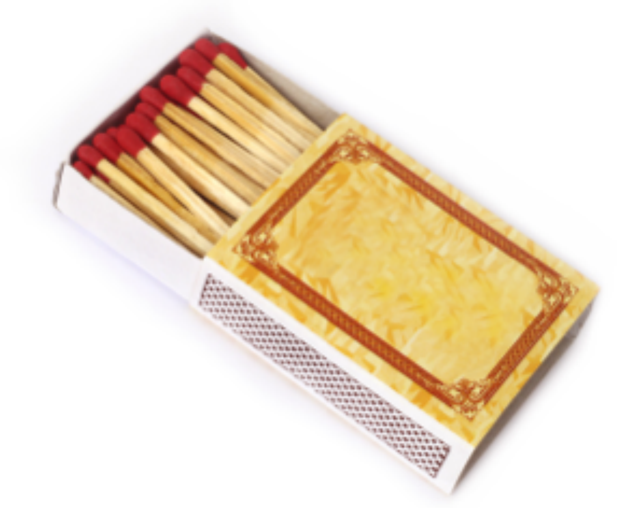

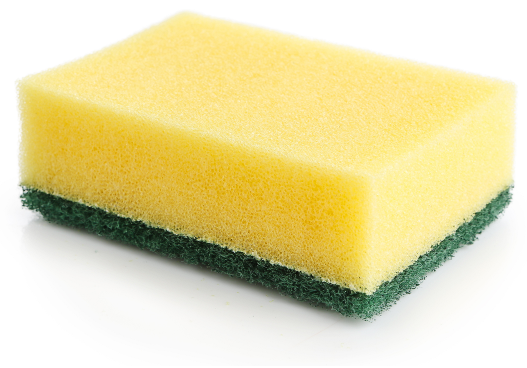

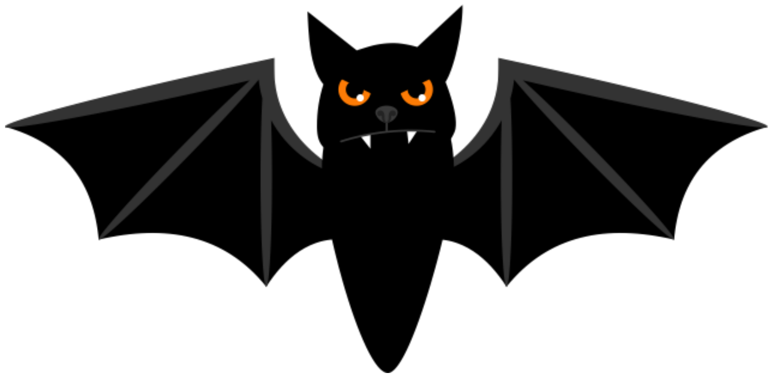

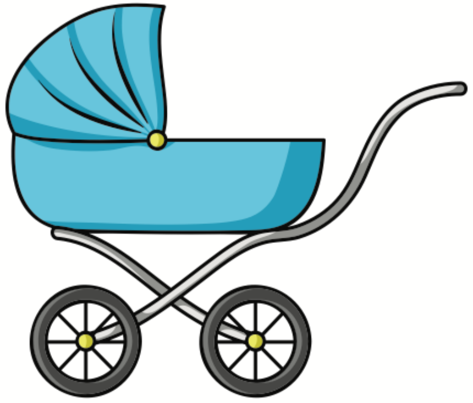

2. Quadro A

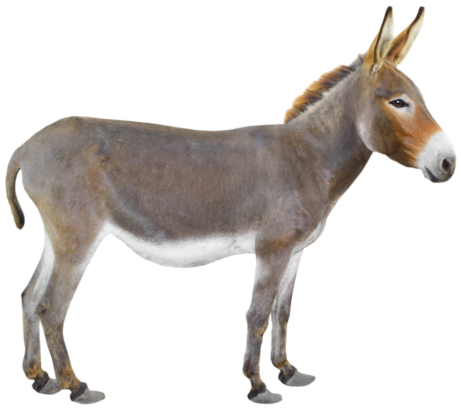

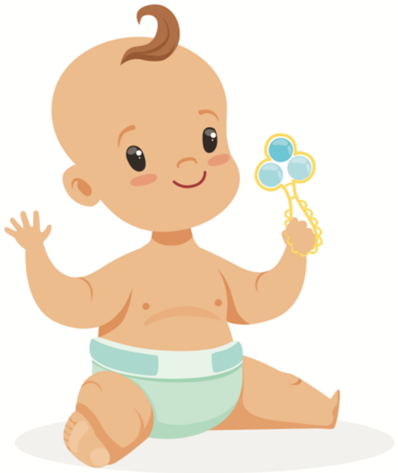

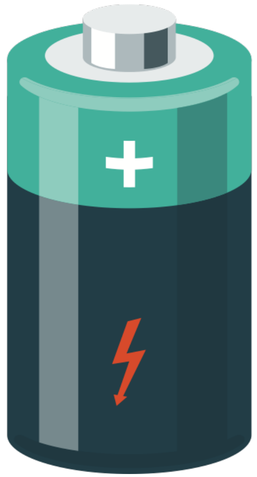

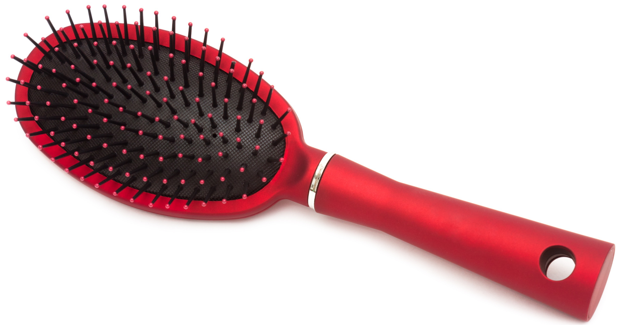

2. Quadro B

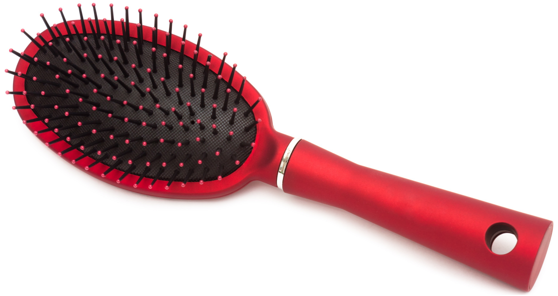
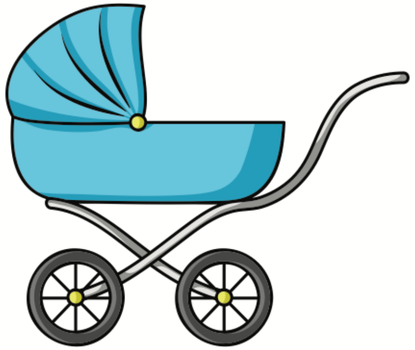
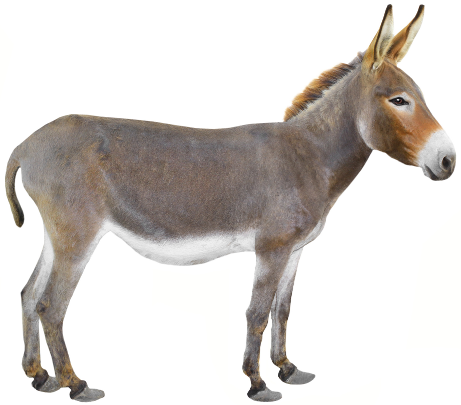
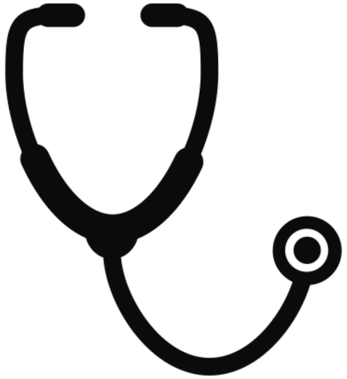
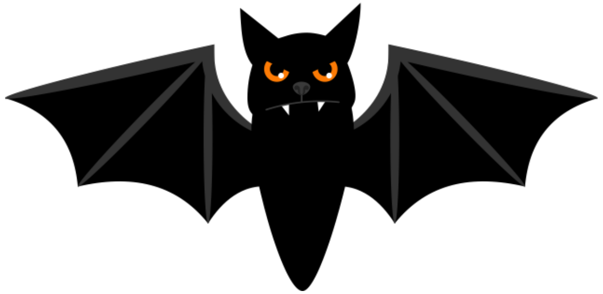
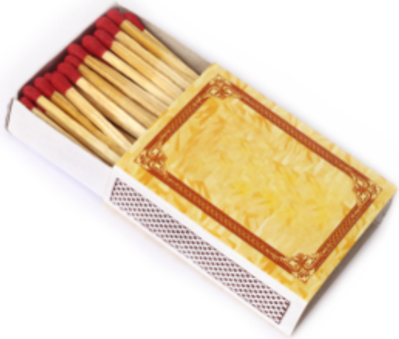
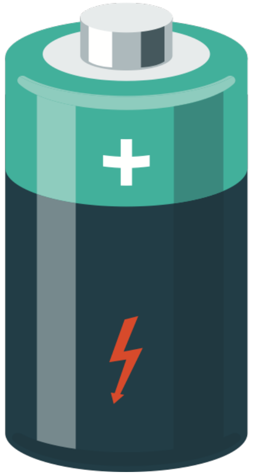
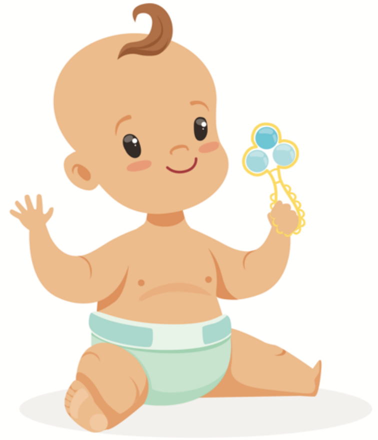

3. Quadro A

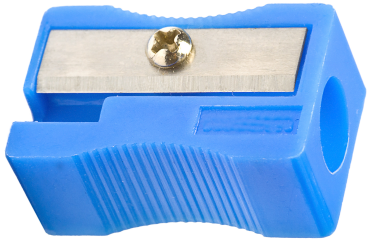

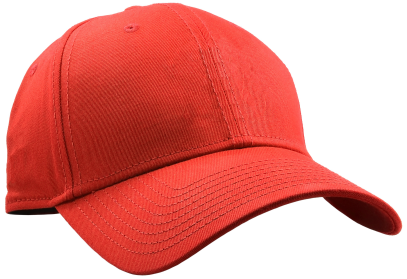

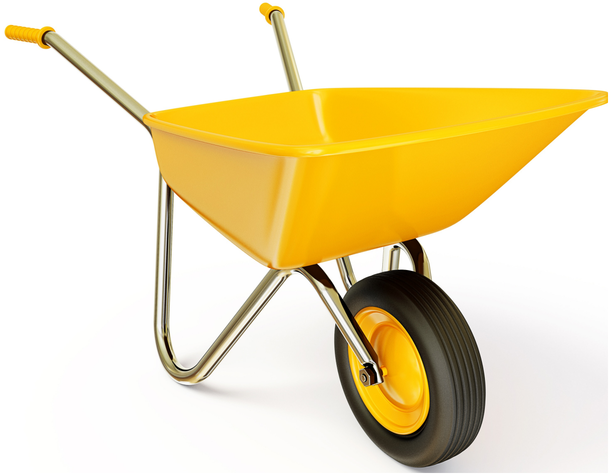

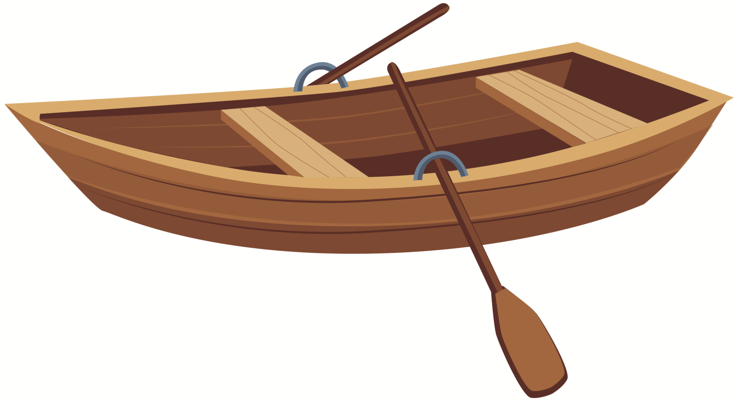

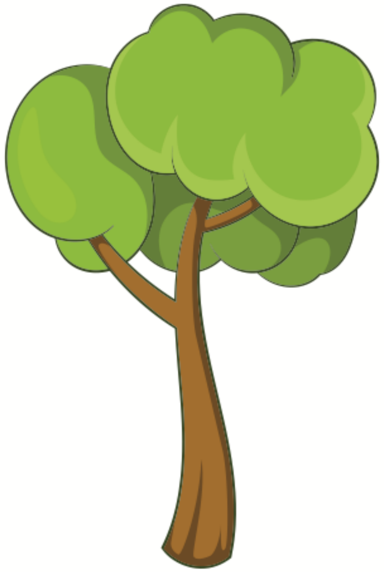

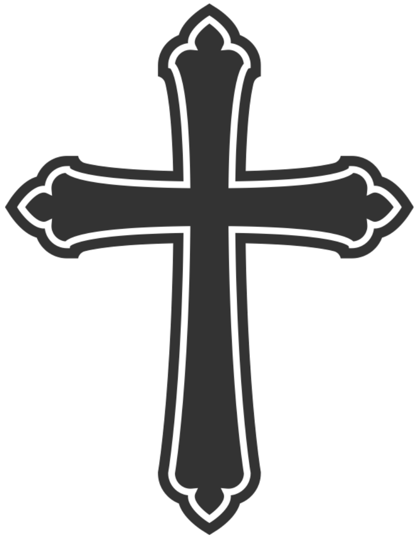

3. Quadro B

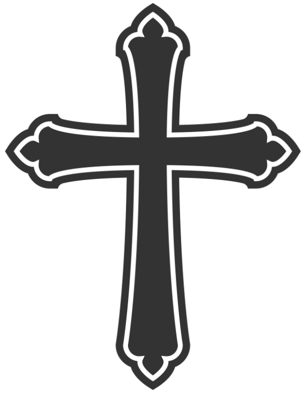
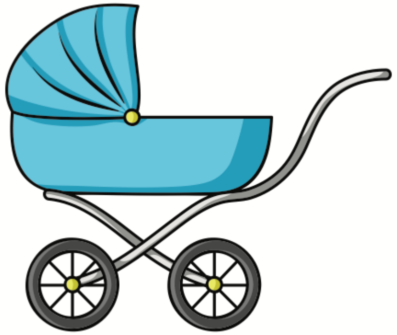
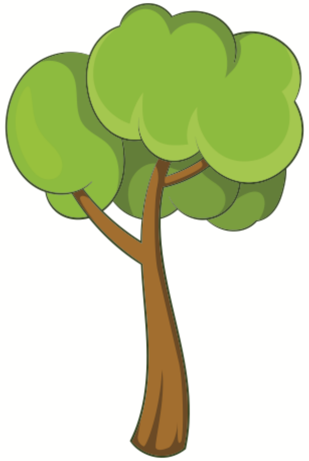
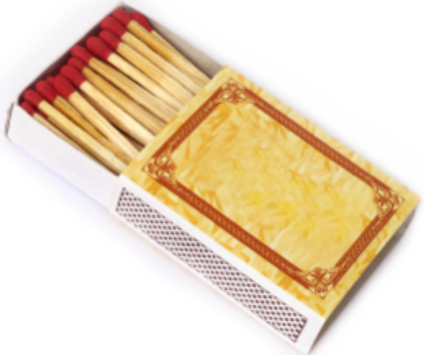
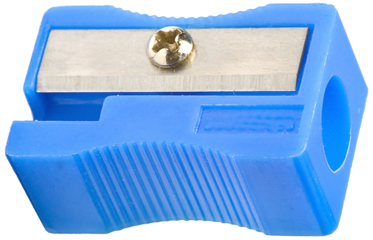
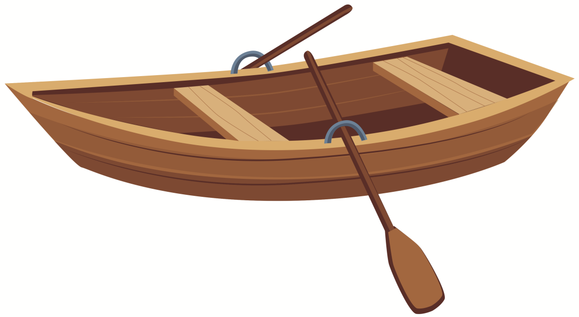
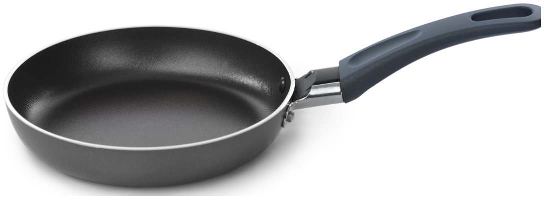
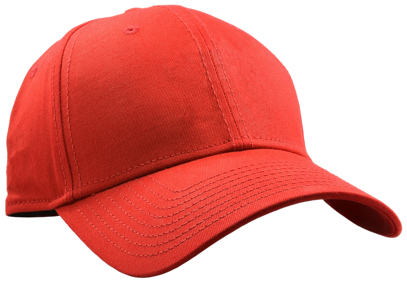
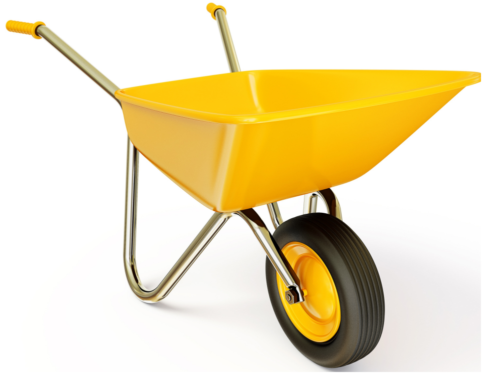

4. Quadro A

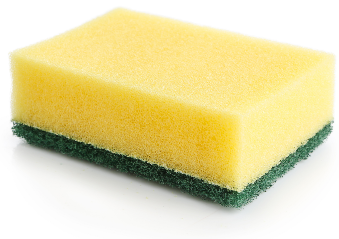
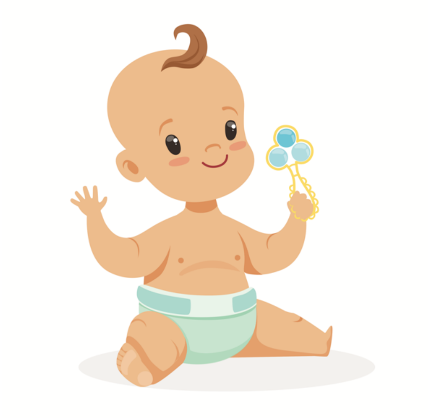
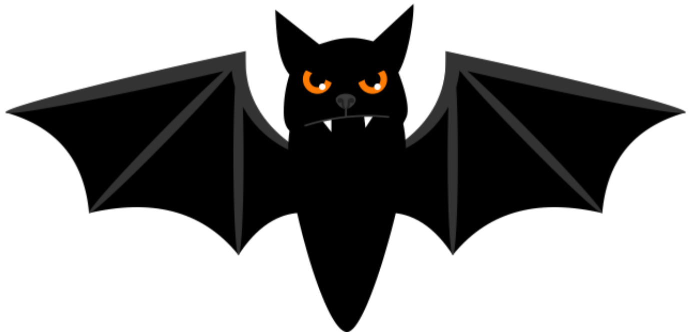
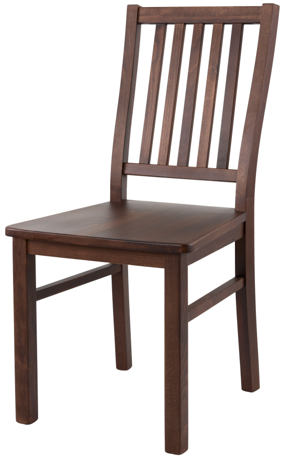
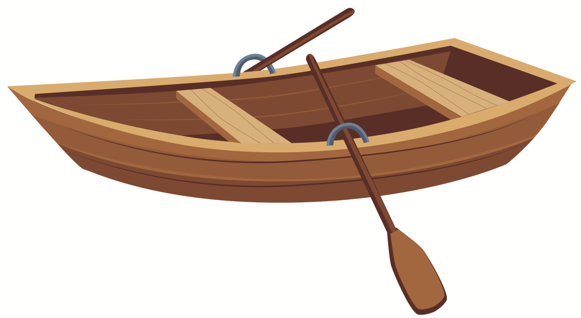
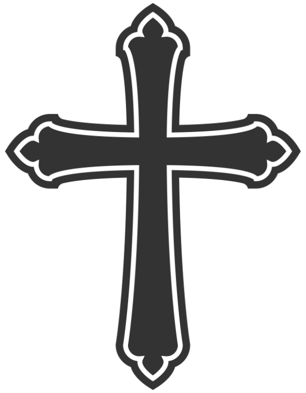
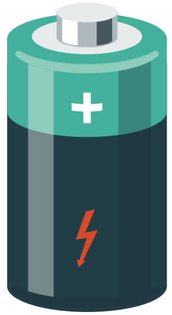
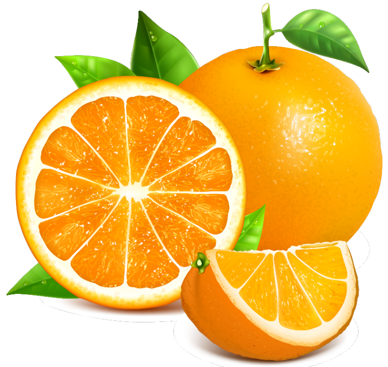

4. Quadro B

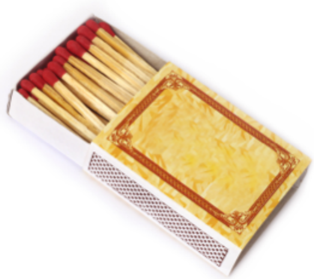
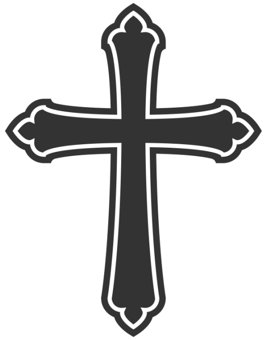
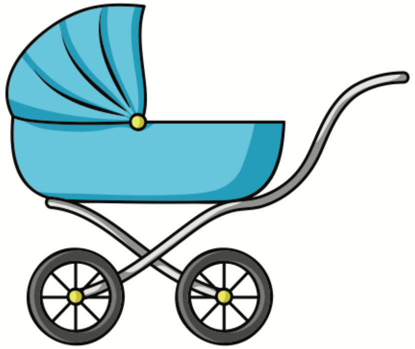
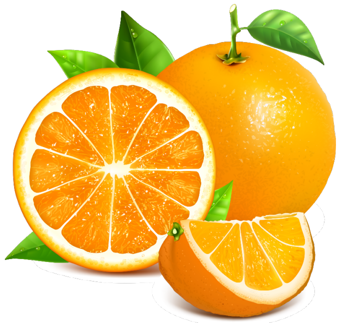
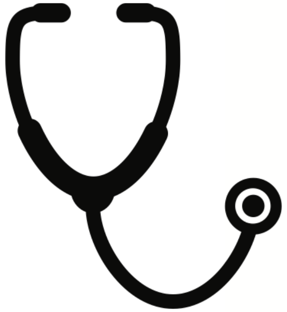
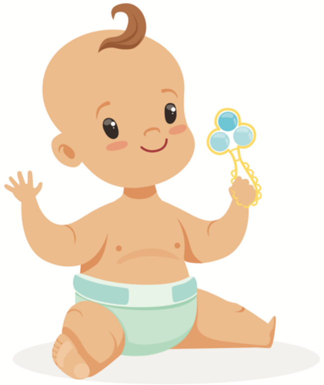

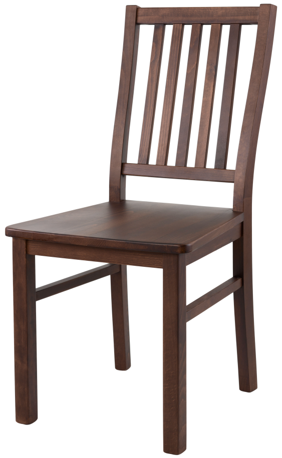
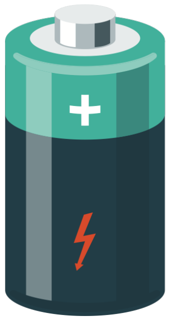
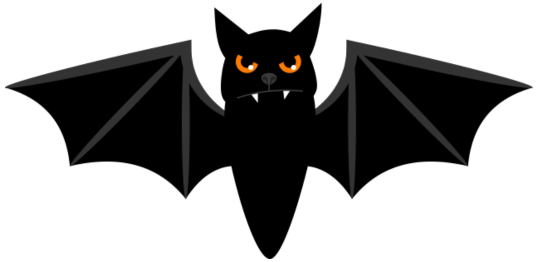
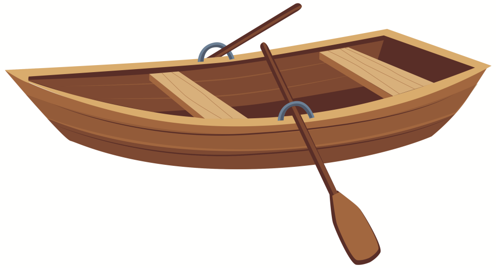
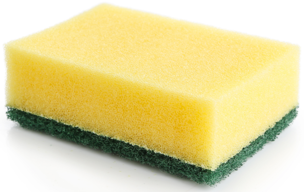

5. Quadro A

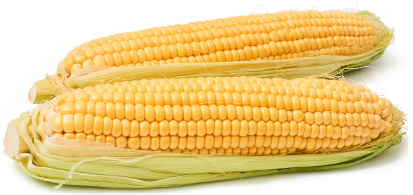
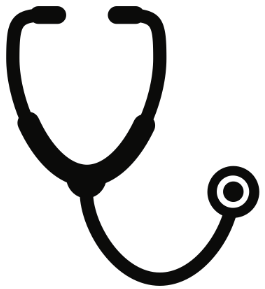
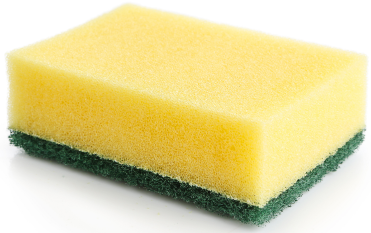
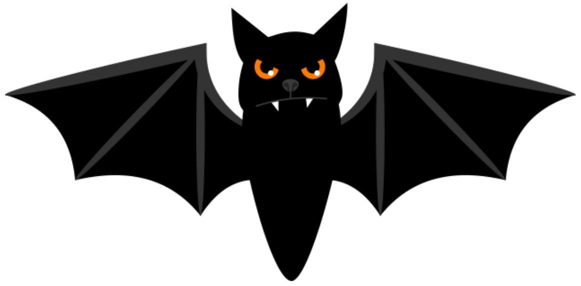
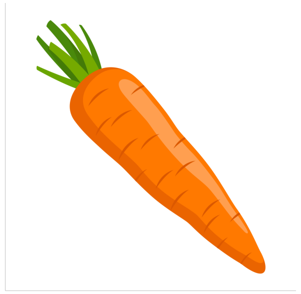
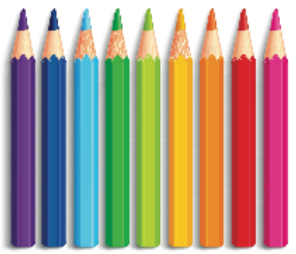
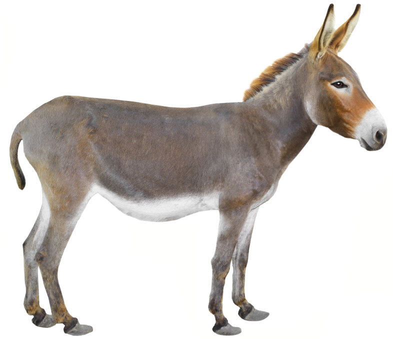
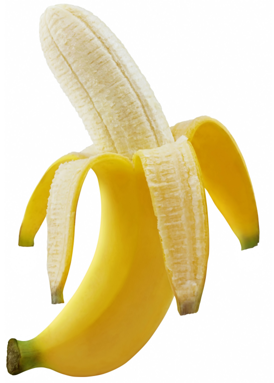
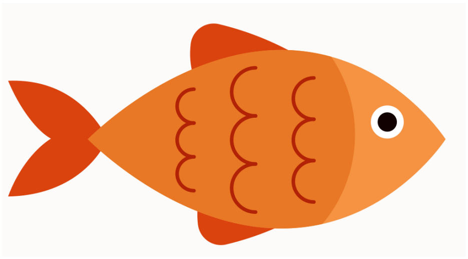
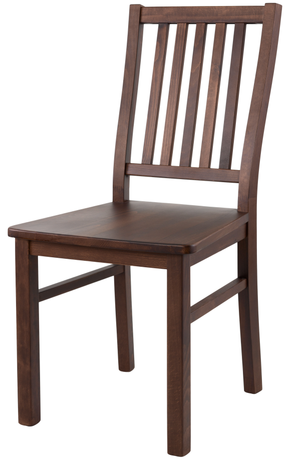

5. Quadro B

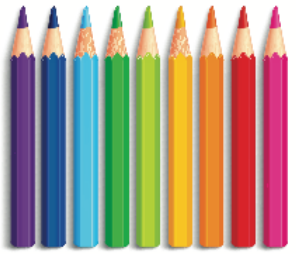
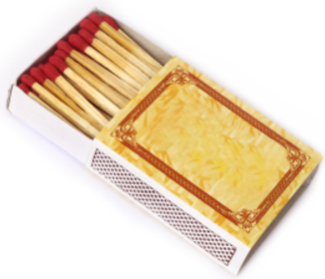
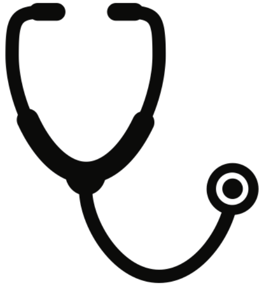
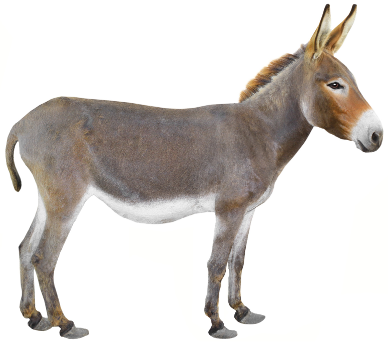
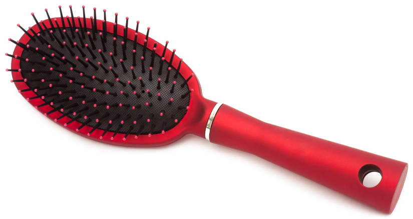
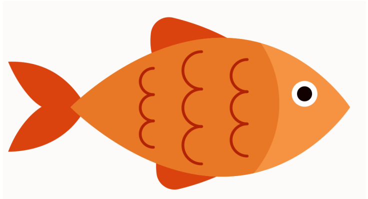
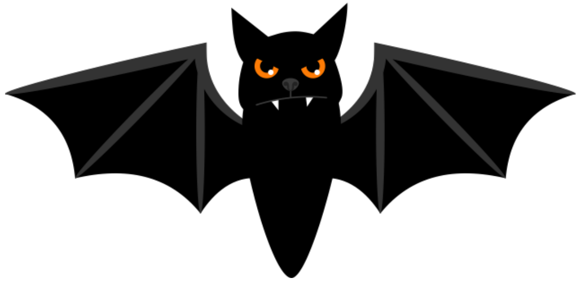
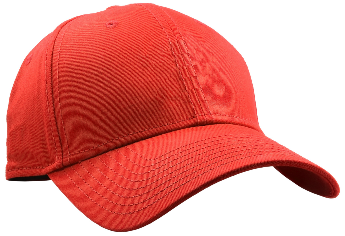
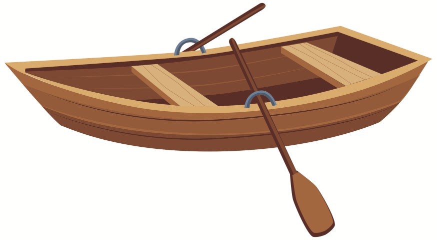
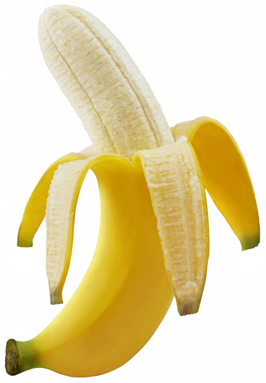
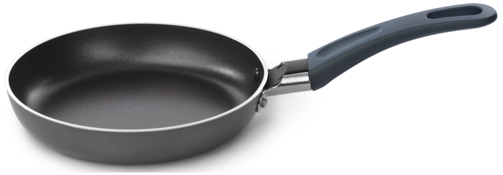
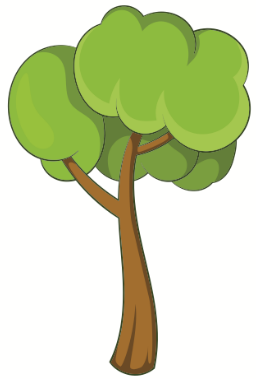
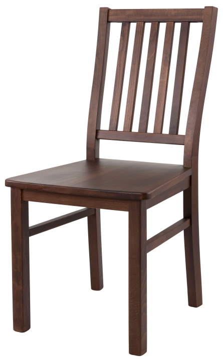
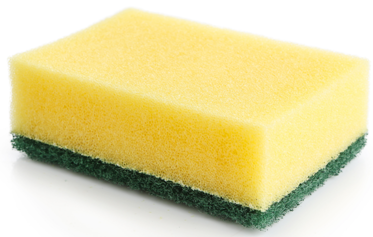
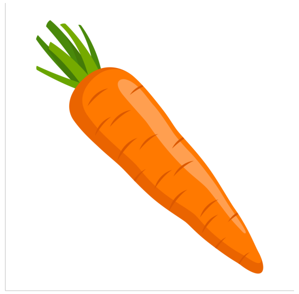
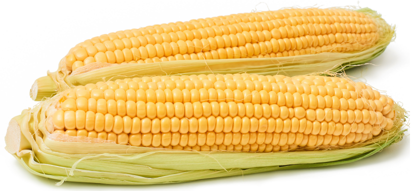

LEITURA E REPETIÇÃO

Leia e repita em voz alta as frases a seguir

1. Fala, arara loura. A arara loura falará.
2. A babá boba bebeu o leite do bebê.
3. Quem era Hera? Hera era a mulher de Zeus.
4. La vem o velho Félix com o fole velho nas costas.
5. Toco preto, porco fresco, corpo crespo.

SINTAXE

Sintaxe – Formação de palavras

Descobrir uma palavra:

1. LHA – FA
2. NE – CA – TA
3. VE – PE – EN – LO

Sintaxe – Formação de palavras

Descobrir a palavra:

4. A palavra LEAL, só **não** se encaixa em:

a) ( ) dis b) ( ) des c) ( ) dade

5. Local onde se compra LIVROS: ________________________________

DESENHO

Desenhe as formas a seguir, na folha de resposta.

1. Círculo
2. Triângulo
3. Cubo

Copie as formas a seguir, na folha de resposta.

4.

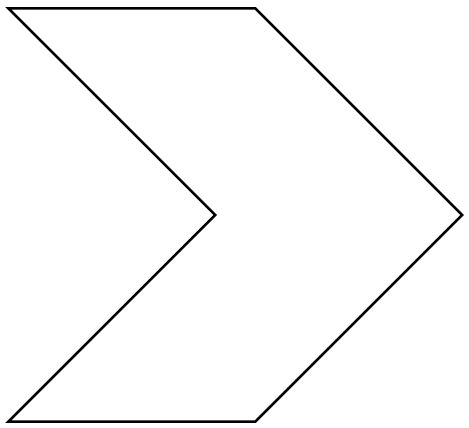

5.

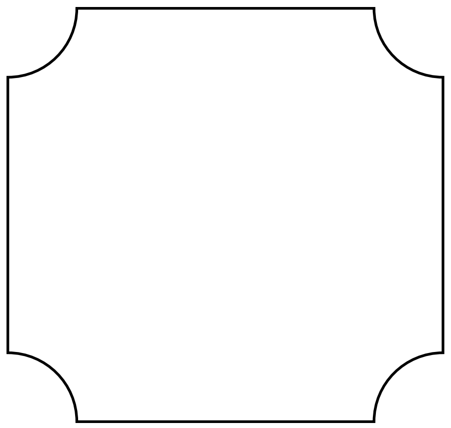

SOLETRAÇÃO

Soletrar na ordem direta

1. ARCO
2. BALEIA
3. SANDUÍCHE

Soletrar na ordem inversa

4. ADORAR
5. ESTREIA
