# Supplementary material for: Filling the gap: brief neuropsychological assessment protocol for glioma patients undergoing awake surgeries
Source: Front Psychol. 2024 Aug 9;15:1417947. doi: 10.3389/fpsyg.2024.1417947 (PMC11342098; doi:10.3389/fpsyg.2024.1417947)
Supplement: Supplementary file 4 [file Data_Sheet_4.PDF]

OMFTCT

Ohy-Maldaun Fast Track Cognitive Test

PREOPERATIVE (T1)

NAMING

Instruction: Say the name of the figure

1. **Instruction:** Say the name of this figure

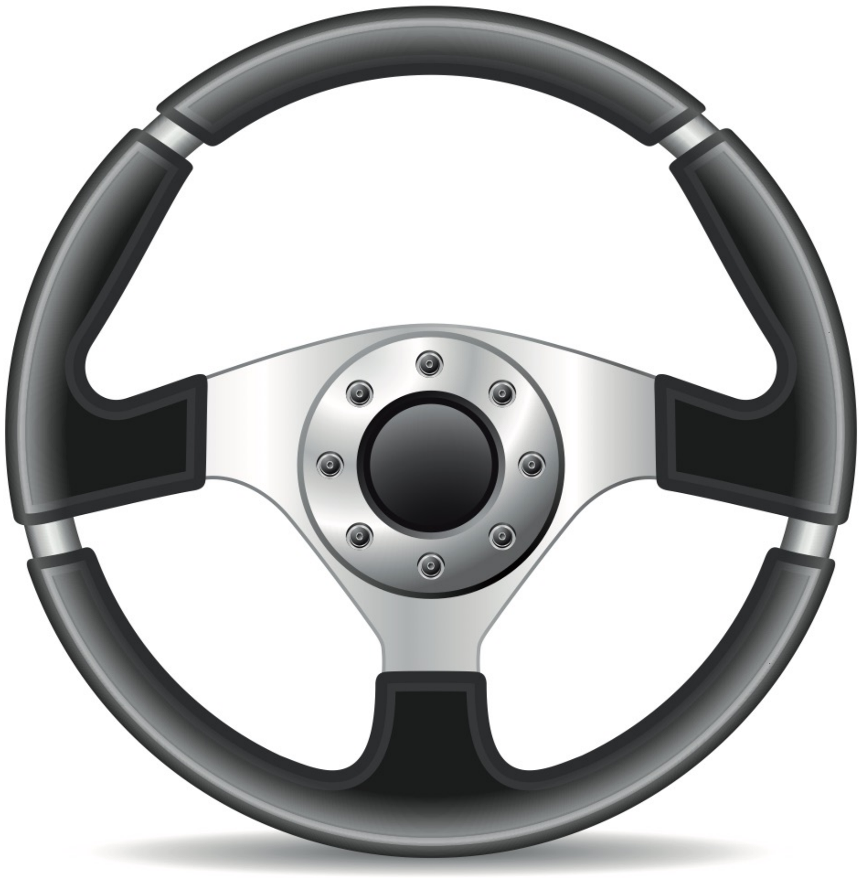

1. **Instruction:** Say the name of this figure

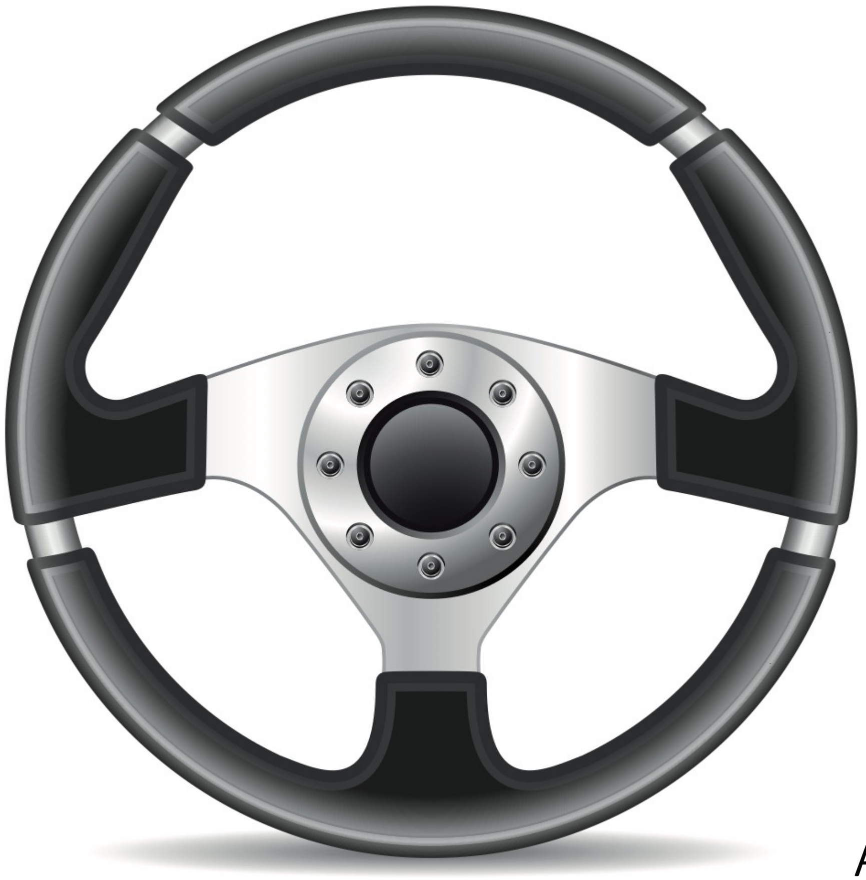

Answer: steering wheel

2. **Instruction:** Say the name of this figure

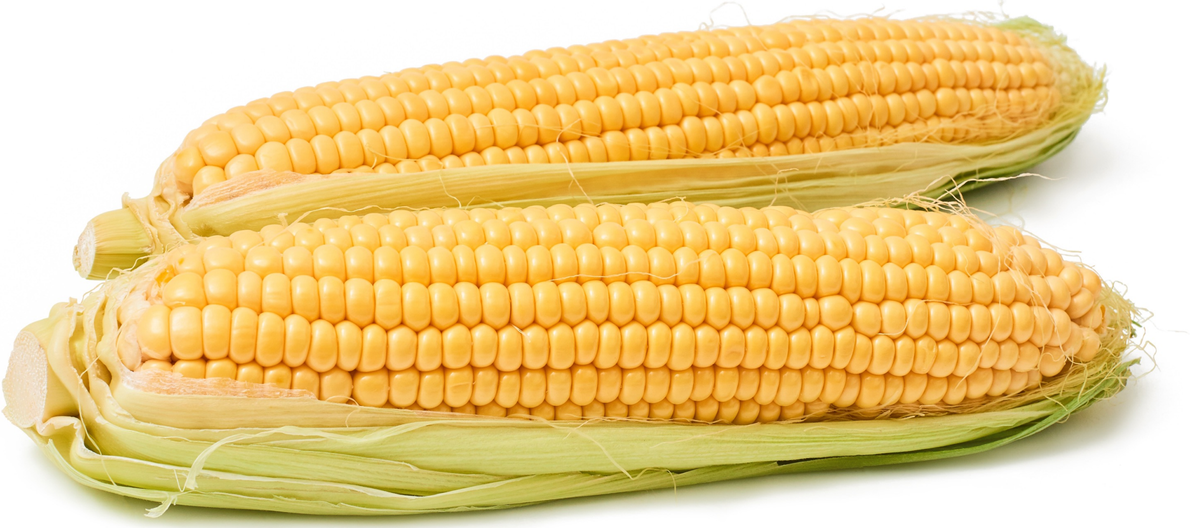

2. **Instruction:** Say the name of this figure

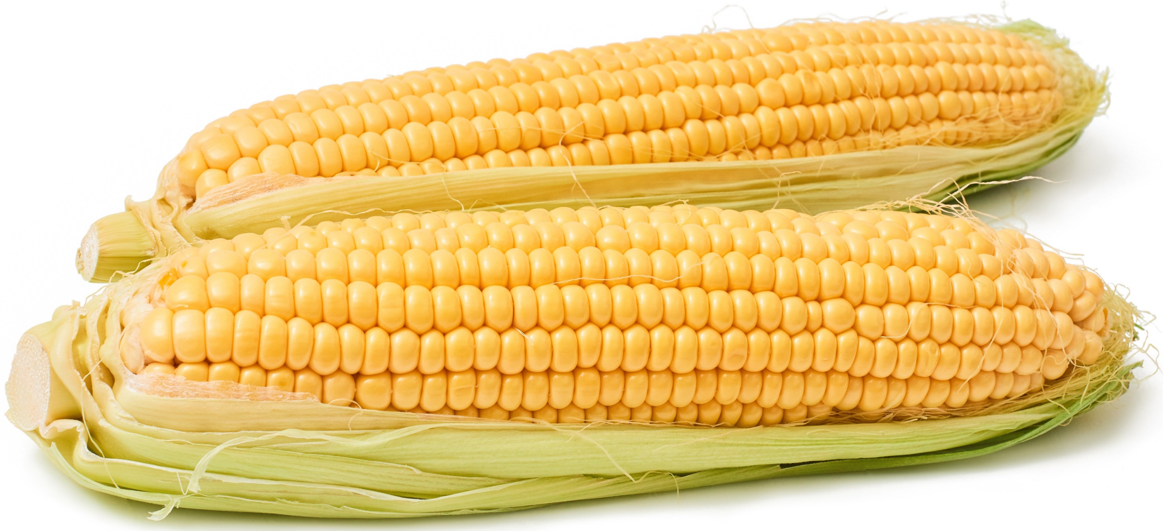

Answer: corn/corn cob

3. **Instruction:** Say the name of this figure

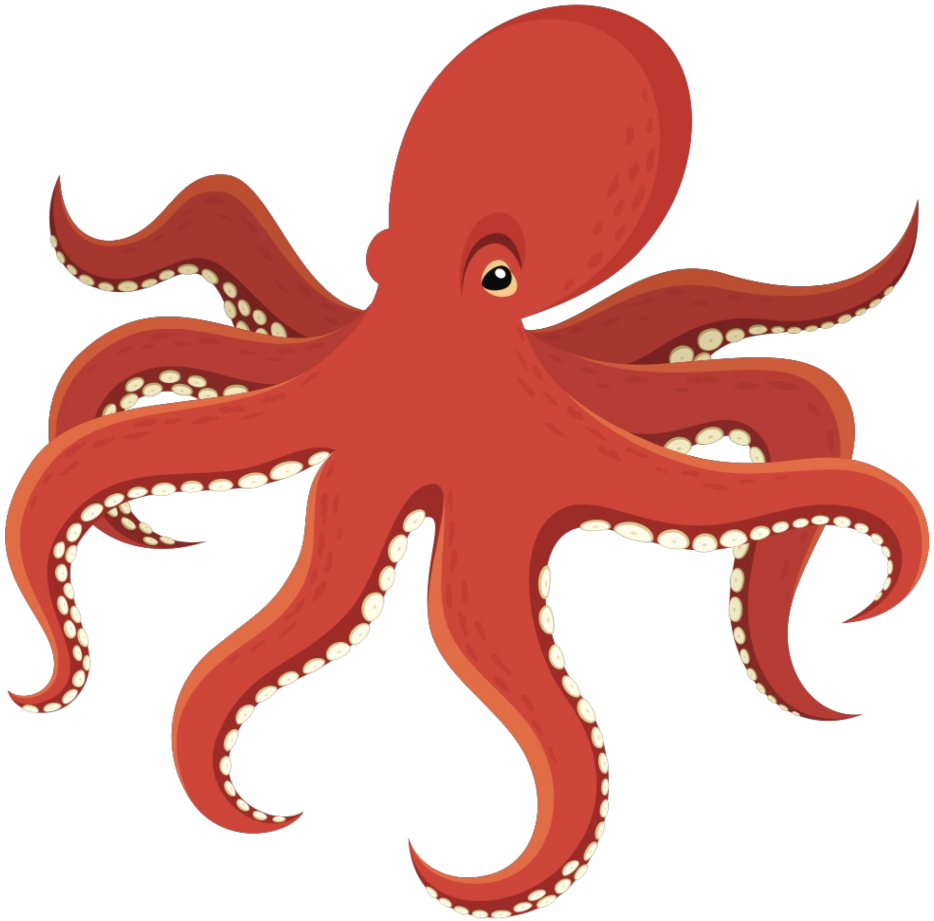

3. **Instruction:** Say the name of this figure

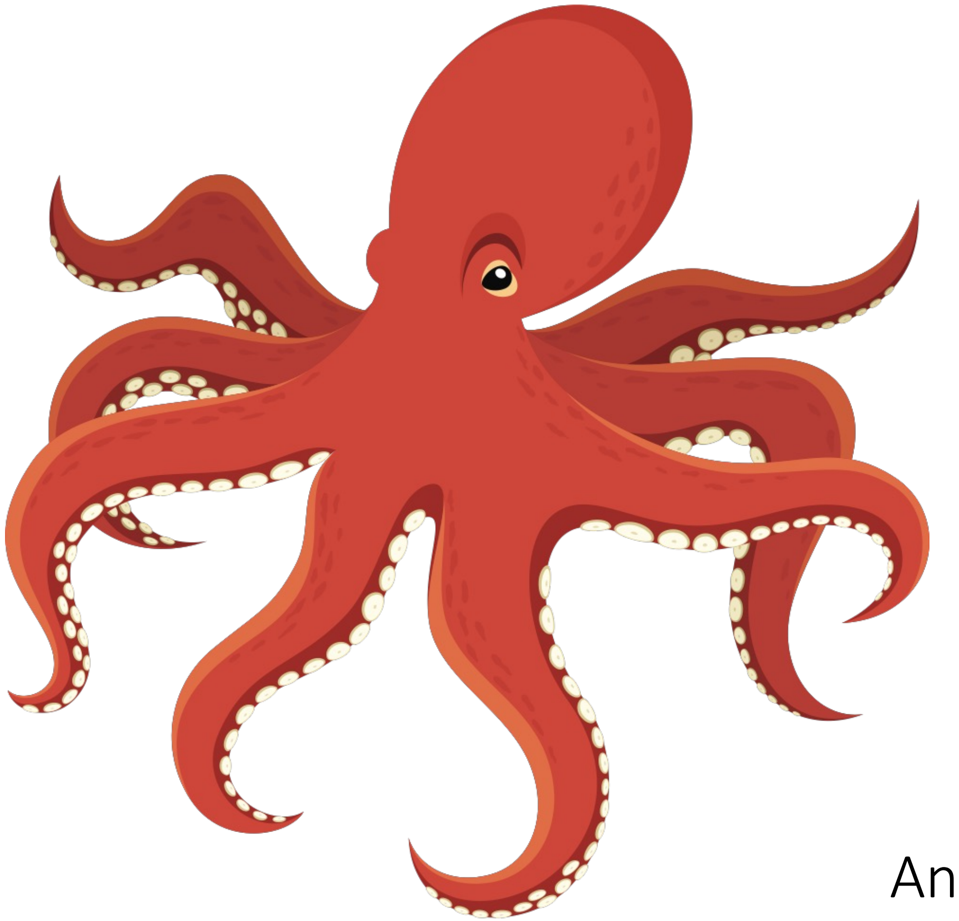

Answer: octopus

Dual task naming

Instruction: Open and close your hands alternautely while naming the figure

4. **Instruction:** Say the name of the figure while opening and closing your hands.

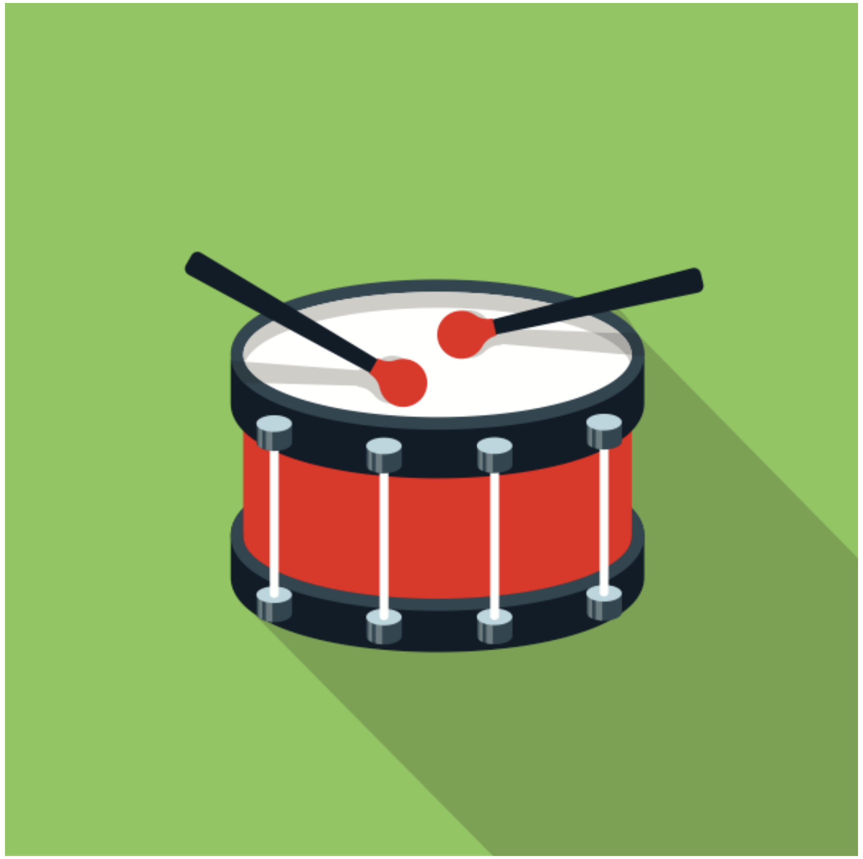

4. **Instruction:** Say the name of the figure while opening and closing your hands.

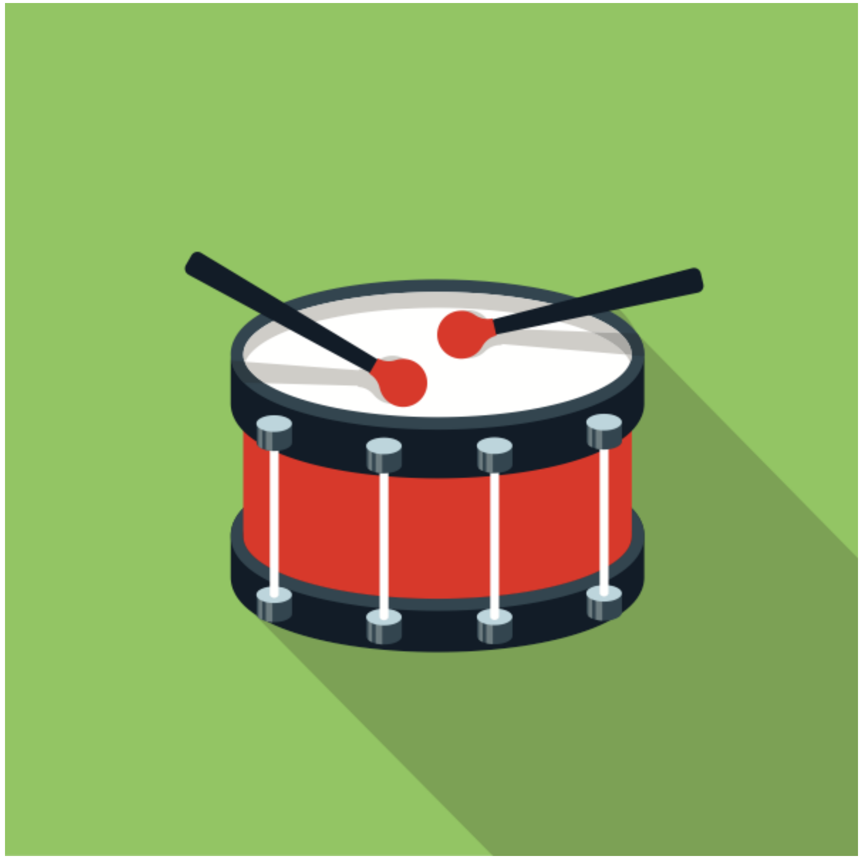

Answer: drum

5. **Instruction:** Say the name of the figure while opening and closing your hands.

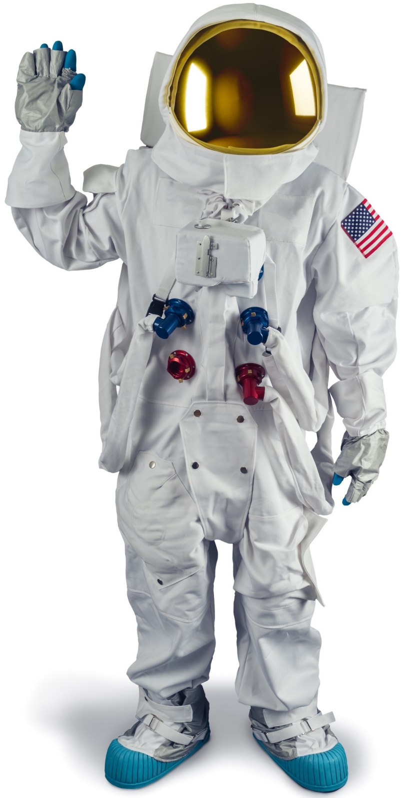

5. **Instruction:** Say the name of the figure while opening and closing your hands.

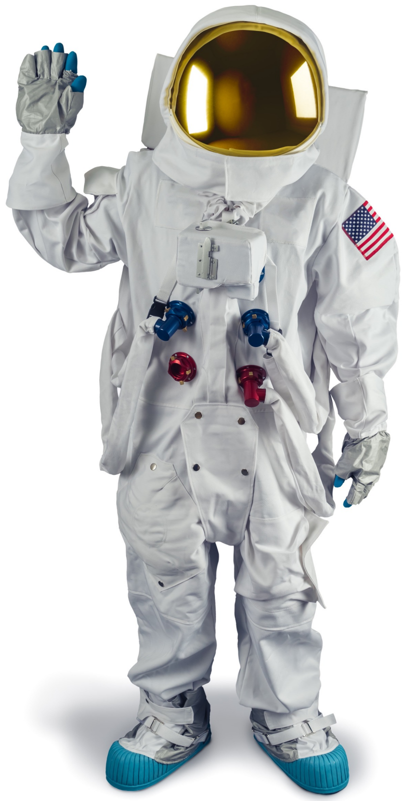

Answer: astronaut

VERBAL MEMORY

Instructions: Memorize and repeat what is said

VERBAL MEMORY

Instructions: Memorize and repeat what is said

1. Gray is the color of the mouse that entered the yellow house.
2. The gray mouse entered the yellow house that had a red door in the laundry room.
3. Shoes – swallow – plum – glove – branch
4. 6 – 0 – 9 – 0 – 3 – 5 – 2
5. Q – J – D – I – V – O – P – W – L

SEMANTIC

Instruction: Say which figure at the bottom relates to the main figure at the top.

1. **Instructions:** Say which figure at the bottom relates to the main figure at the top

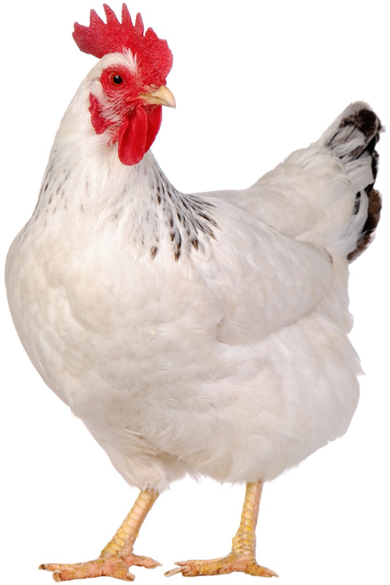

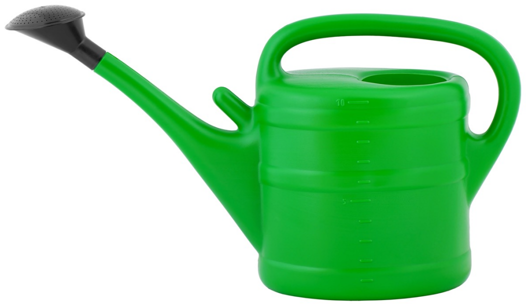

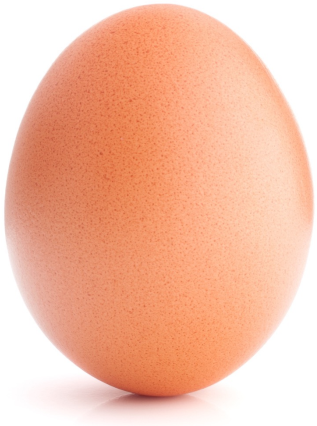

1. **Instructions:** Say which figure at the bottom relates to the main figure at the top

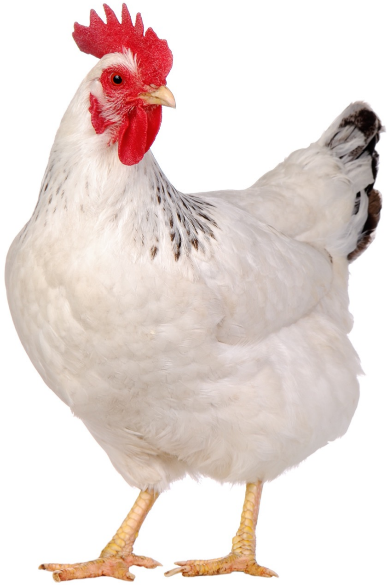

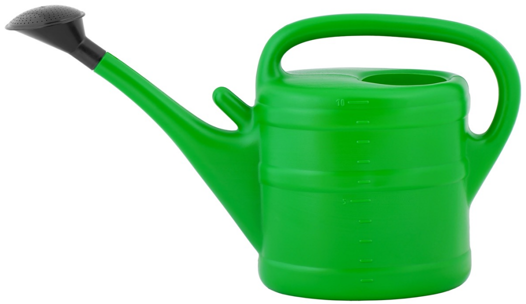

Answer: egg

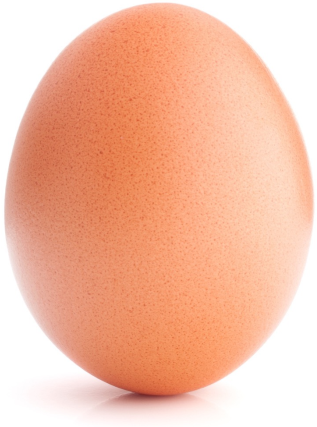

2. **Instructions:** Say which figure at the bottom relates to the main figure at the top.

fireman

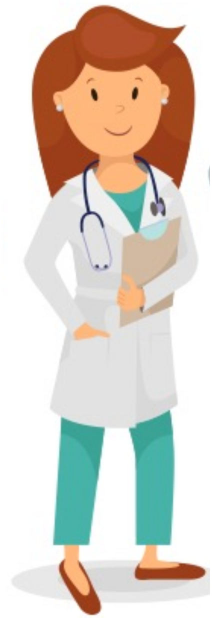

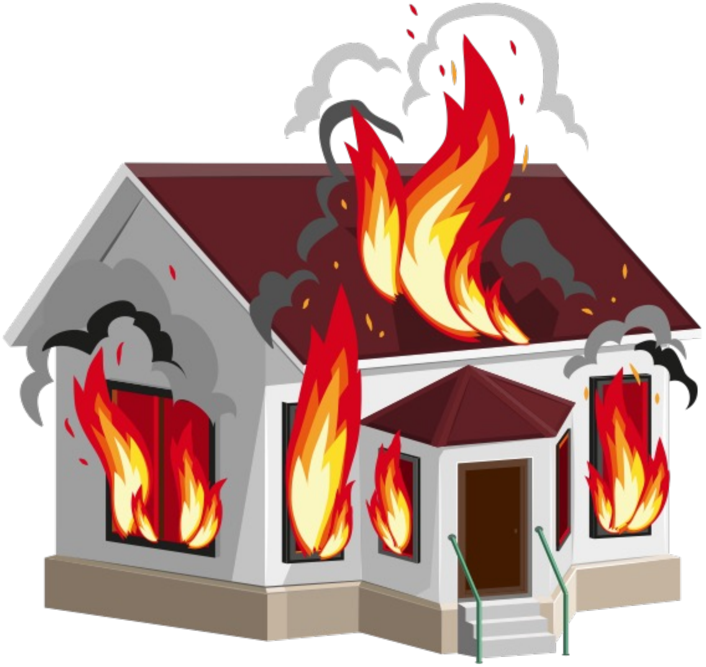

2. **Instructions:** Say which figure at the bottom relates to the main figure at the top.

fireman

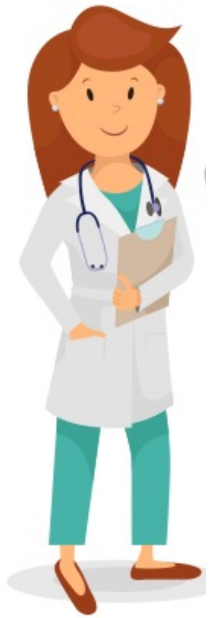

Answer: house on fire

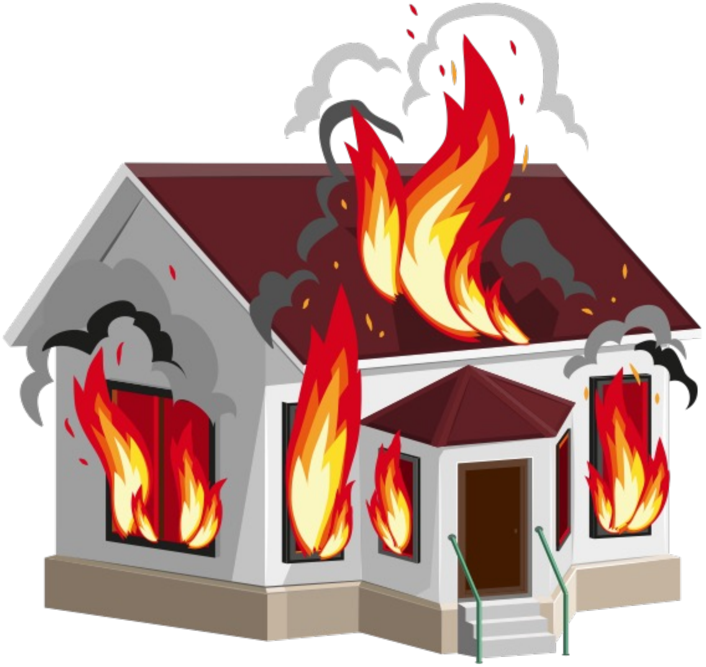

Dual task semantics

Instruction: Touch your right ear with your left hand alternately while answering the task

3. **Instructions:** Touch your right ear with your left hand alternately while answering the question

What is it for?

NAIL

3. **Instructions:** Touch your right ear with your left hand alternately while answering the question

What is it for?

NAIL

Answer: to pin/ to nail

4. **Instruction:** Touch your right ear with your left hand alternately while answering the question

What material is it made of?

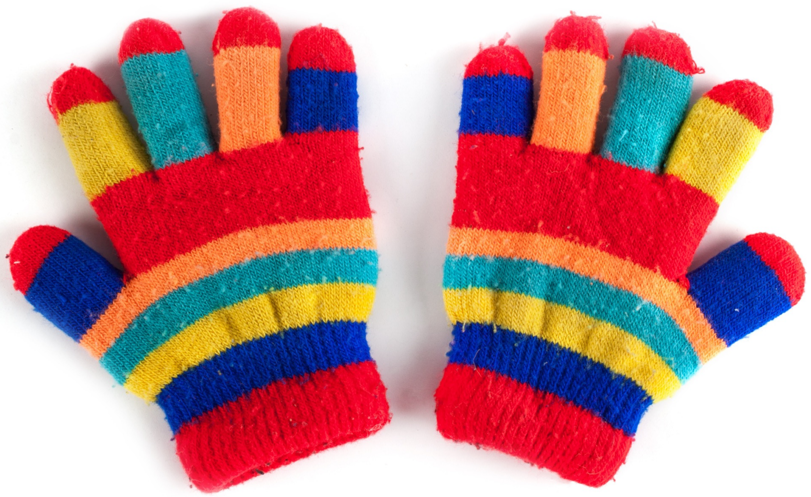

4. **Instruction:** Touch your right ear with your left hand alternately while answering the question

What material is it made of?

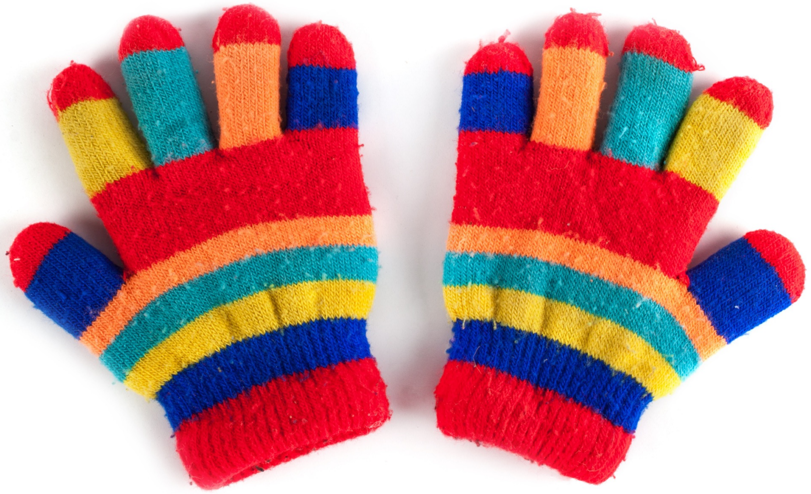

Answer: fleece/wool

5. **Instruction:** Touch your right ear with your left hand alternately while answering the question

What shape does it have?

PEN

5. **Instruction:** Touch your right ear with your left hand alternately while answering the question

What shape does it have?

PEN

Answer: cilinder

CALCULATION

Instruction: Calculate and state the result

Instruction: Calculate and state the result

1. 5 + 6 =

2. 12 – 7 =

3. 25 x 6 =

Instruction: Calculate and state the result

1. 5 + 6 = 11

2. 12 – 7 = 5

3. 25 x 6 = 150

4. Instruction: From the following sequence of numbers, identigy the three numbers that add up to 13.

5 – 8 – 3 – 10 – 2 – 9

5. Instructions: How many quarters are needed to obtain $4.00?

4. Instruction: From the following sequence of numbers, identigy the three numbers that add up to 13.

5 – 8 – 3 – 10 – 2 – 9

Answer: 2, 3, 8

5. Instruction: How many quarters are needed to obtain $4.00?

Answer: 16

WRITING

Instruction: Copy and transcribe the words and sentences

Instruction: Copy the words:

1. Analogy
2. Career

Instruction: Copy the sentence:

3. He carried a ton of oranges in his large bag.

Instruction: Write the dictated word

4. Maintenance

Instruction: Write the dictated sentence

5. There was no alternative but to give it as a gift.

VISUAL MEMORY

Instruction: Examine the pictures in box A and then identify which images from box B were present in box A.

1. **Instruction:** Memorize the pictures in box A

BOX A

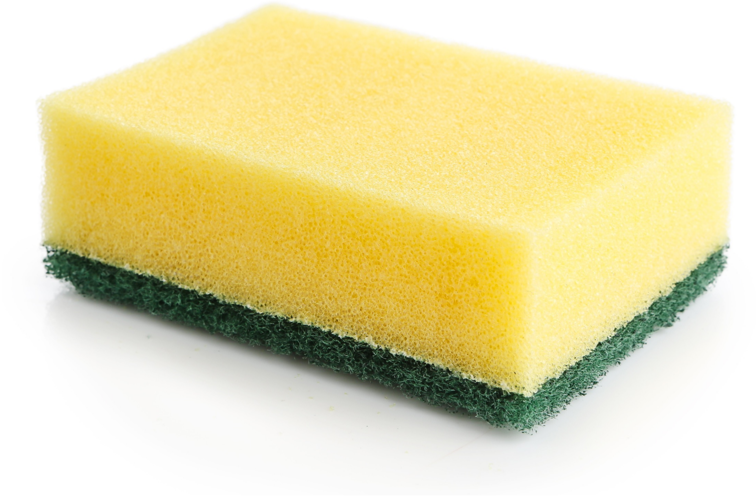

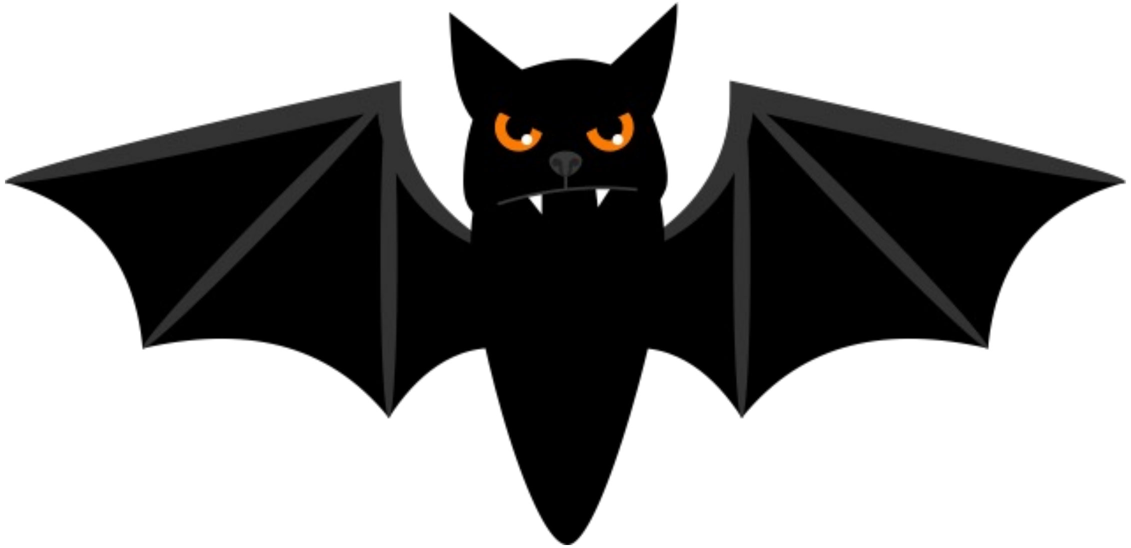

1. **Instruction:** Identify which pictures you recalled from box A and which are found in box B.

BOX B

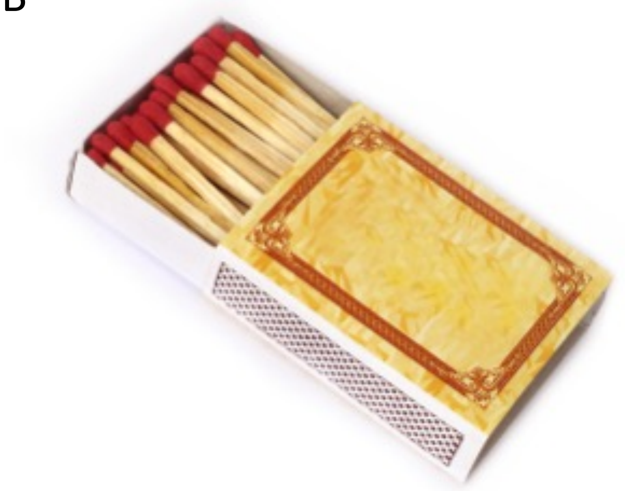

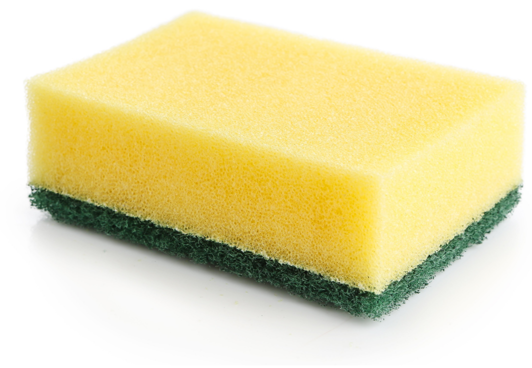

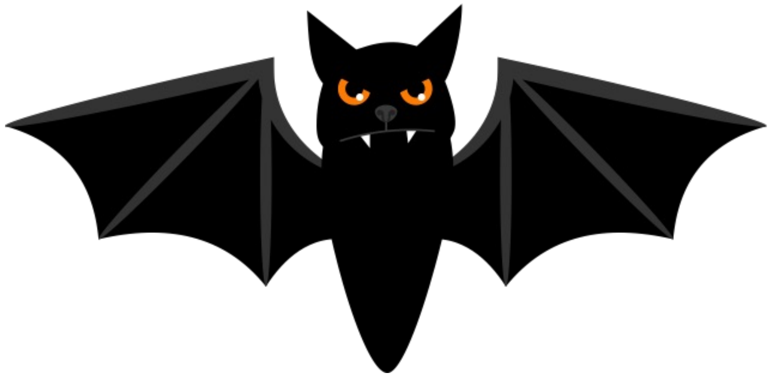

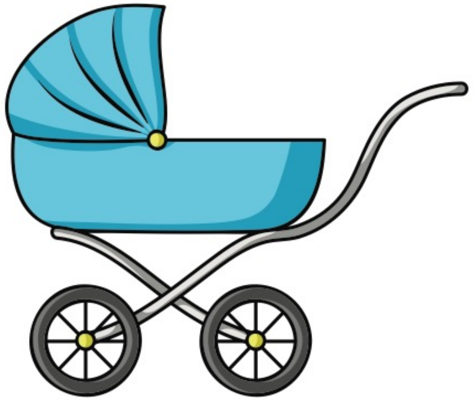

1. **Instruction:** Identify which pictures you recalled from box A and which are found in box B.

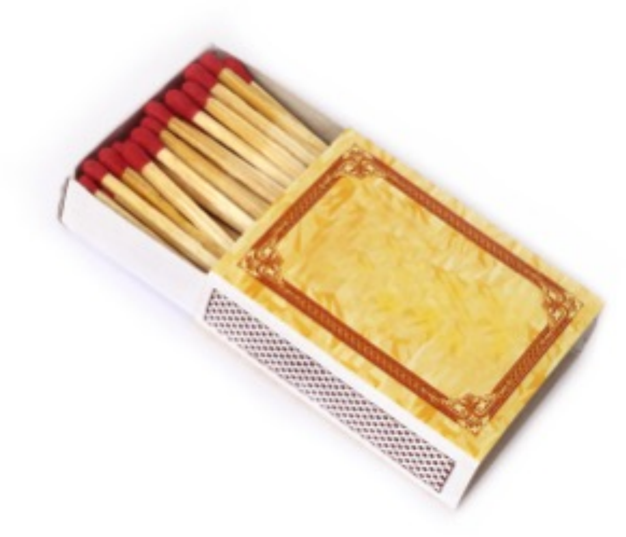

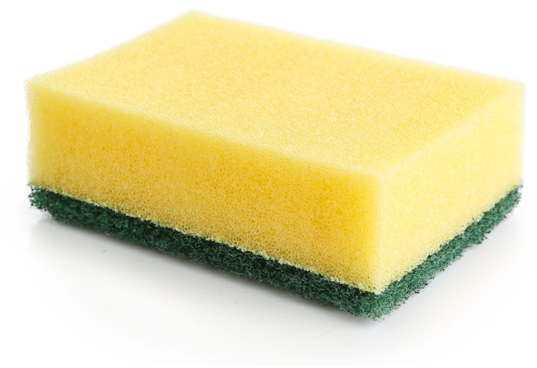

Answer: bat and sponge

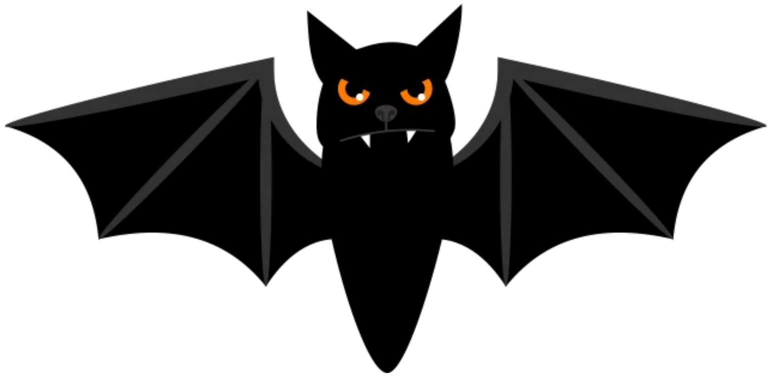

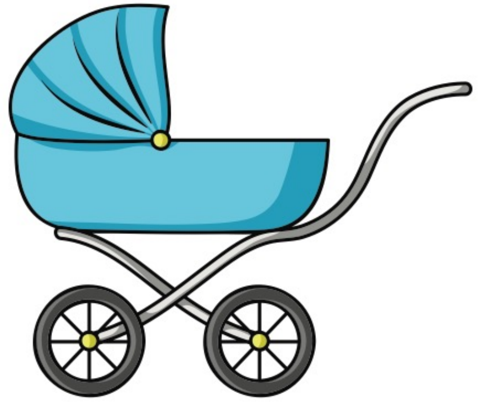

2. **Instruction:** Memorize the pictures in box A

BOX A

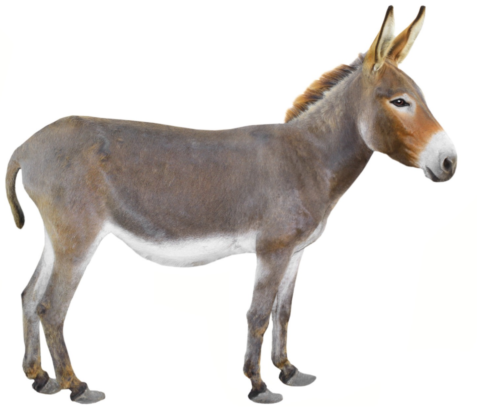

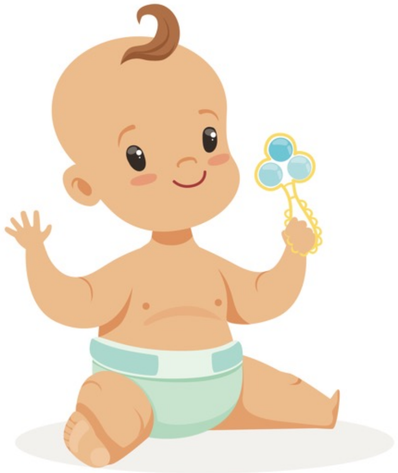

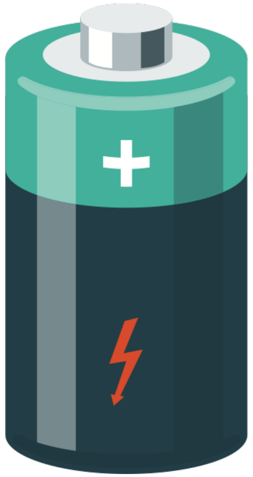

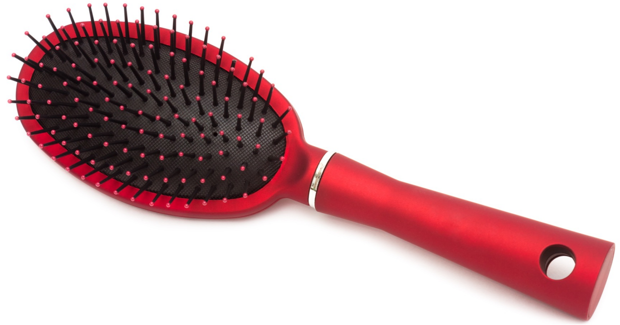

2. **Instruction:** Identify which pictures you recalled from box A and which are found in box B.

BOX B

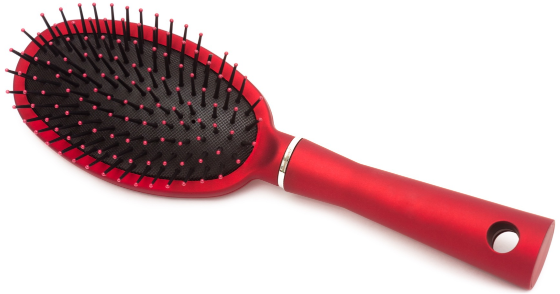
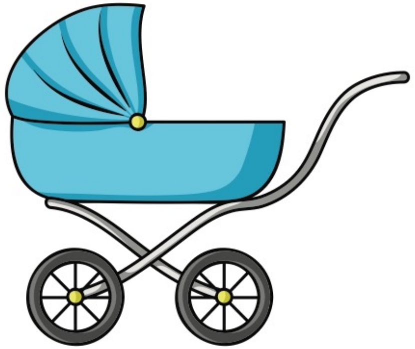
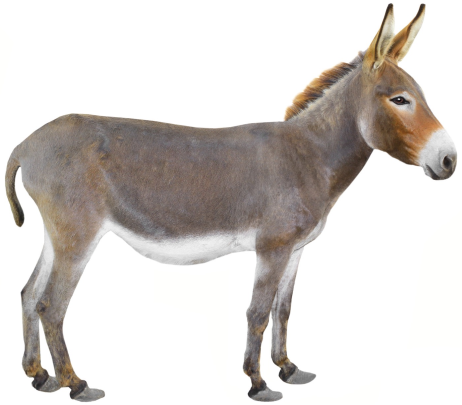
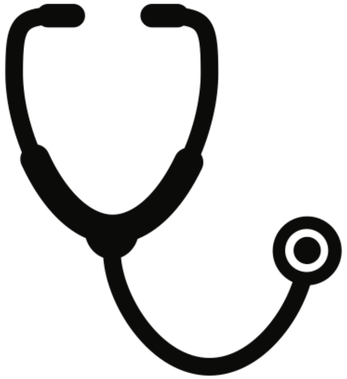
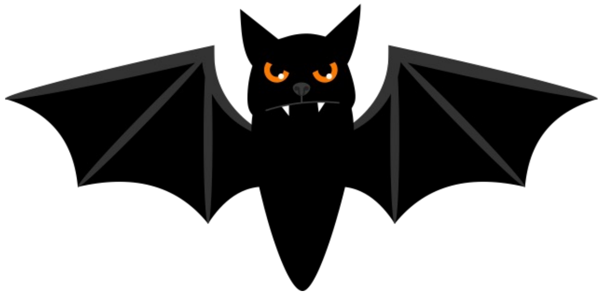
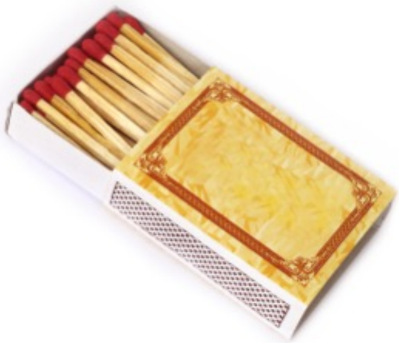
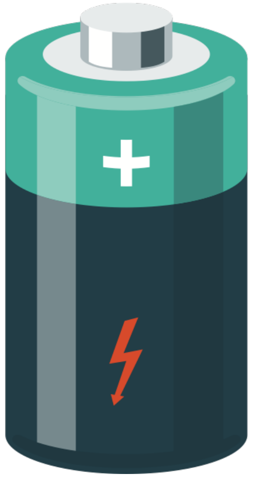
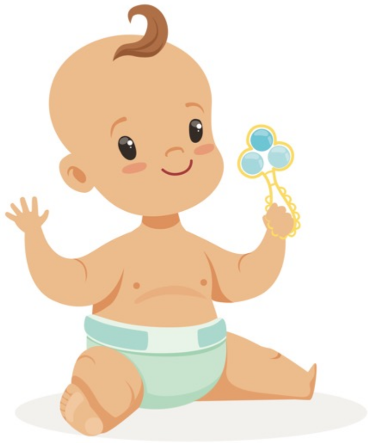

2. **Instruction:** Identify which pictures you recalled from box A and which are found in box B.

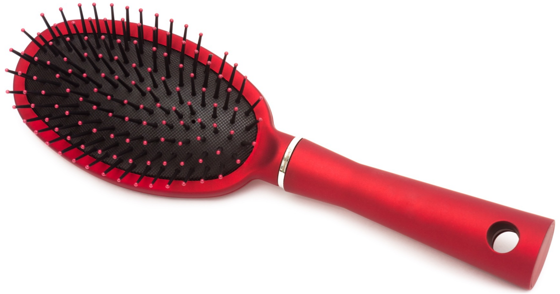
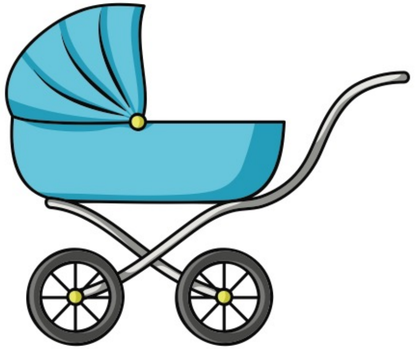
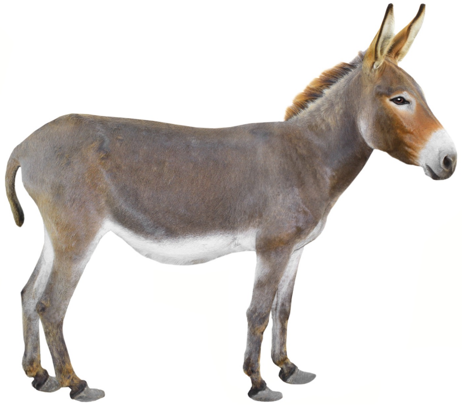
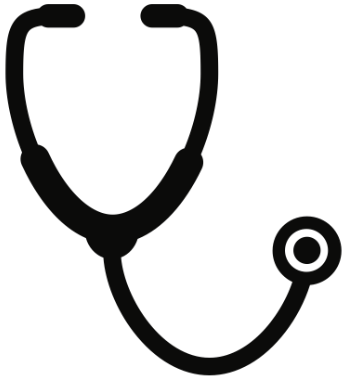

Answer: donkey, baby, battery, brush

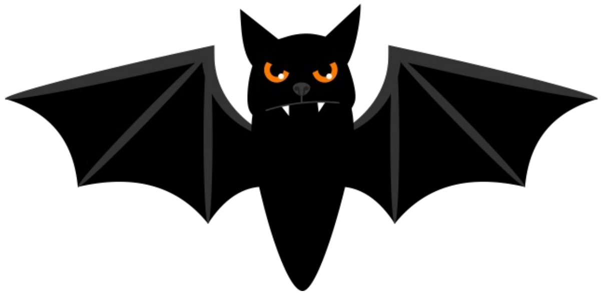
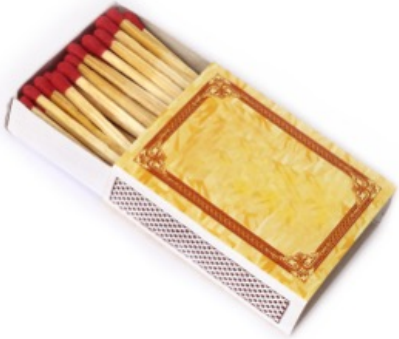
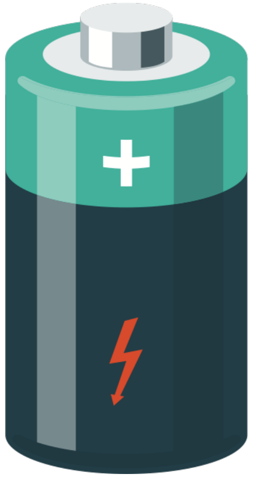
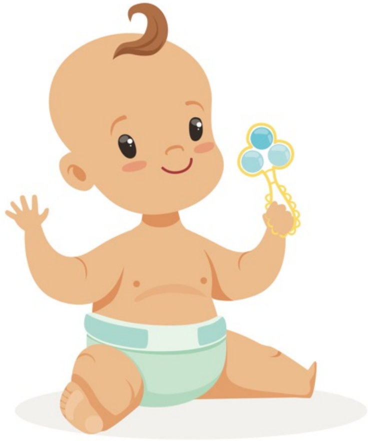

3. **Instruction:** Memorize the pictures in box A

BOX A

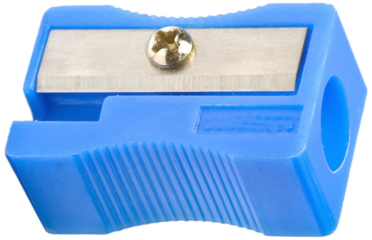

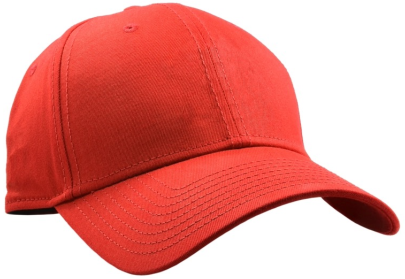

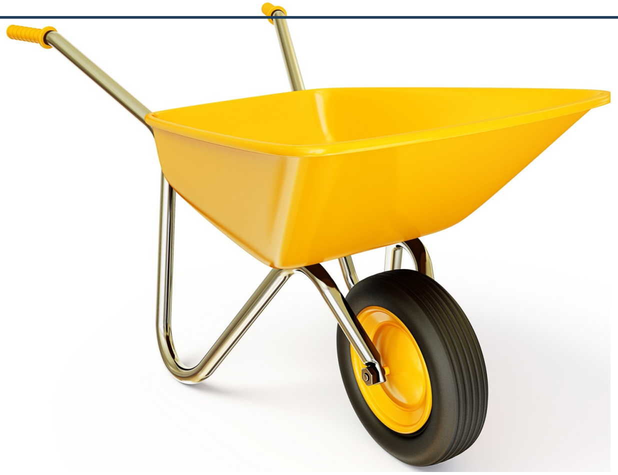

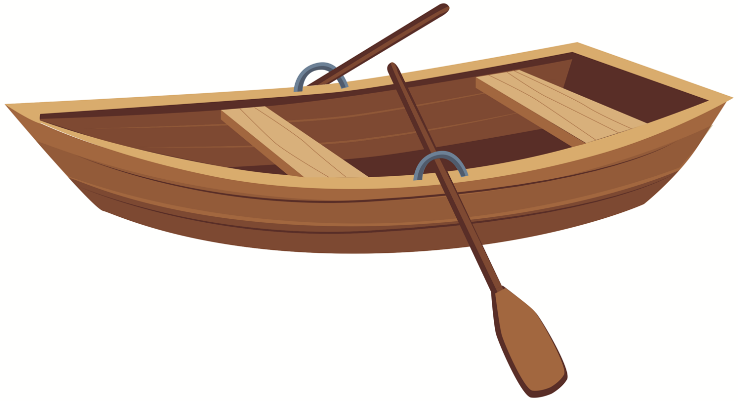

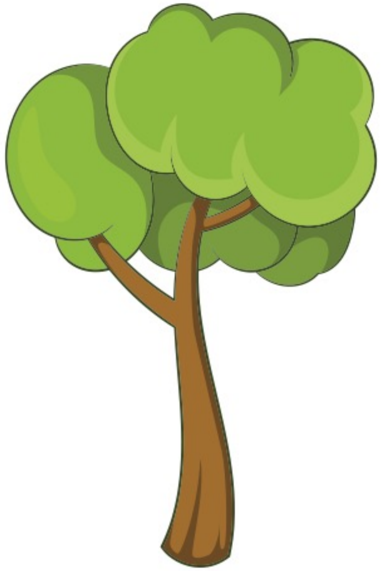

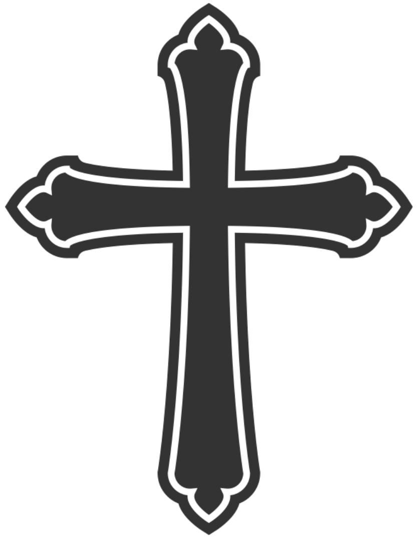

3. **Instruction:** Identify which pictures you recalled from box A and which are found in box B.

BOX B

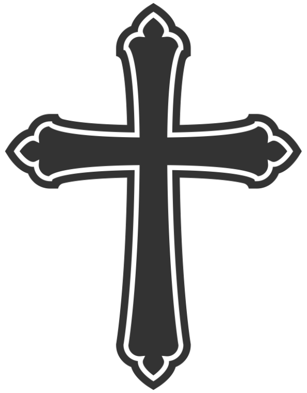
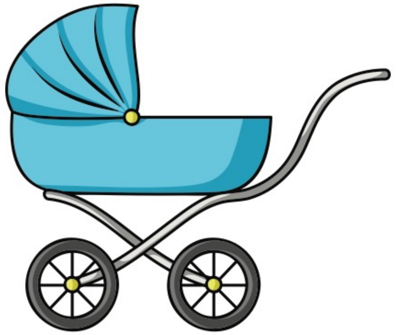
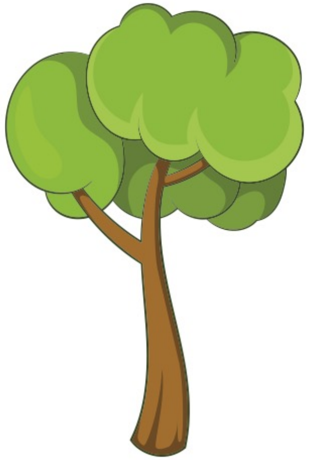
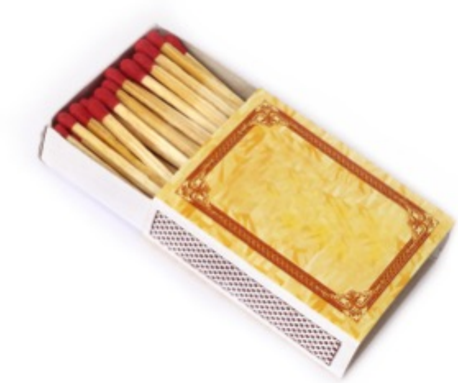
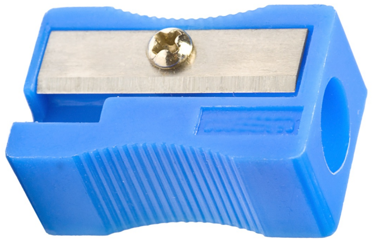
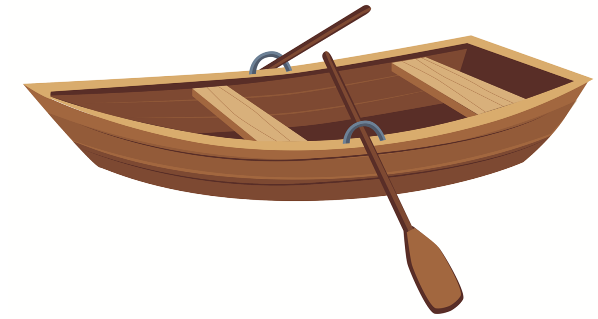
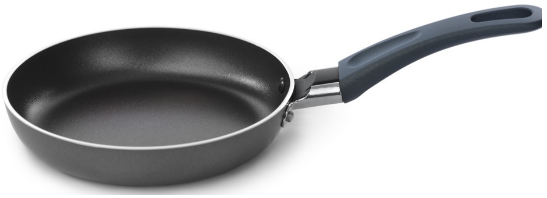
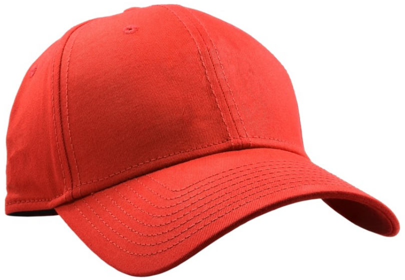
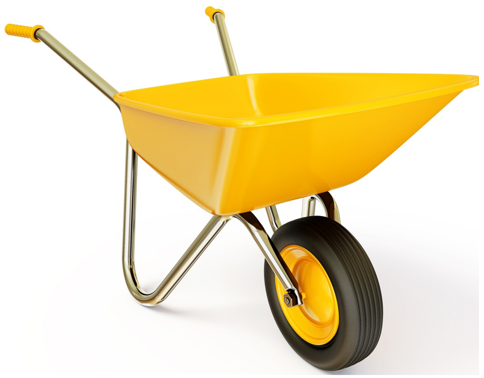

3. **Instruction:** Identify which pictures you recalled from box A and which are found in box B.

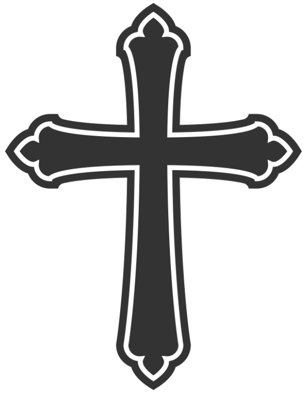
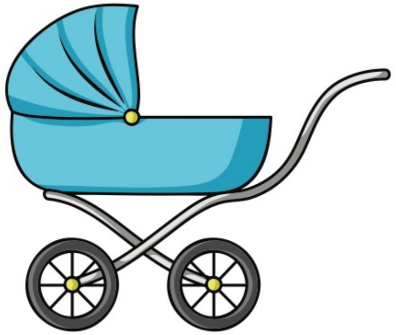
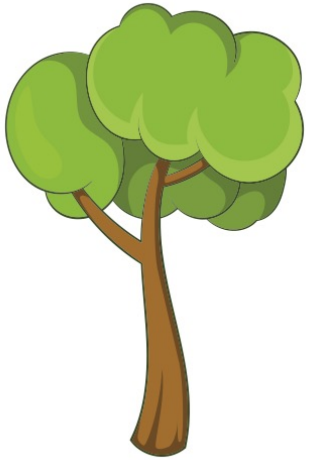
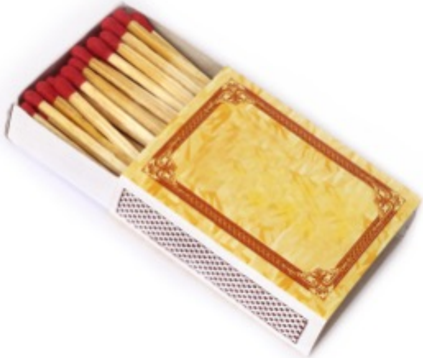
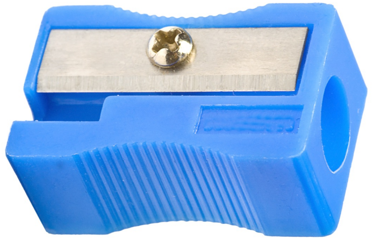

Answer: pencil sharpener, cap, pushcart/wheel barrow, boat, tree, cross

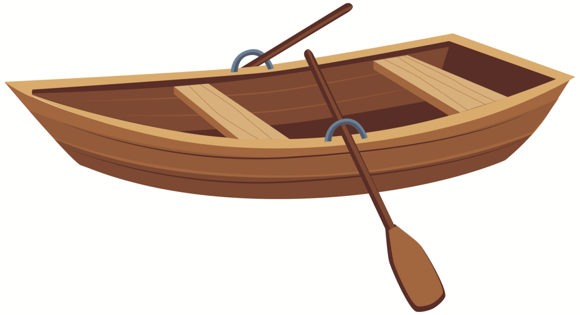
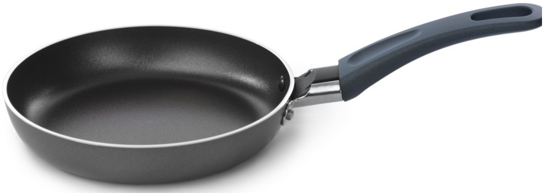
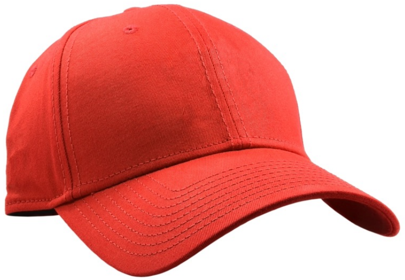
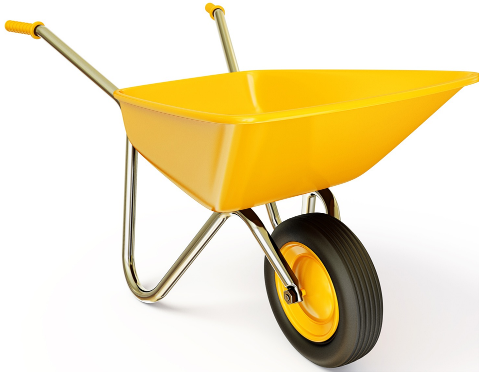

4. **Instruction:** Memorize the pictures in box A

BOX A

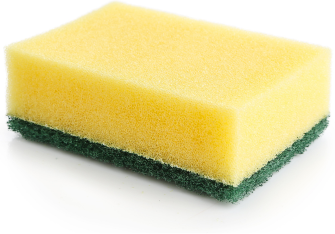

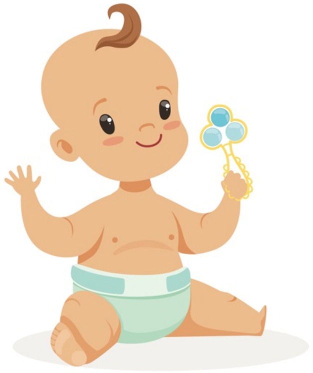

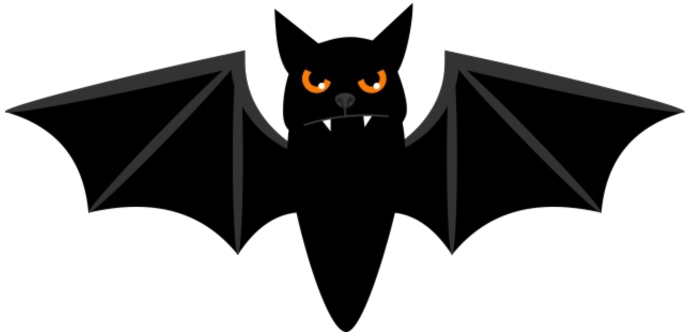

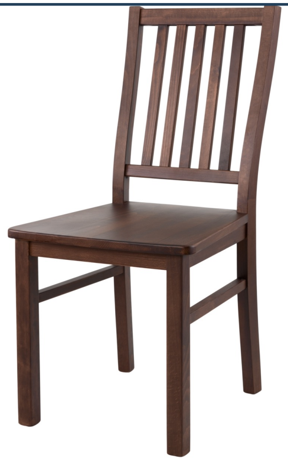

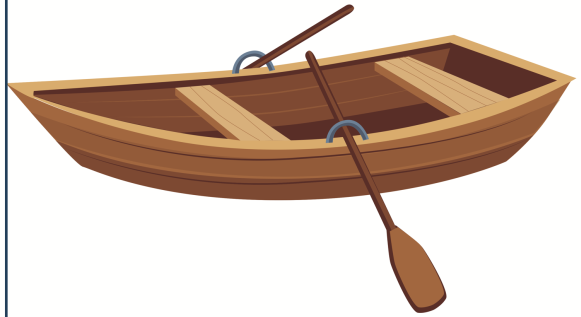

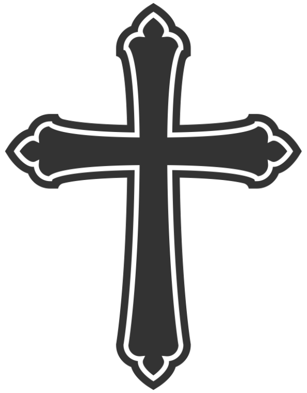

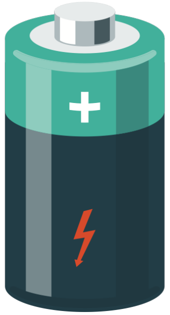

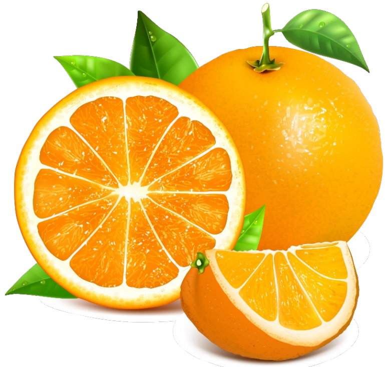

4. **Instruction:** Identify which pictures you recalled from box A and which are found in box B.

BOX B

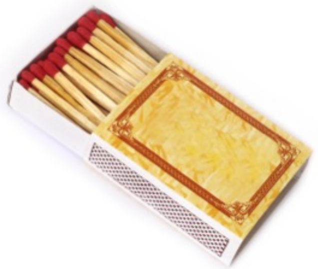
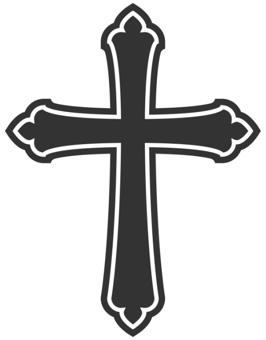
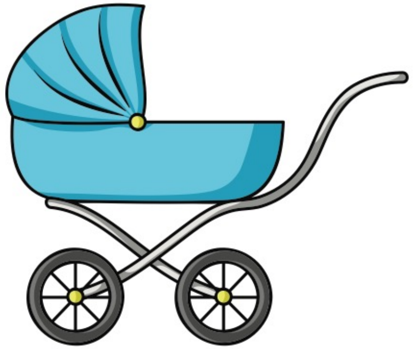
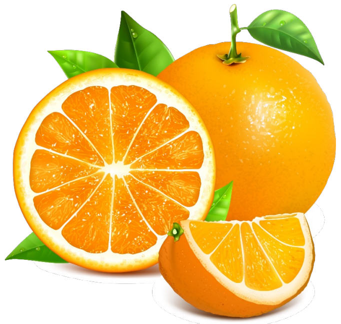
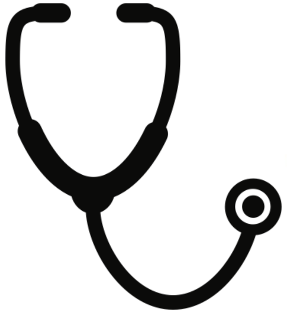
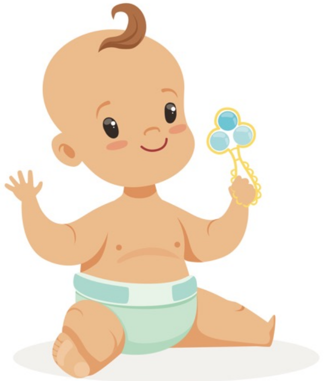
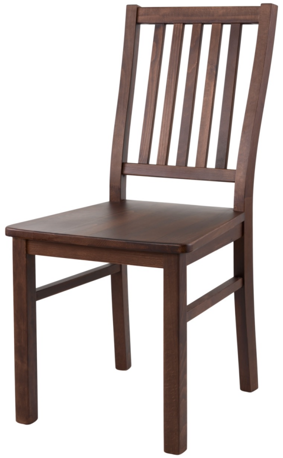
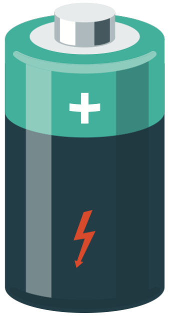
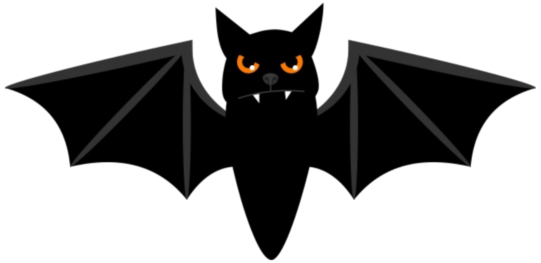
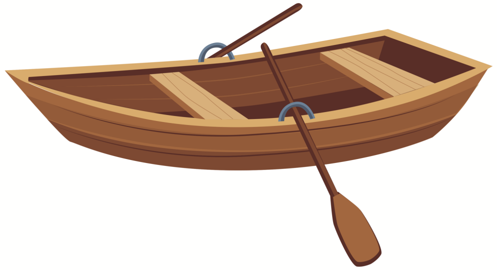
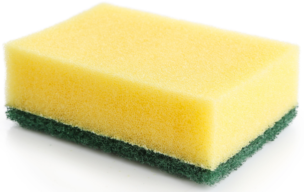

4. **Instruction:** Identify which pictures you recalled from box A and which are found in box B.

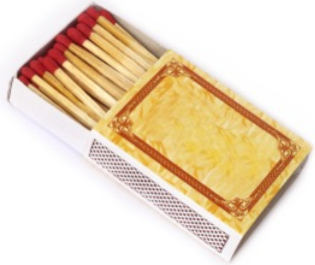
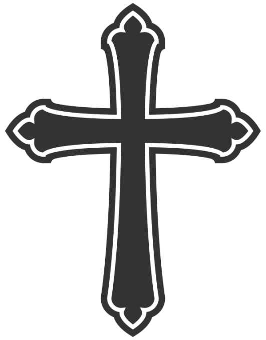
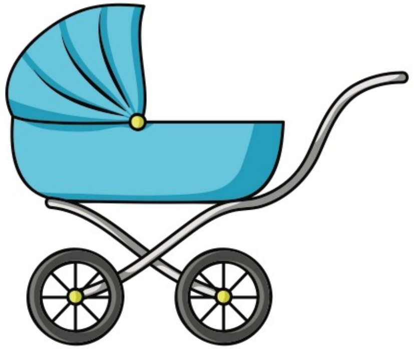
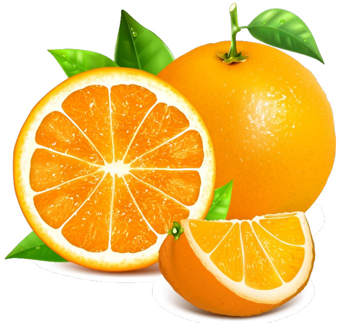
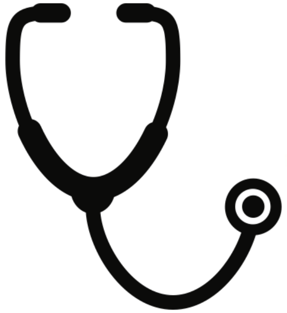
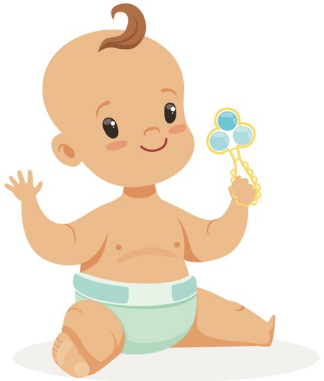

Answer: sponge, baby, bat, chair, boat, cross, battery, orange

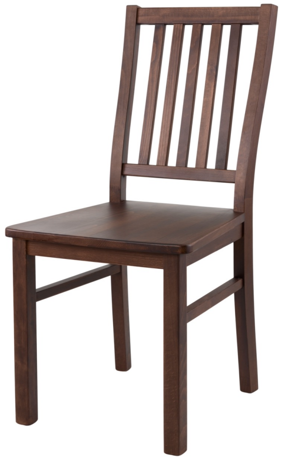
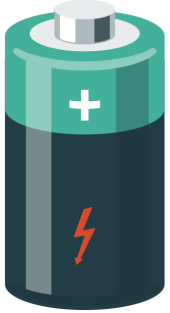
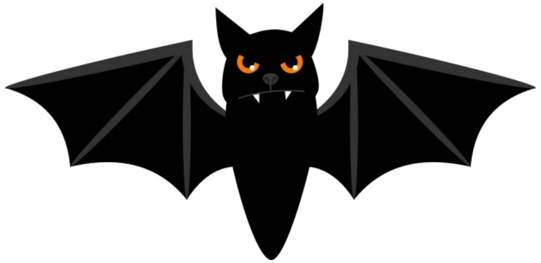
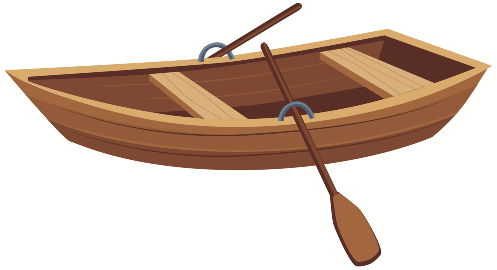
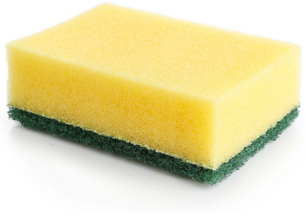

5. **Instruction:** Memorize the pictures in box A

BOX A

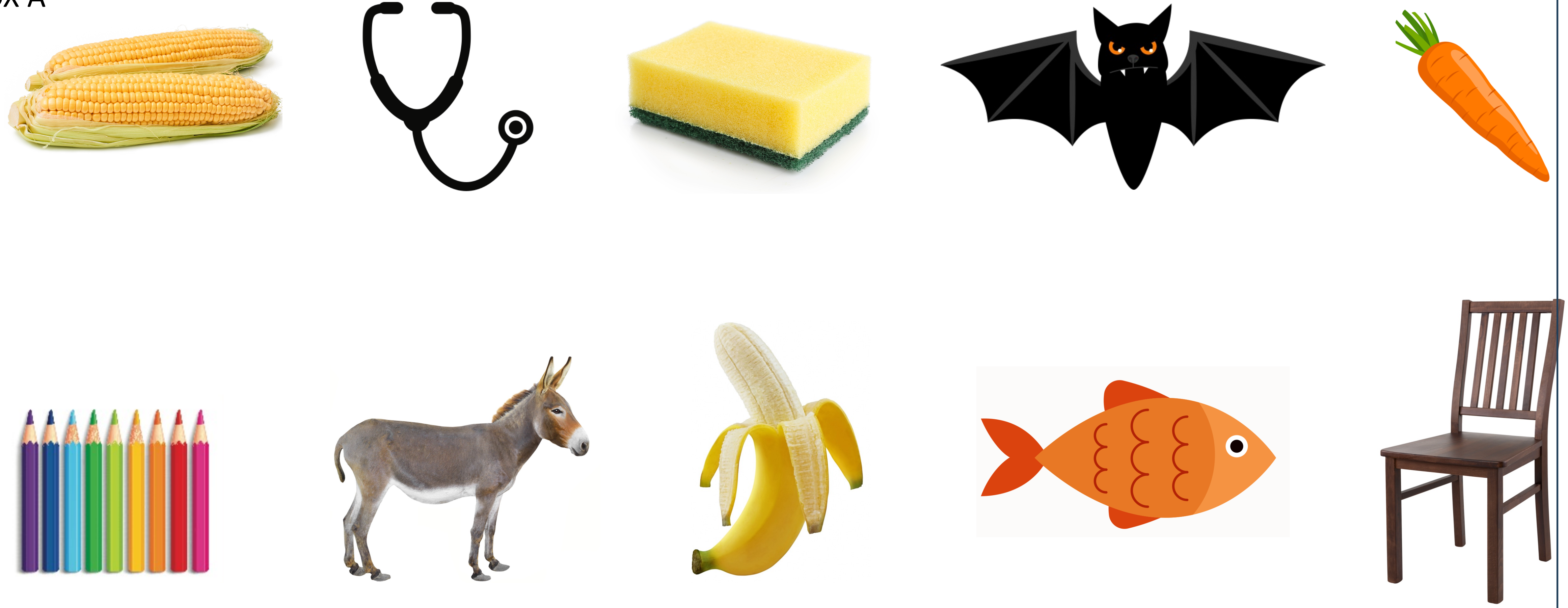

5. **Instruction:** Identify which pictures you recalled from box A and which are found in box B.

BOX B

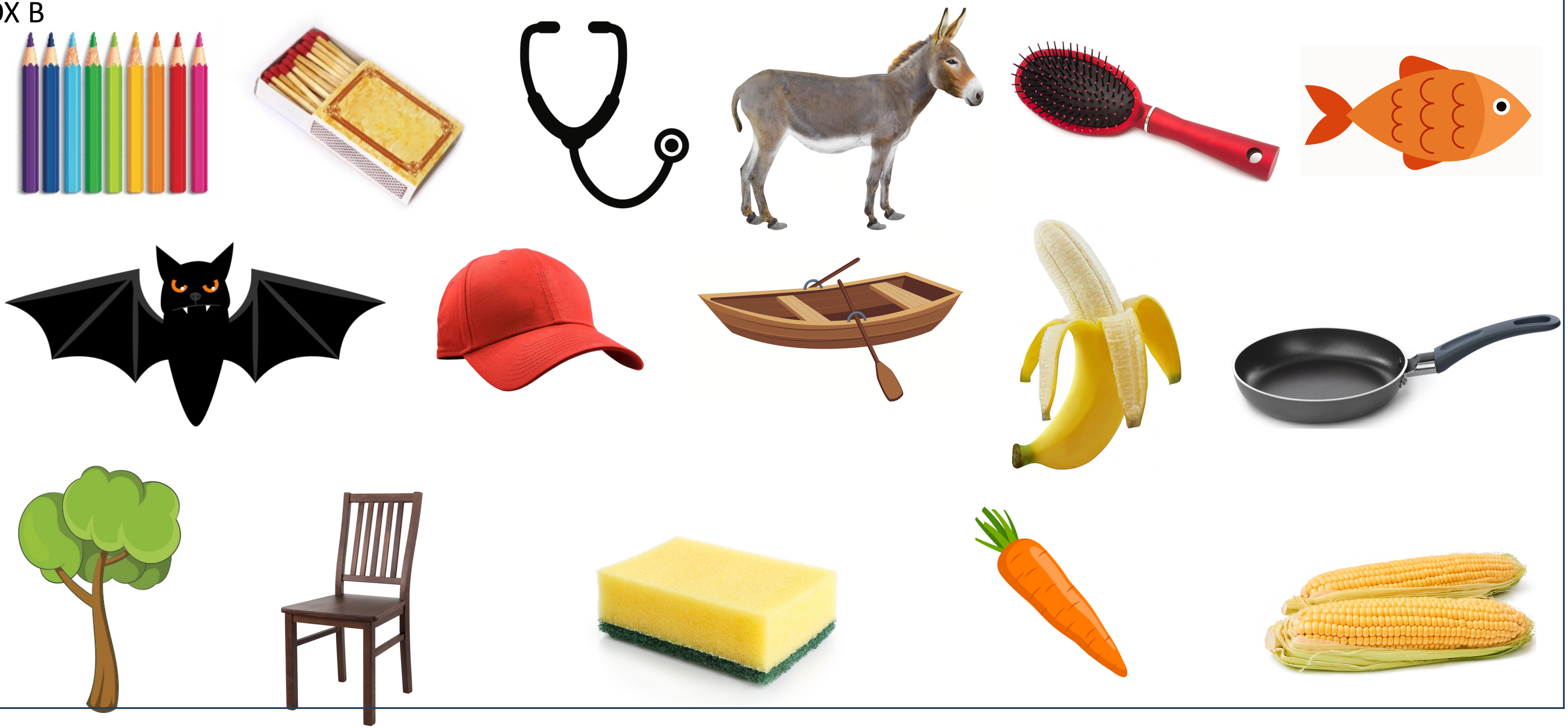

5. **Instruction:** Identify which pictures you recalled from box A and which are found in box B.

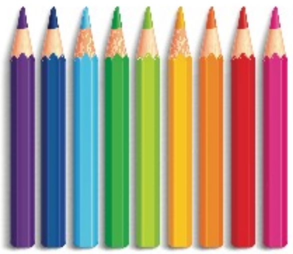
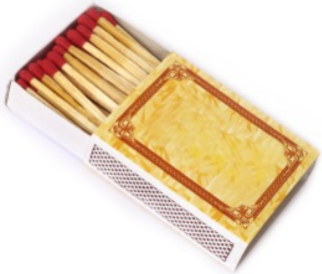
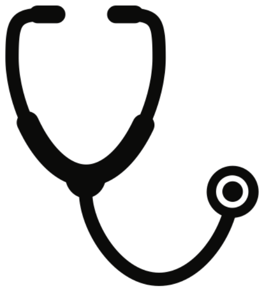
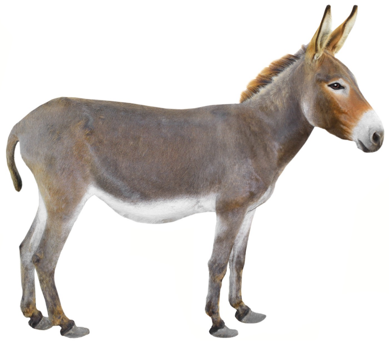
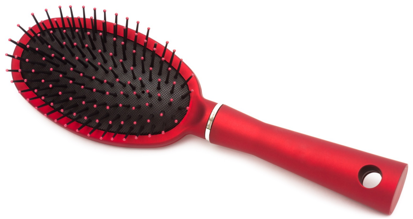
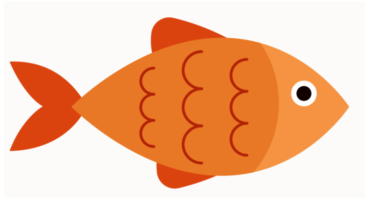
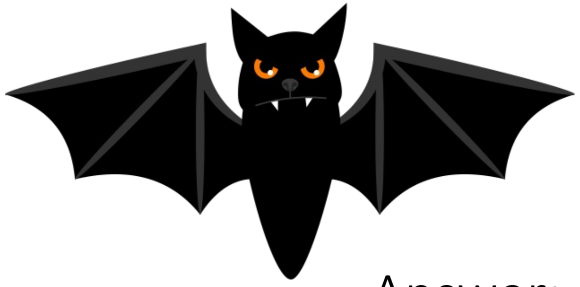
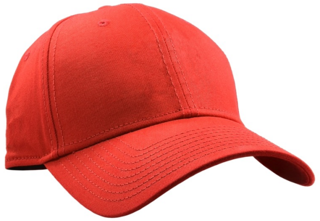
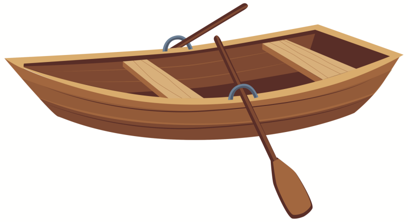
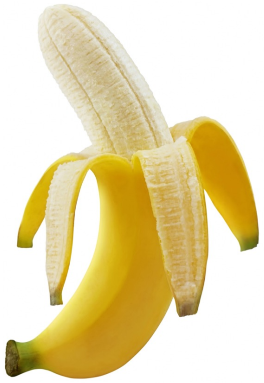
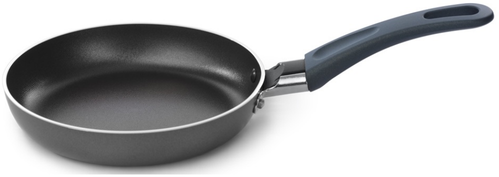

Answer: corn, stethoscope, sponge, bat, carrot, color pencil, donkey, banana, fish, chair

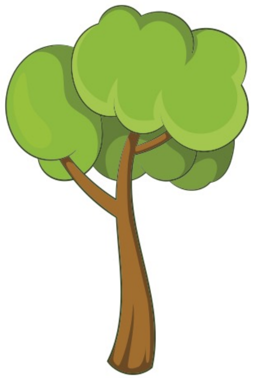
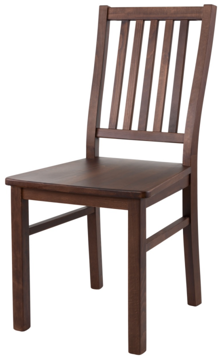
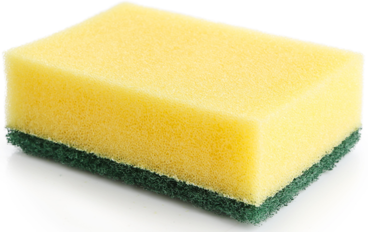
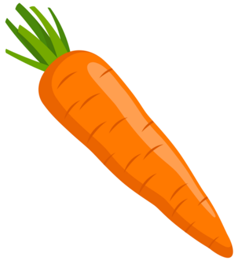
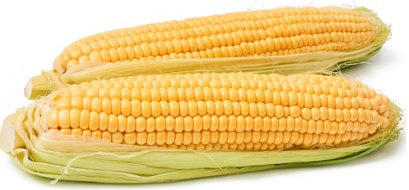

READING

Instruction: Read the following sentences

Instruction: Read the following sentences

1. She sells seashells by the sea shore
2. Fresh fried fish, fish fresh fried, fried fish fresh, fish fried fresh.
3. I scream, you scream, we all scream for ice cream!
4. I saw a kitten eating chicken in the kitchen.
5. If two witches were watching two watches, which witch would watch which watch.

Word Formation

Instruction: Form the word correctly

1. NET – PLA
2. PU – TER – COM
3. VI – TE – SI - ON – LE

Word Formation

Instruction: Form the word correctly

1. NET – PLA
2. PU – TER – COM
3. VI – TE – SI - ON – LE

Answer: planet, computer, television

4. **Instruction:** The word COLOR, just doesn't fit in:

a) in b)ful c)less

5. **Instruction:** Where could you buy a PAPER?

4. **Instruction:** The word COLOR, just doesn't fit in:

a) in b)ful c)less

Answer: a

5. **Instruction:** Where could you buy a PAPER?

Answer: stationery shop

DRAWING

Instruction: Draw the following shapes on the answer sheet

Instruction: Draw the following shapes on the answer sheet

1. Circle
2. Triangle
3. Cube

Instruction: Copy the following forms on the answer sheet

4.

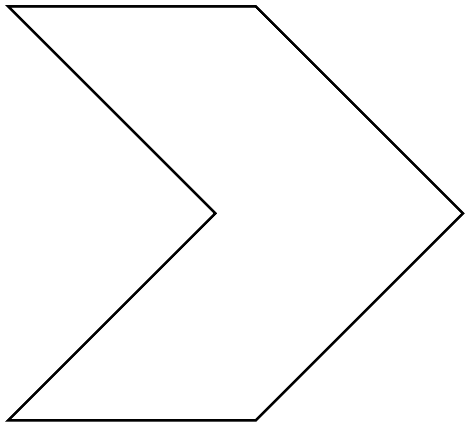

5.

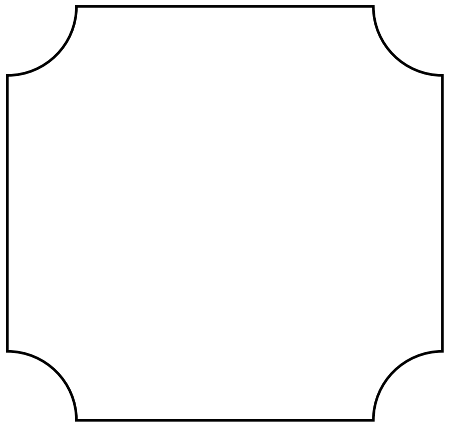

SPELLING

Instruction: Spell the words in the forward or the reverse order

Spell in the forward order

1. ARC
2. WHALE
3. SANDWICH

Spell in the reverse order

4. ADORE
5. CONTRACT
