# Supplementary material for: Filling the gap: brief neuropsychological assessment protocol for glioma patients undergoing awake surgeries
Source: Front Psychol. 2024 Aug 9;15:1417947. doi: 10.3389/fpsyg.2024.1417947 (PMC11342098; doi:10.3389/fpsyg.2024.1417947)
Supplement: Supplementary file 5 [file Data_Sheet_5.PDF]

OMFTCT

Ohy-Maldaun Fast Track Cognitive Test

POSTOPERATIVE (T2)

NAMING

Instruction: Say the name of the figure

1. **Instruction:** Say the name of this figure

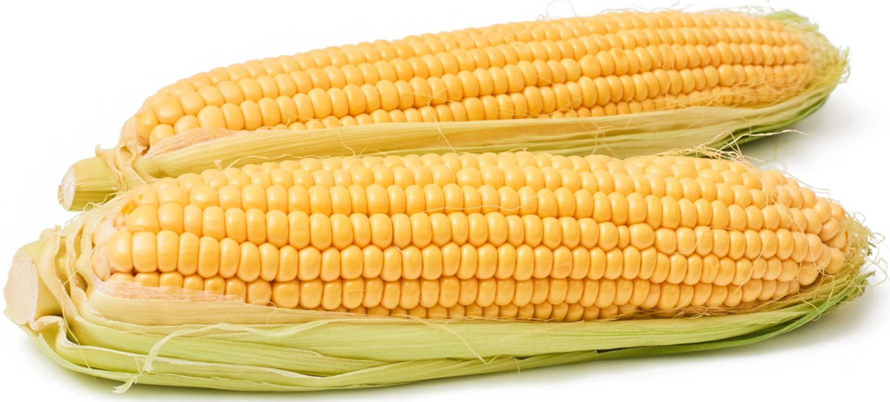

1. **Instruction:** Say the name of this figure

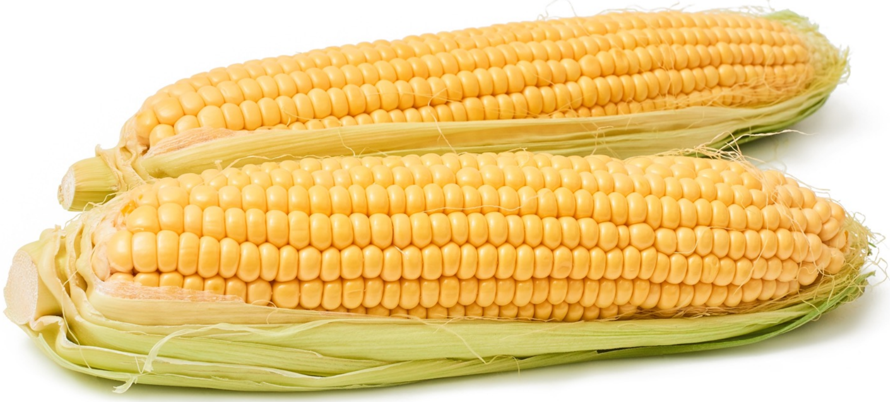

Answer: corn/corn cob

2. **Instruction:** Say the name of this figure

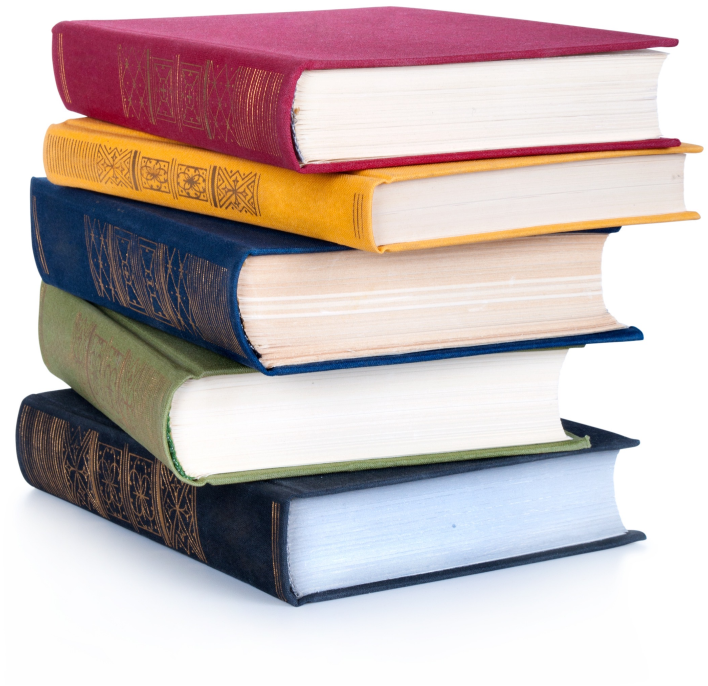

2. **Instruction:** Say the name of this figure

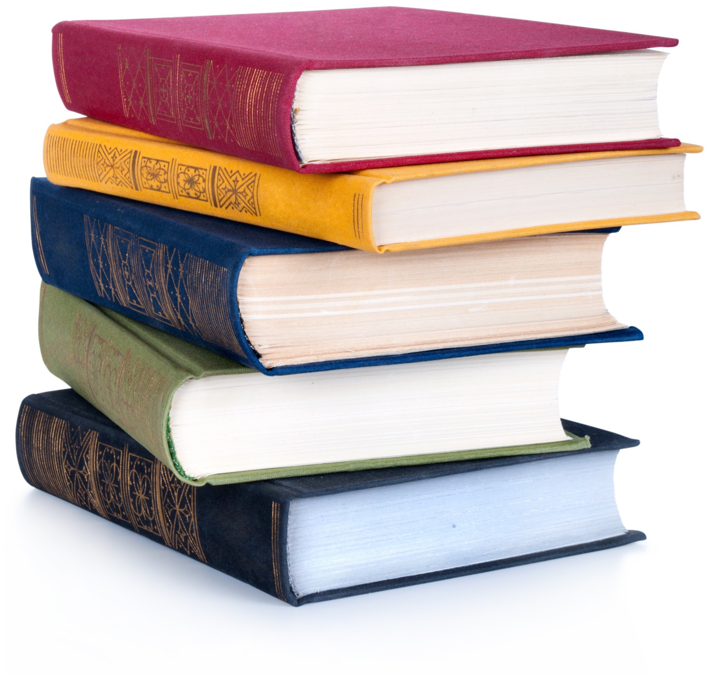

Answer: books

3. **Instruction:** Say the name of this figure

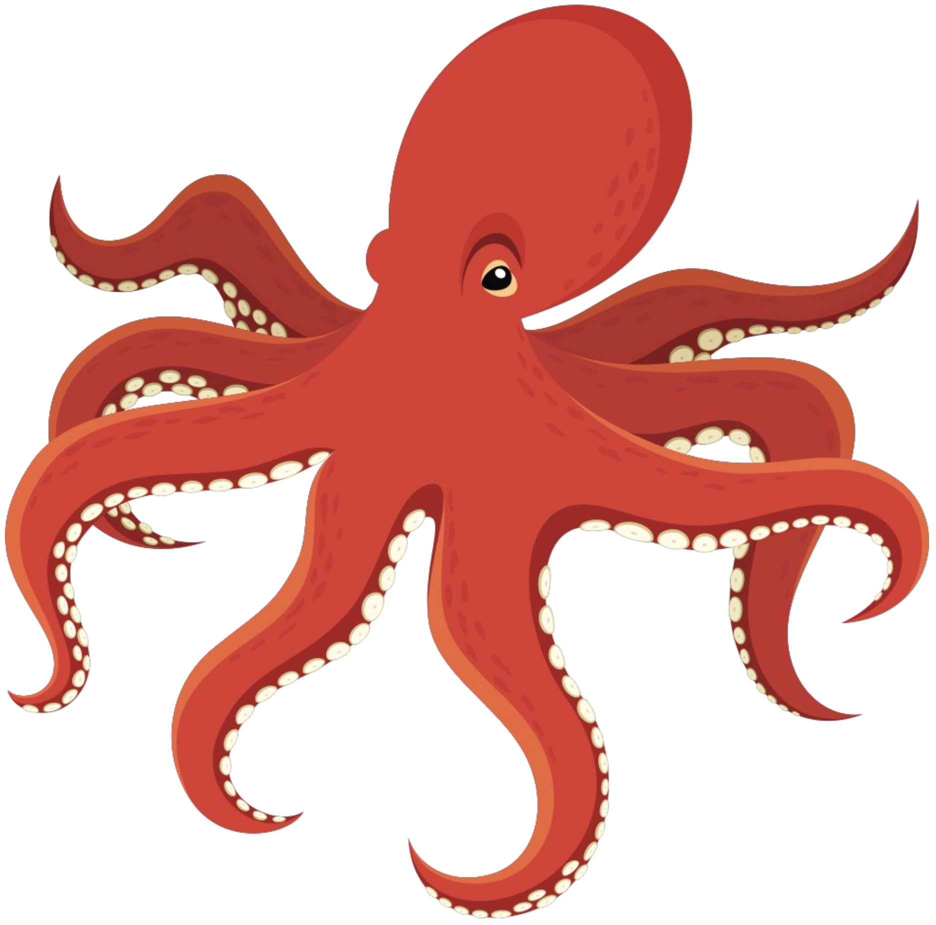

3. **Instruction:** Say the name of this figure

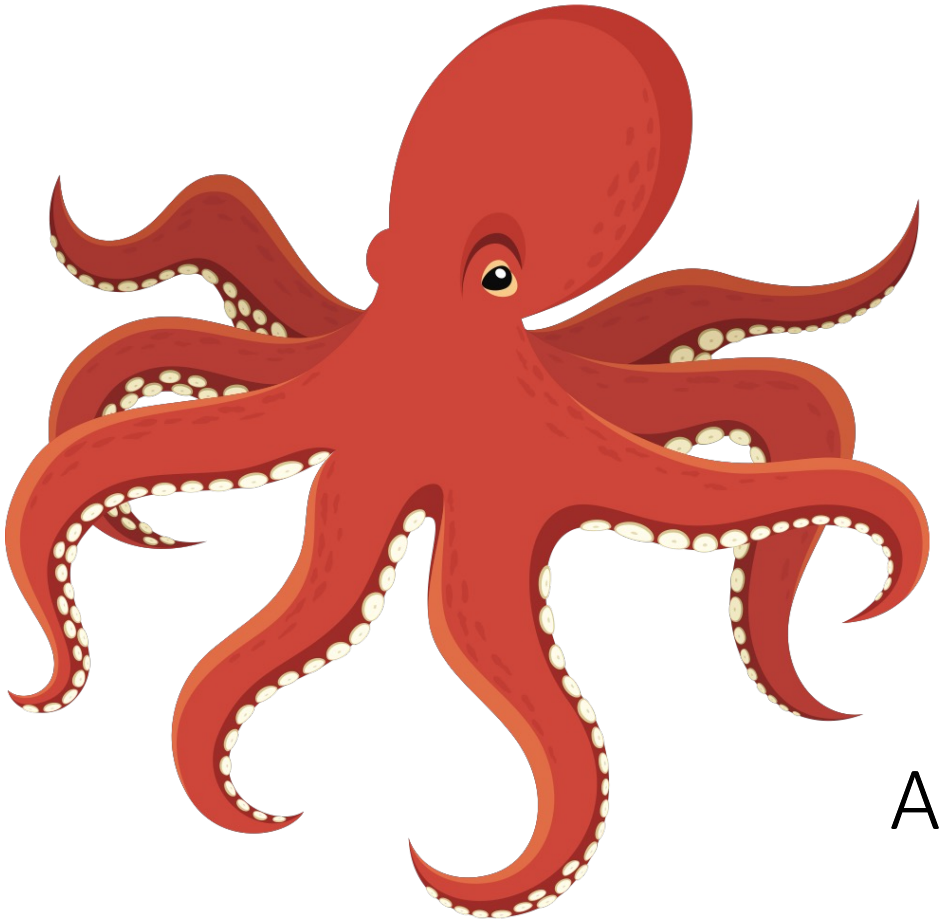

Answer: octopus

Dual task naming

Instruction: Open and close your hands alternautely while naming the figure

4. **Instruction:** Say the name of the figure while opening and closing your hands.

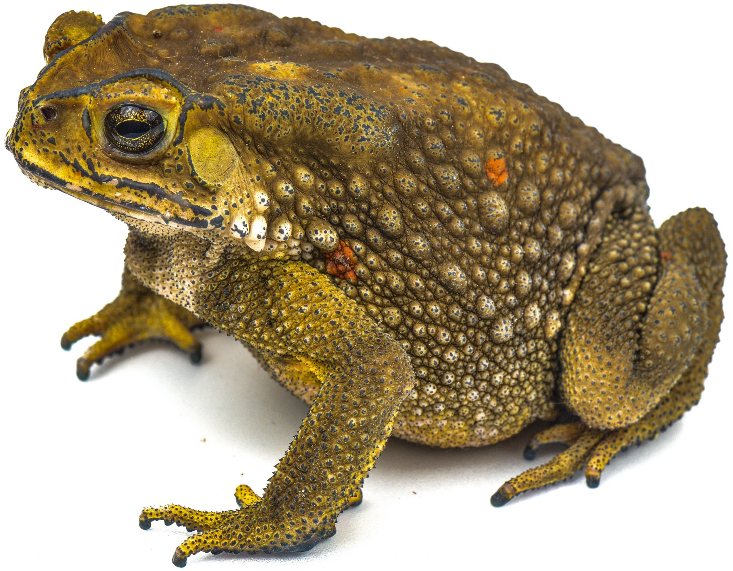

4. **Instruction:** Say the name of the figure while opening and closing your hands.

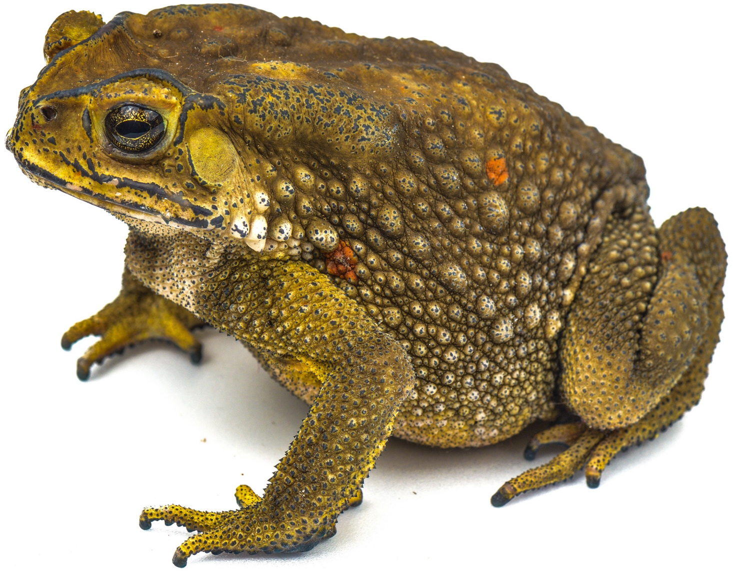

Answer: frog

5. **Instruction:** Say the name of the figure while opening and closing your hands.

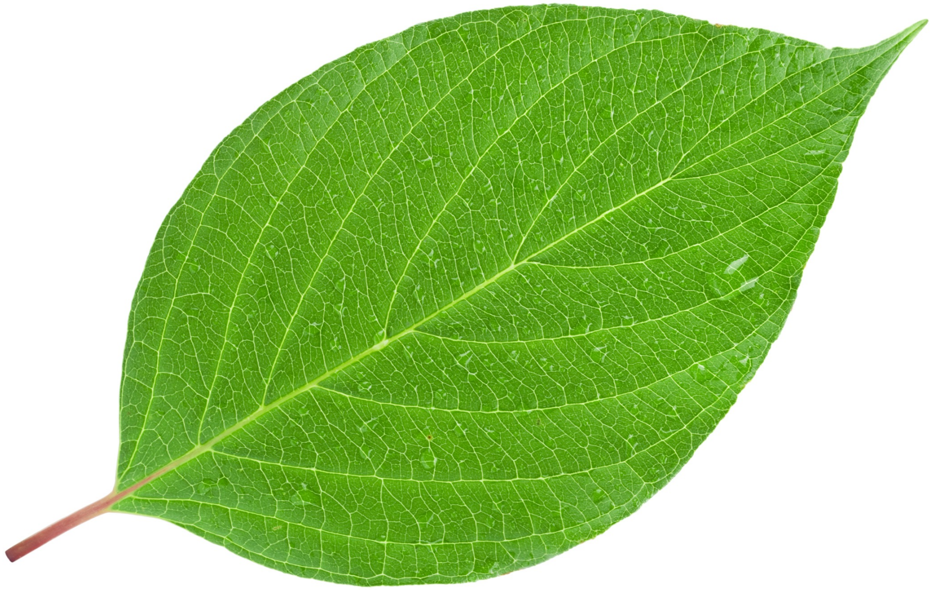

5. **Instruction:** Say the name of the figure while opening and closing your hands.

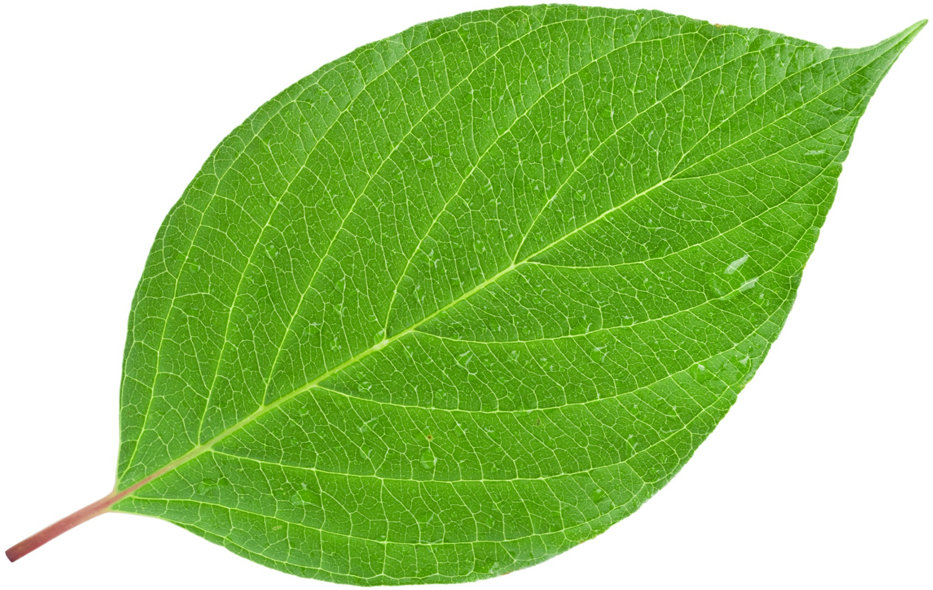

Answer: leaf

VERBAL MEMORY

Instructions: Memorize and repeat what is said

VERBAL MEMORY

Instruction: Memorize and repeat what is said:

1. Gray is the color of the rat that entered the house.
2. The gray rat entered the yellow house that had a door.
3. Shoes – swallow – plum – glove
4. 4 – 0 – 1 – 9 – 7
5. Q – J – D – I – V – O

SEMANTIC

Instruction: Say which figure at the bottom relates to the main figure at the top.

1. **Instruction:** Say which figure at the bottom relates to the main figure at the top.

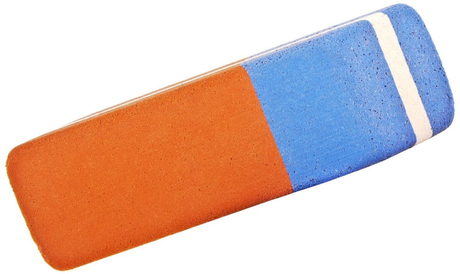

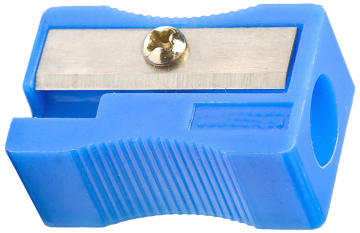

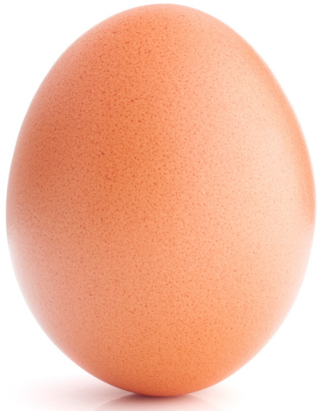

1. **Instruction:** Say which figure at the bottom relates to the main figure at the top.

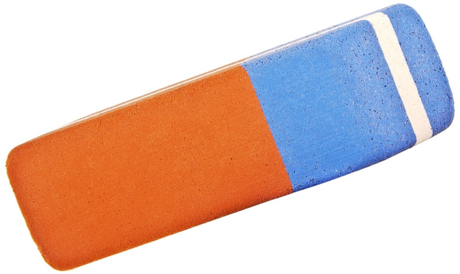

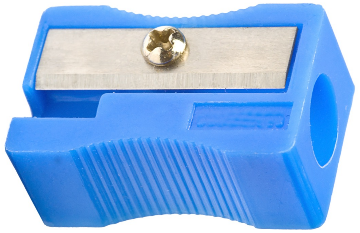

Answer: sharpener

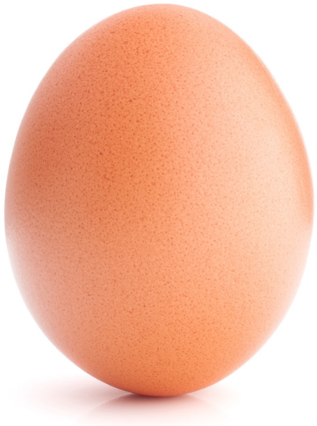

2. **Instruction:** Say which figure at the bottom relates to the main figure at the top.

water

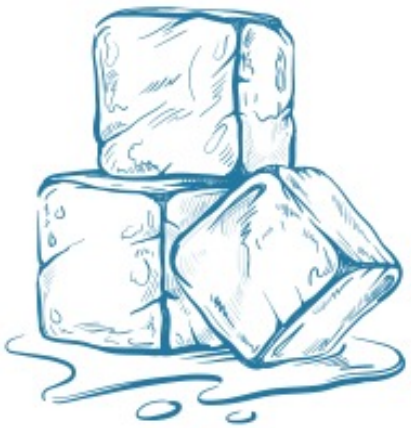

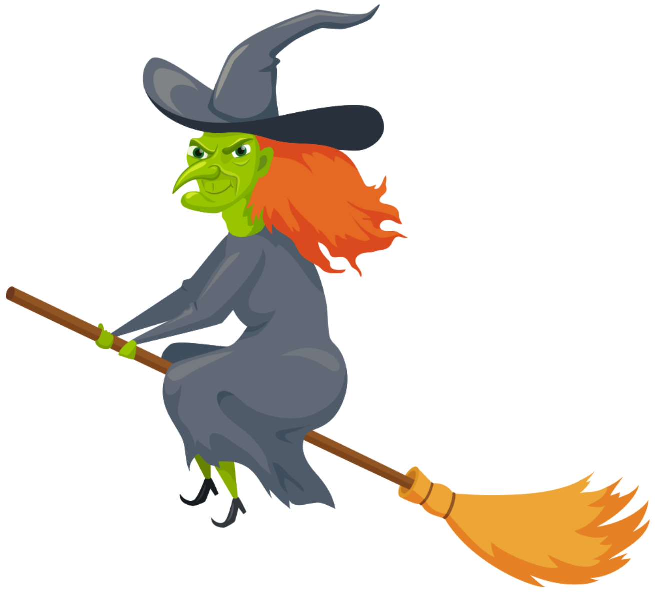

2. **Instruction:** Say which figure at the bottom relates to the main figure at the top.

water

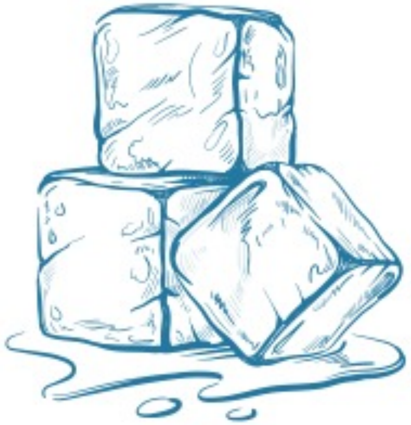

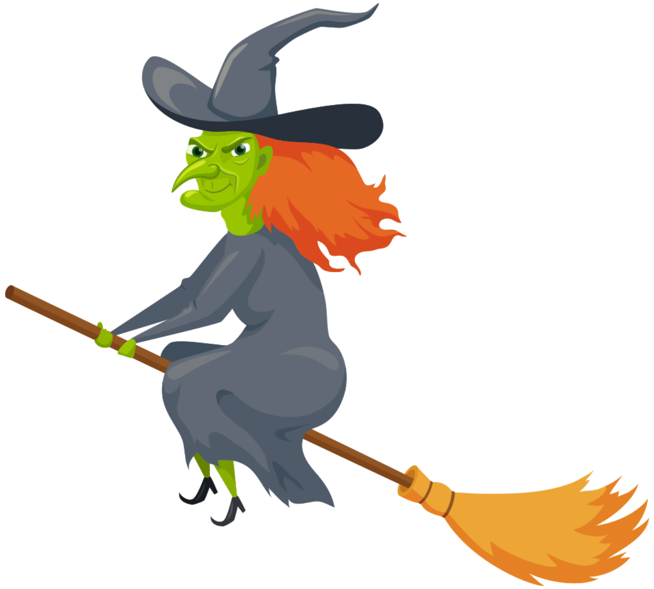

Answer: ice

Dual task semantics

Instruction: Touch your right ear with your left hand alternately while answering the task

3. **Instruction:** Touch your right ear with your left hand alternately while answering the question.

What is it for?

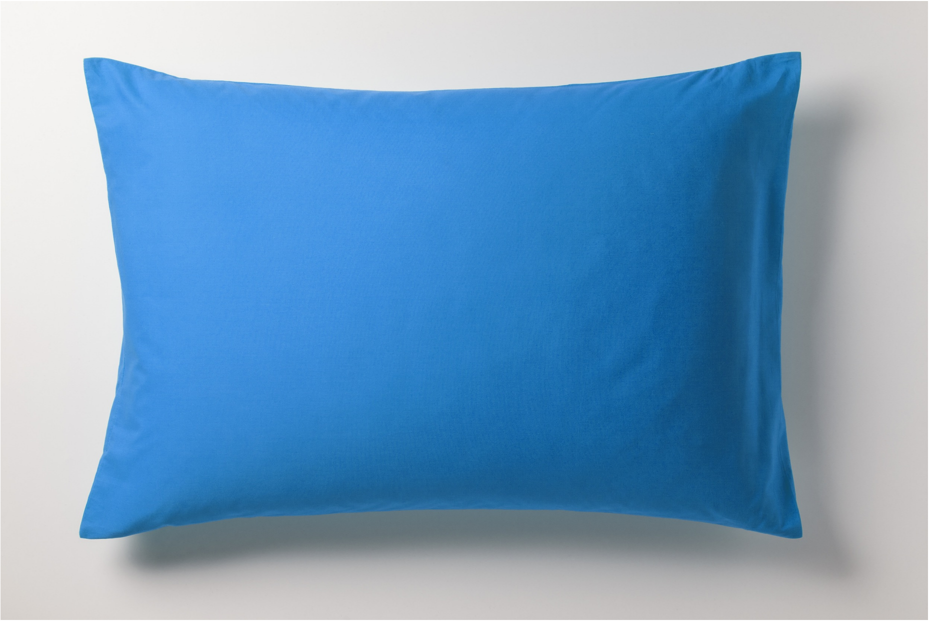

3. **Instruction:** Touch your right ear with your left hand alternately while answering the question.

What is it for?

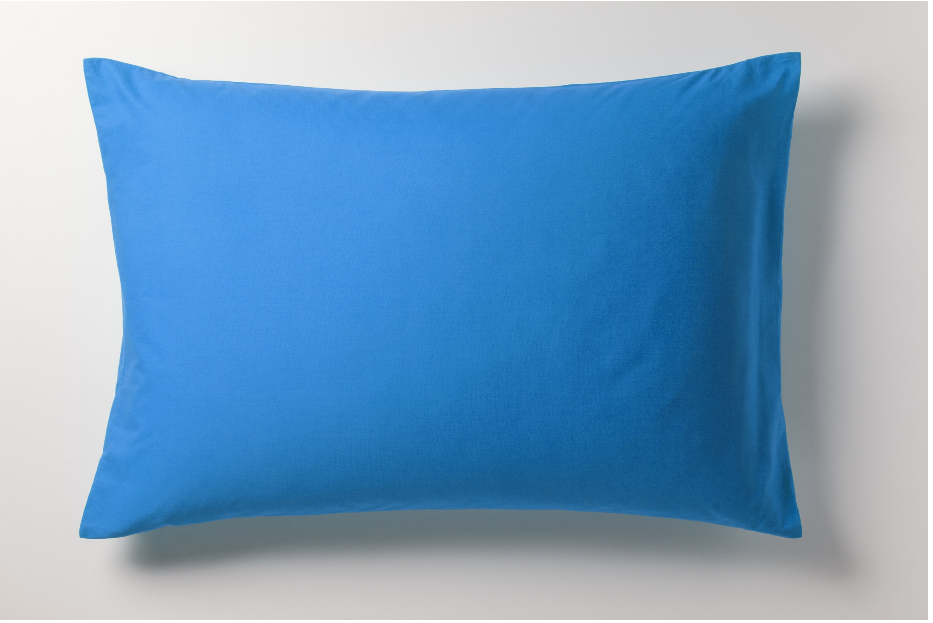

Answer: lie down / rest / sleep

4. **Instruction:** Touch your right ear with your left hand alternately while answering the question.

What material is it made of?

KEY

4. **Instruction:** Touch your right ear with your left hand alternately while answering the question.

What material is it made of?

KEY

Answer: metal

5. **Instruction:** Touch your right ear with your left hand alternately while answering the question.

What shape does it have?

STRAW

5. **Instruction:** Touch your right ear with your left hand alternately while answering the question.

What shape does it have?

STRAW

Answer: cilinder

CALCULATION

Instruction: Calculate and tate the result

Instructions: Calculate and tell the result

1. 7 + 4 = 11

2. 15 – 8 = 7

3. 23 x 2 = 46

4. **Instruction:** Add only the unique numbers and subtract 3 from the final result.

1 – 7 – 3 – 2 – 1 – 8

5. **Instruction:** Subtract 3 from 20, then continue subtracting 3 four more times consecutively from the previous result.

4. **Instruction:** Add only the unique numbers and subtract 3 from the final result.

1 – 7 – 3 – 2 – 1 – 8

Answer: 17

5. **Instruction:** Subtract 3 from 20, then continue subtracting 3 four more times consecutively from the previous result.

Answer: 17, 14, 11, 8

WRITING

Intruction: Copy and transcribe the words and sentences

Instruction: Copy the words

1. Fantasy
2. Rubber

Instruction: Copy the sentence

3. Currently, it fulfills its commitments in an organized manner.

Instruction: Write the dictated word

4. Salamander

Instruction: Write the dictated sentence

5. The trip happened a long time ago, but it left good memories.

VISUAL MEMORY

Instructions: Examine the pictures in box A and then identify which images from box B were present in box A.

1. **Instruction:** Memorize the pictures in box A

BOX A

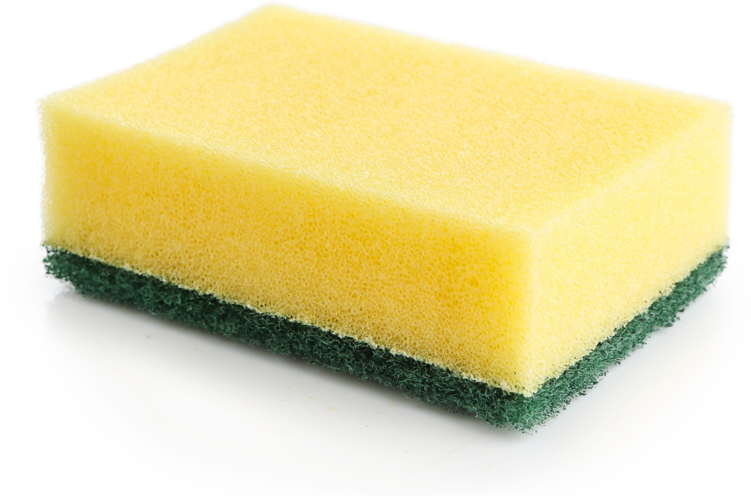

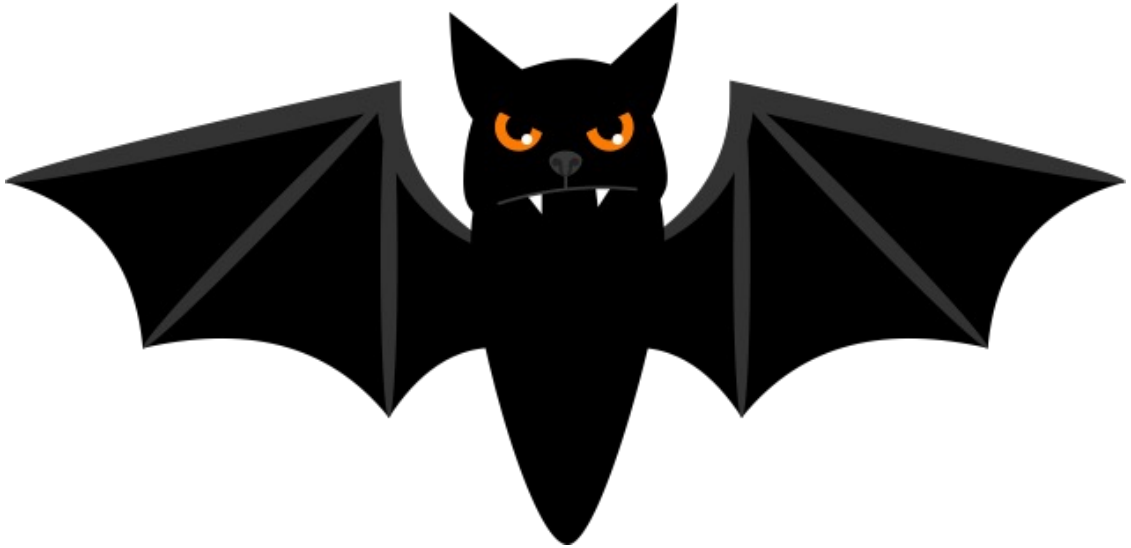

1. **Instruction:** Identify which pictures you recalled from box A and which are found in box B.

BOX B

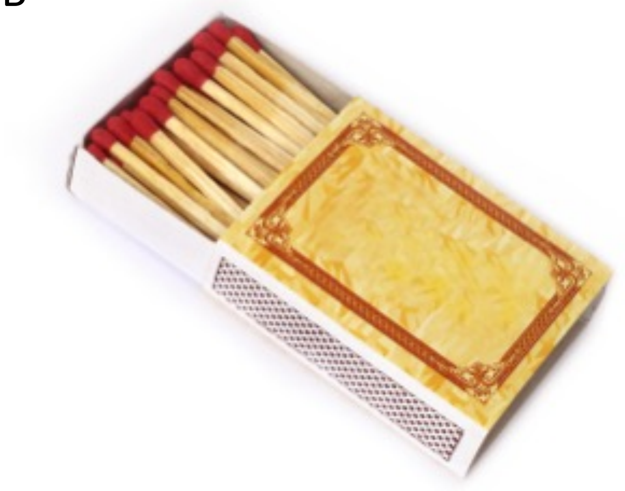

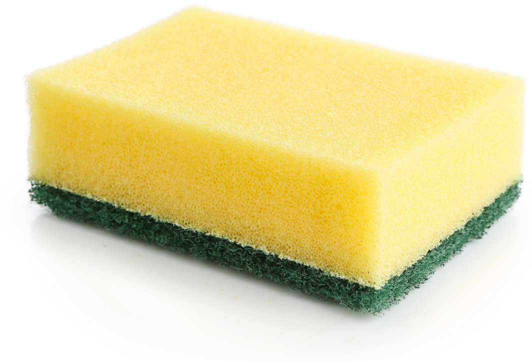

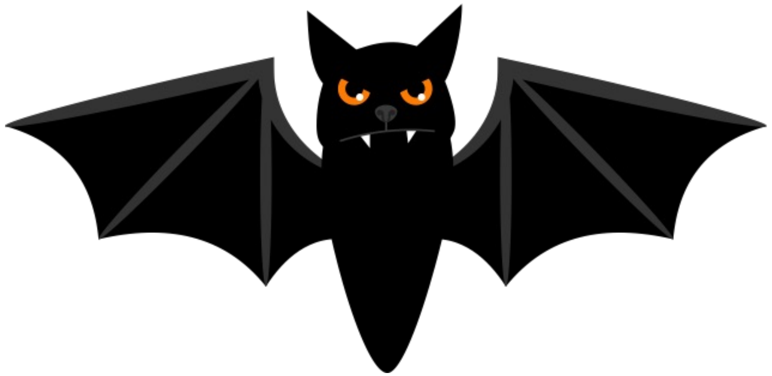

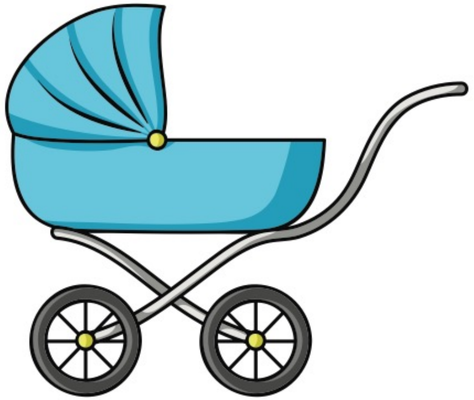

1. **Instruction:** Identify which pictures you recalled from box A and which are found in box B.

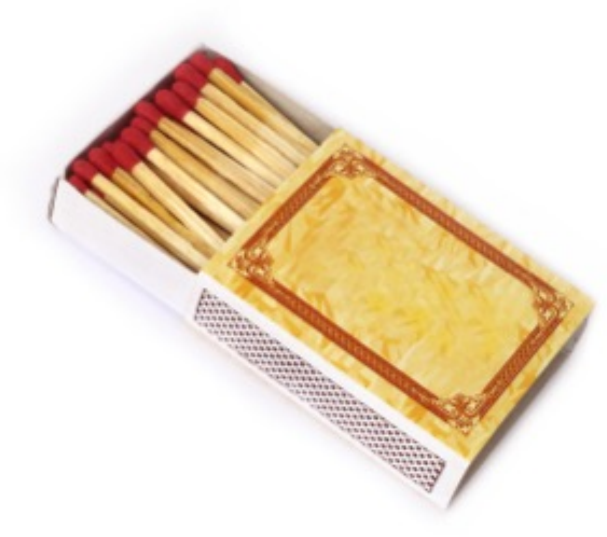

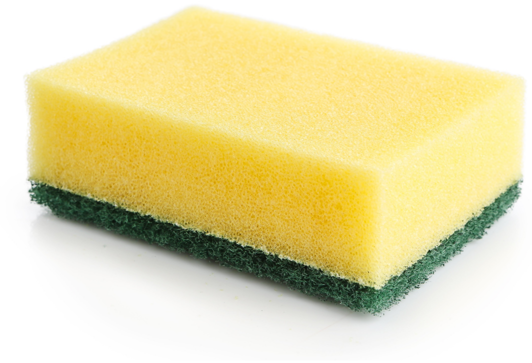

Answer: bat and sponge

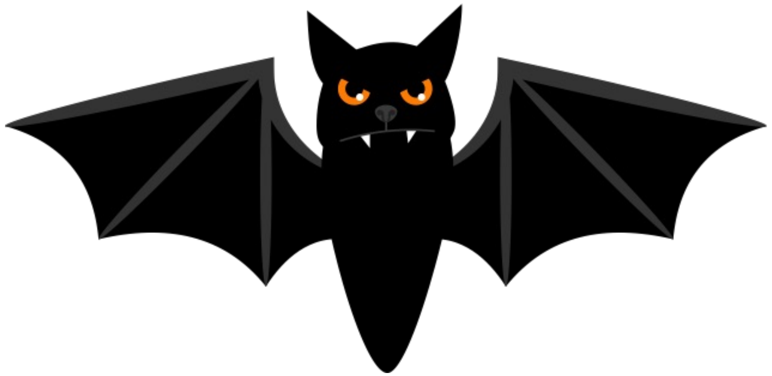

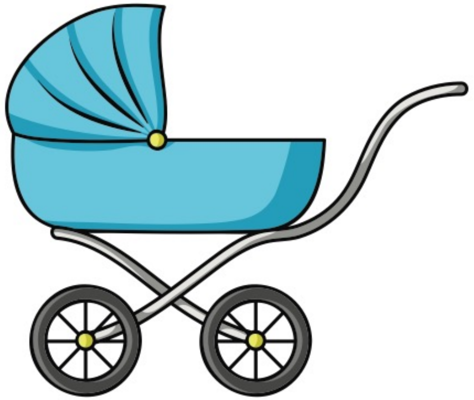

2. **Instruction:** Memorize the pictures in box A

BOX A

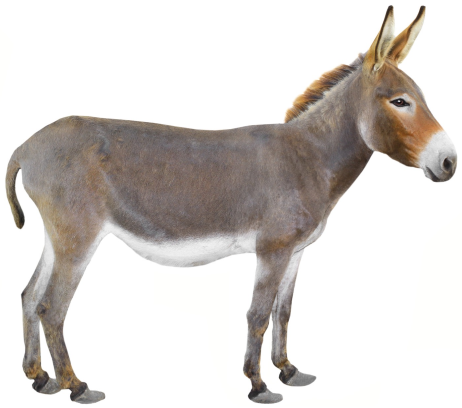 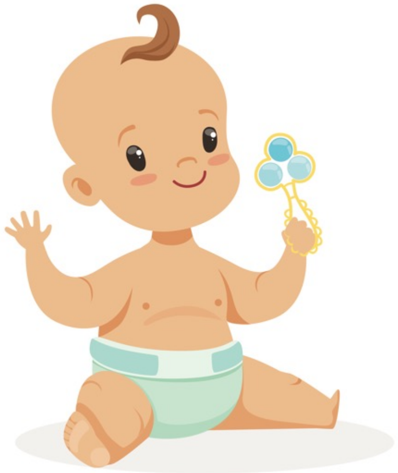 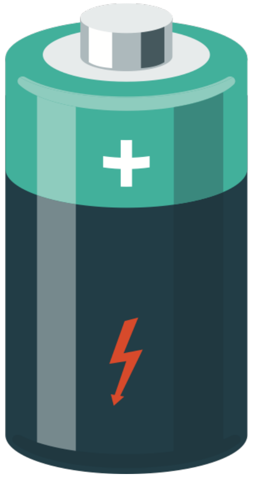 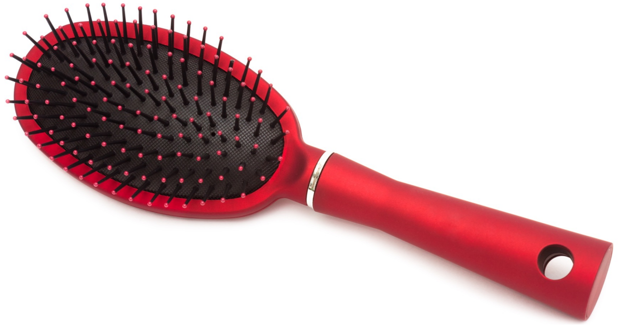

2. **Instruction:** Identify which pictures you recalled from box A and which are found in box B.

BOX B

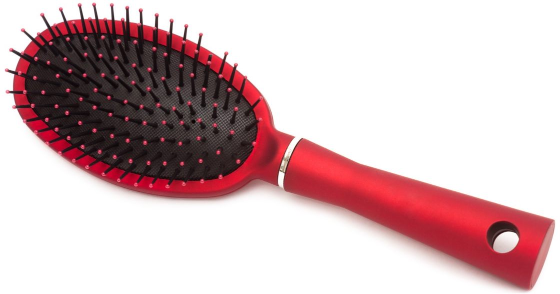
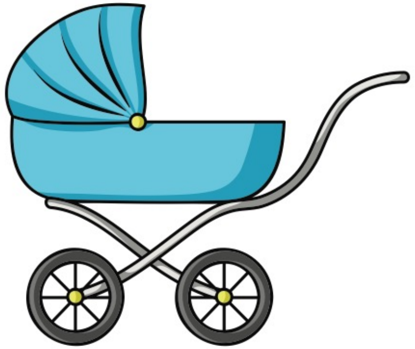
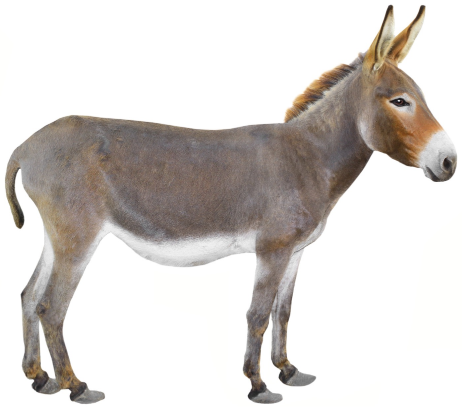
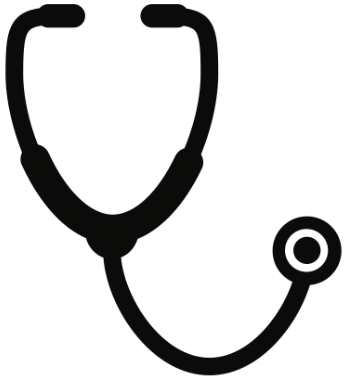
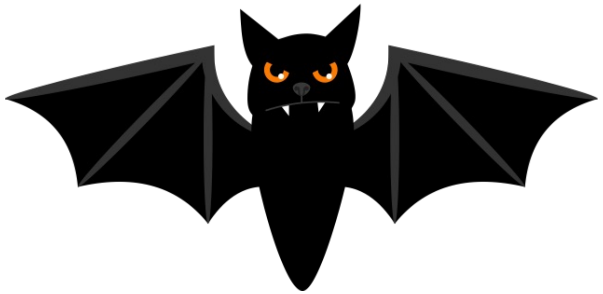
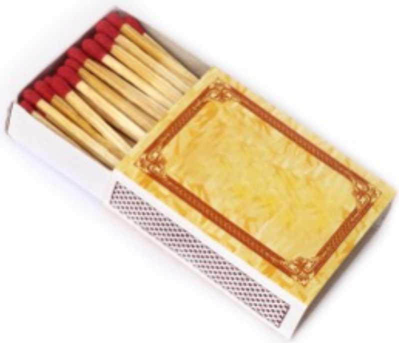
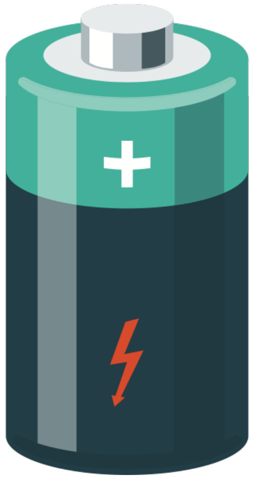
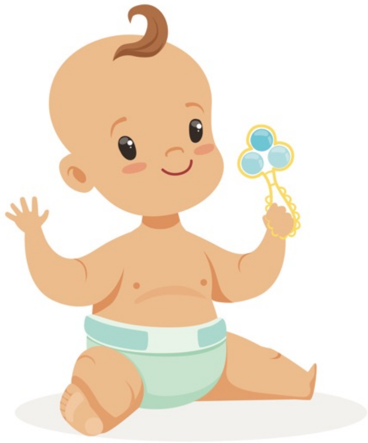

2. **Instruction:** Identify which pictures you recalled from box A and which are found in box B.

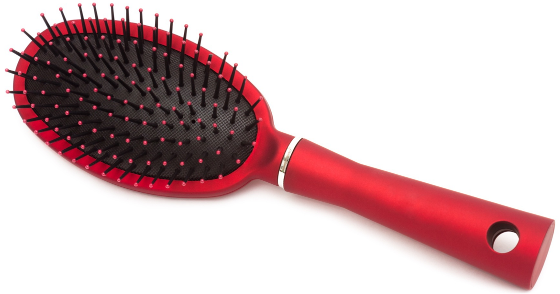
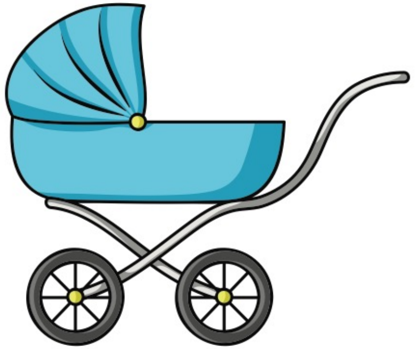
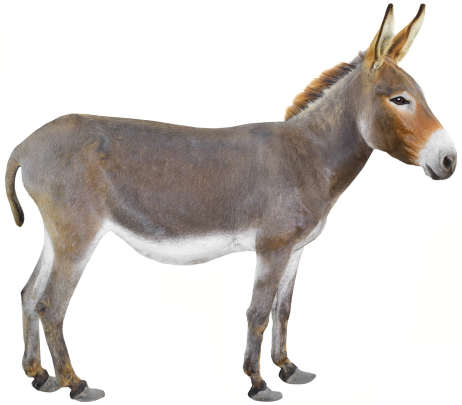
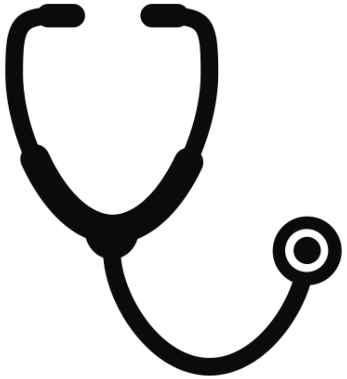

Answer: donkey, baby, battery, brush

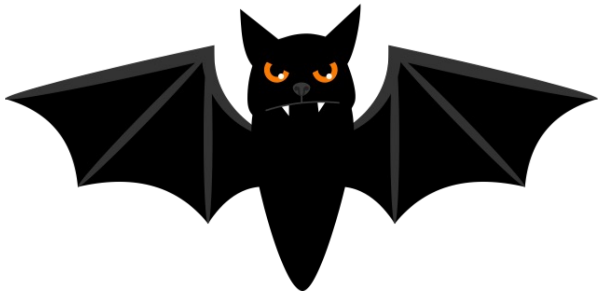
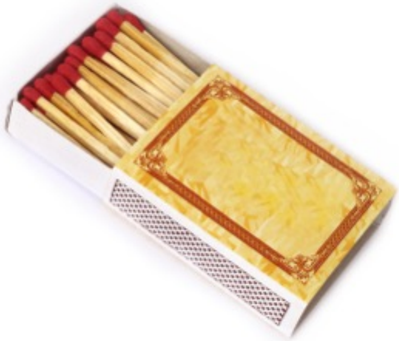
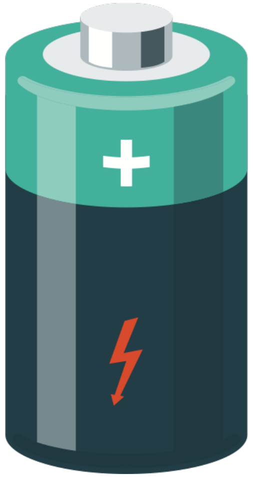
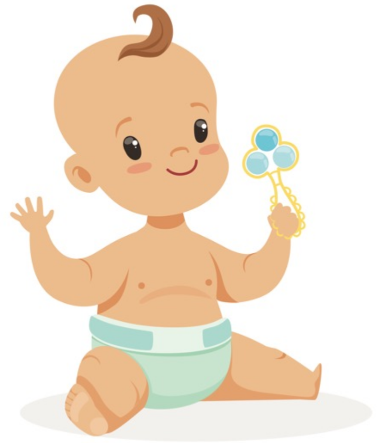

3. **Instruction:** Memorize the pictures in box A

BOX A

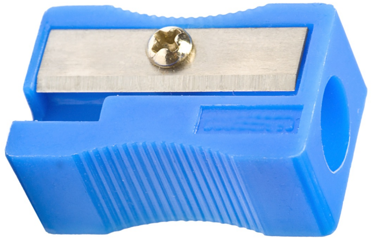

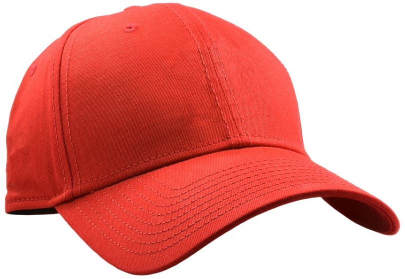

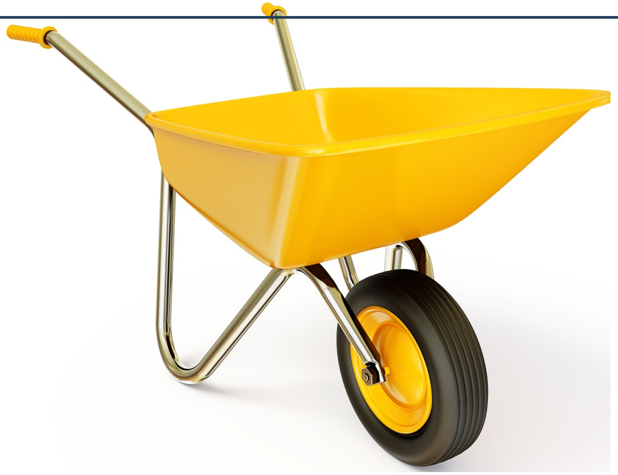

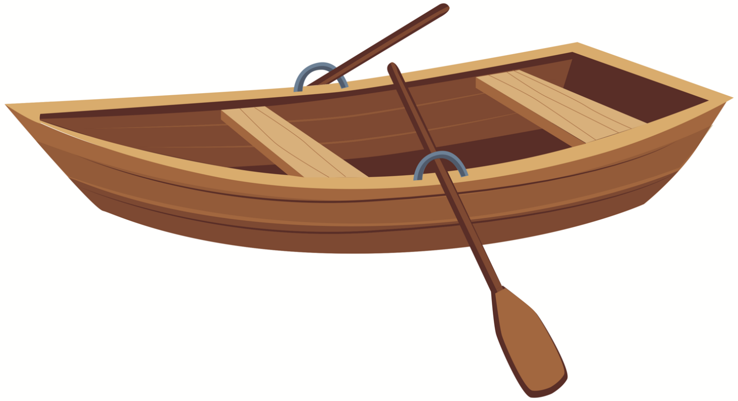

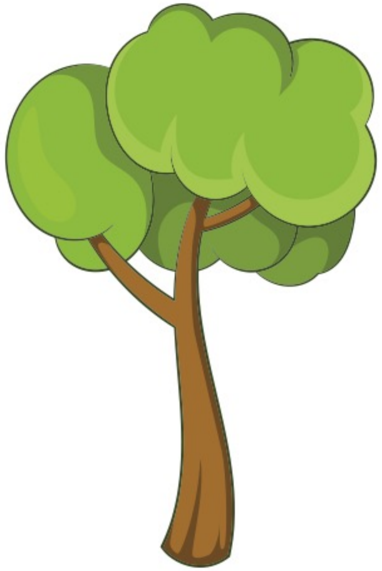

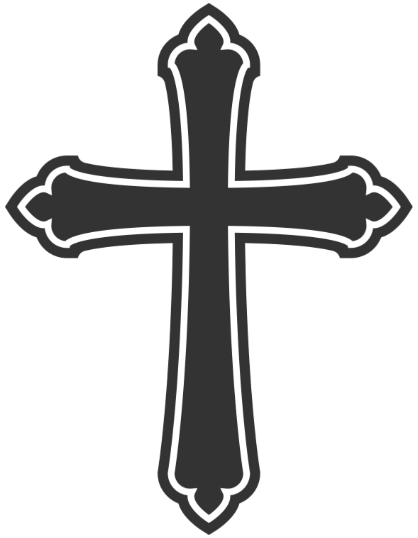

3. **Instruction:** Identify which pictures you recalled from box A and which are found in box B.

BOX B

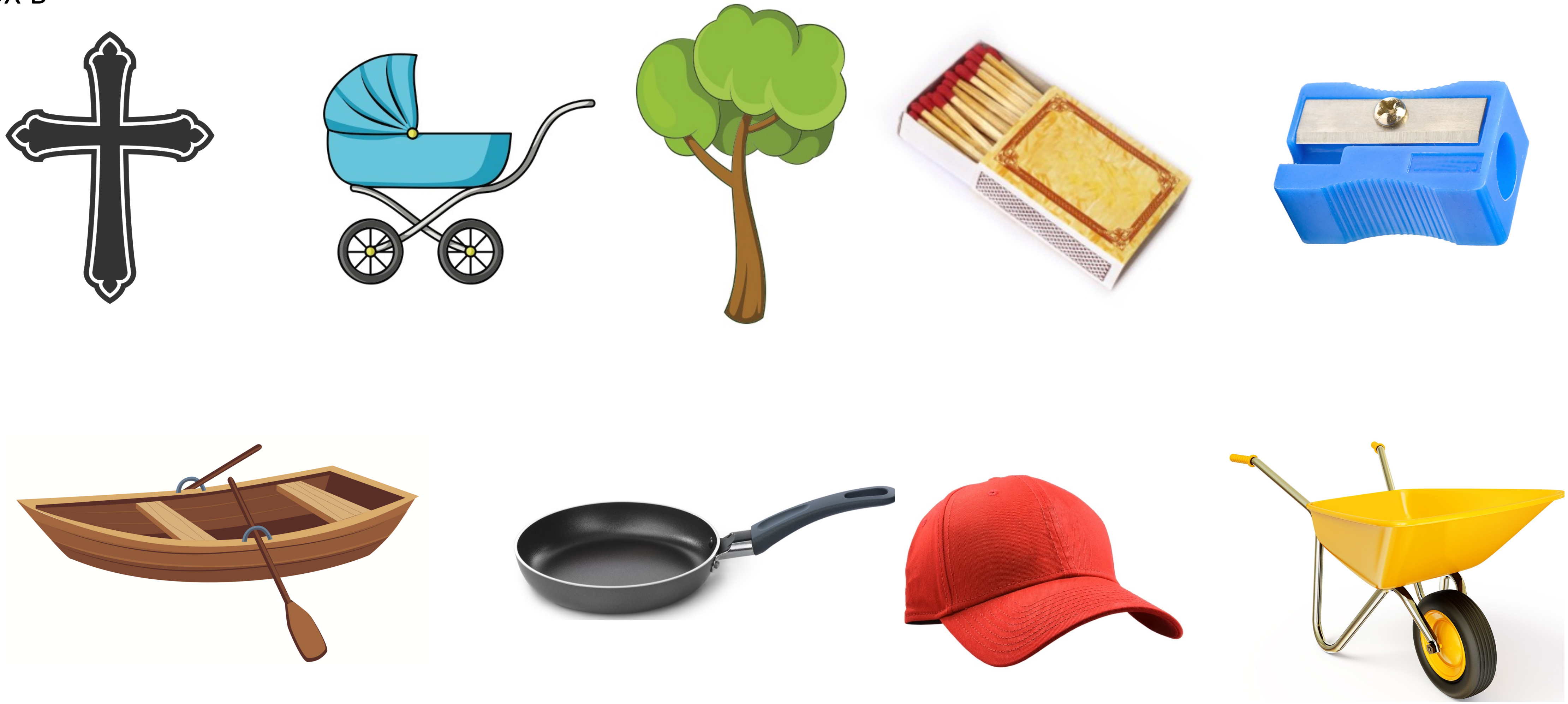

3. **Instruction:** Identify which pictures you recalled from box A and which are found in box B.

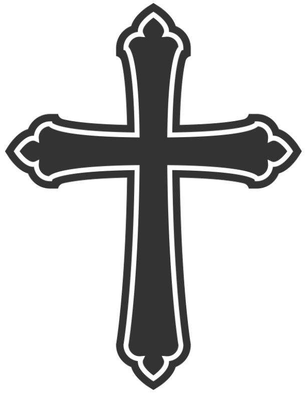
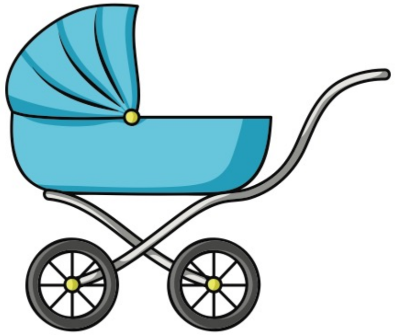
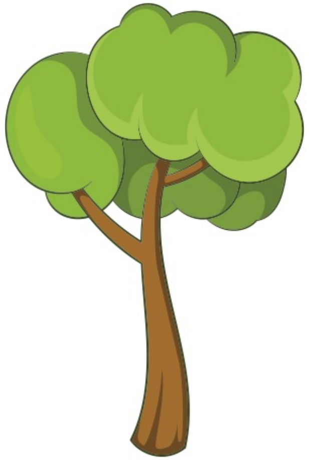
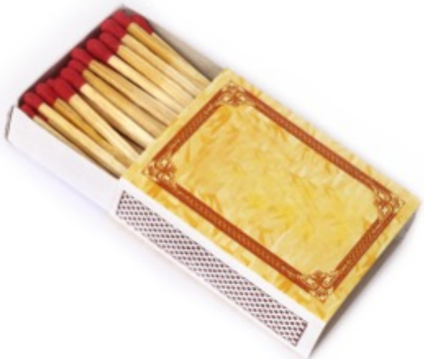
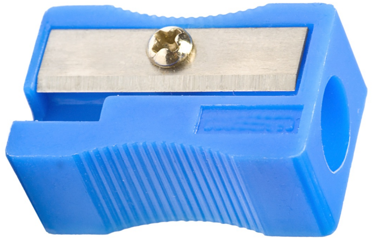

Answer: pencil sharpener, cap, pushcart/wheel barrow, boat, tree, cross

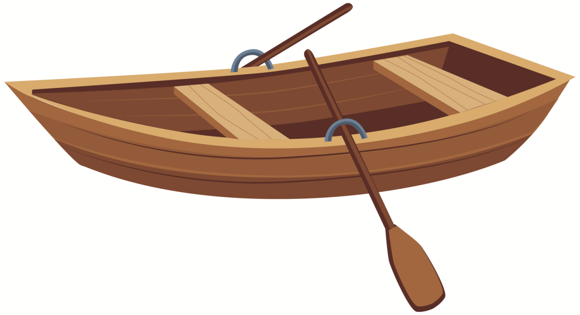
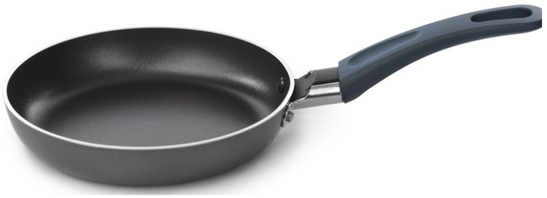
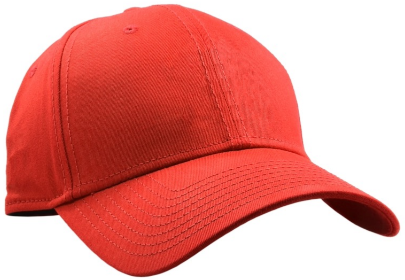
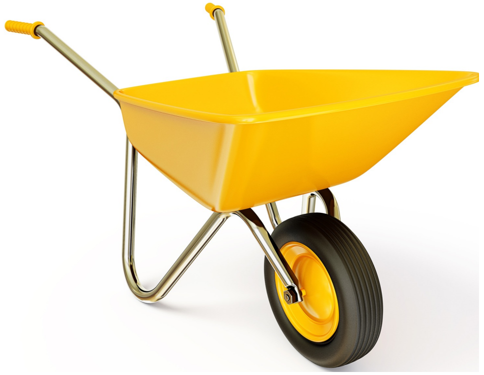

4. **Instruction:** Memorize the pictures in box A

BOX A

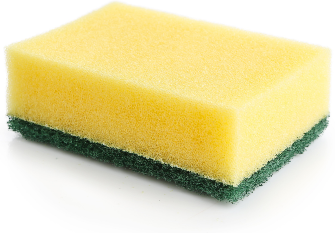
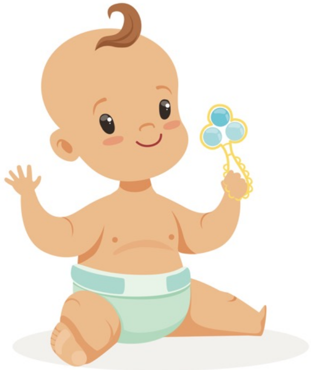
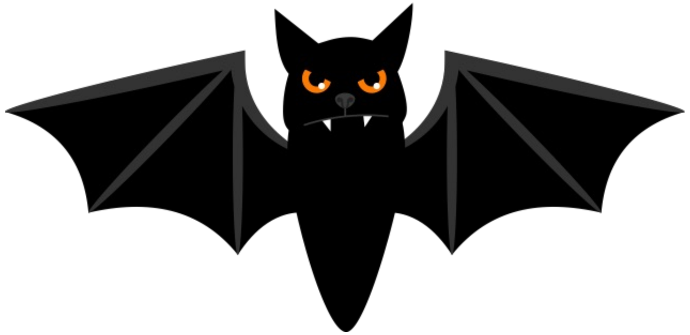
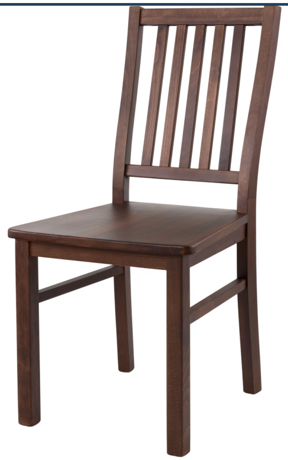
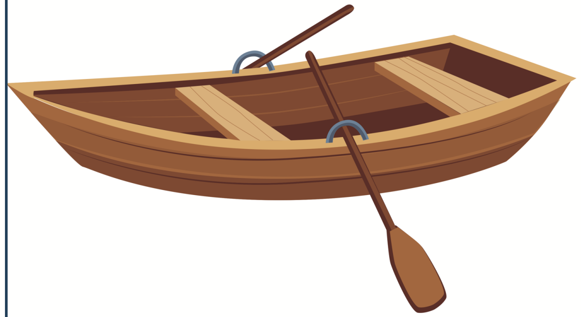
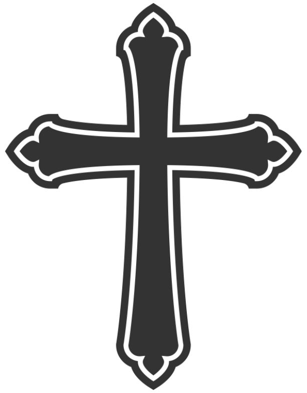
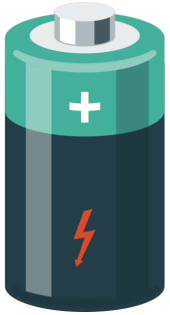
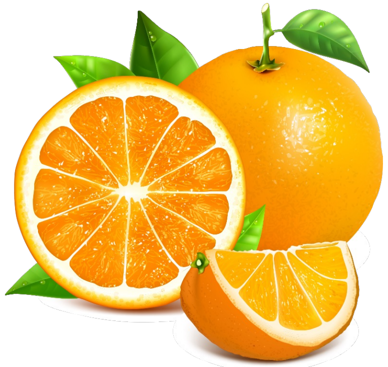

4. **Instruction:** Identify which pictures you recalled from box A and which are found in box B.

BOX B

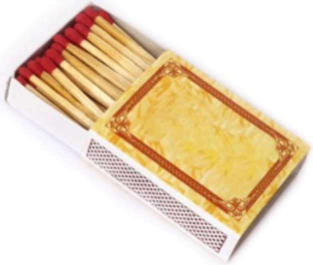
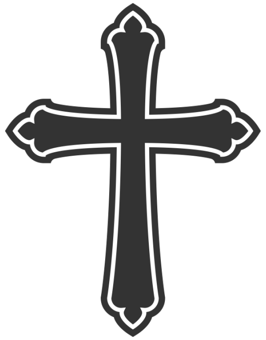
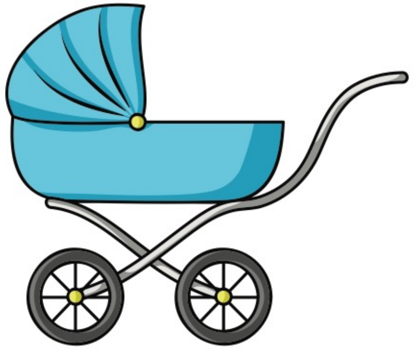
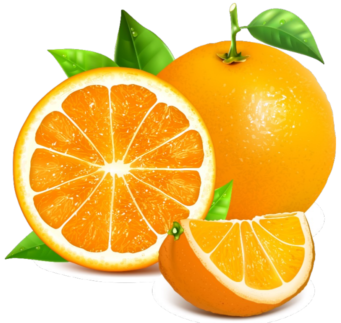
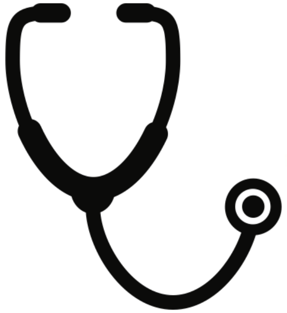
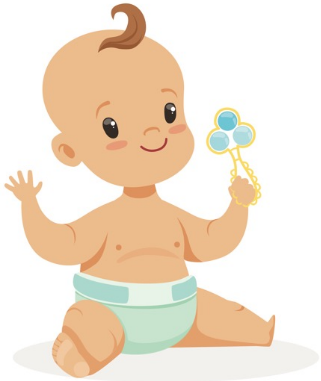
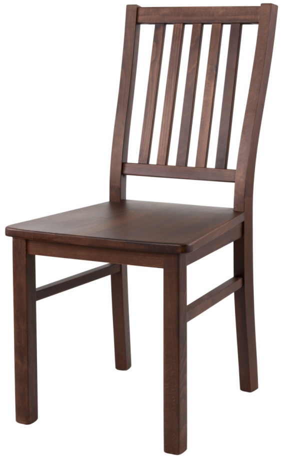
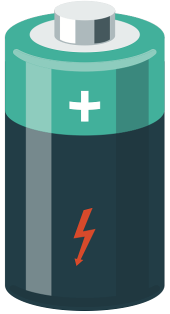
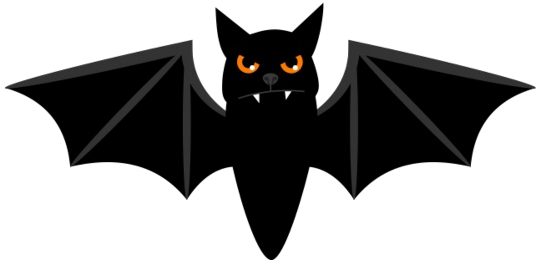
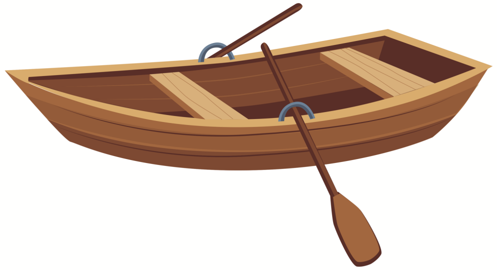
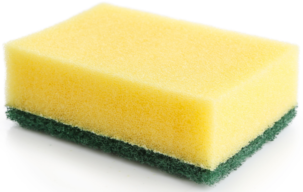

4. **Instruction:** Identify which pictures you recalled from box A and which are found in box B.

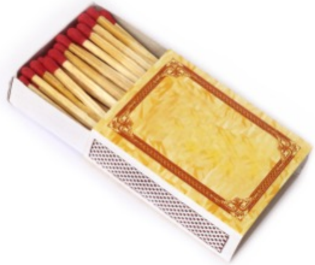
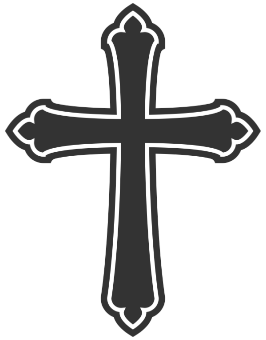
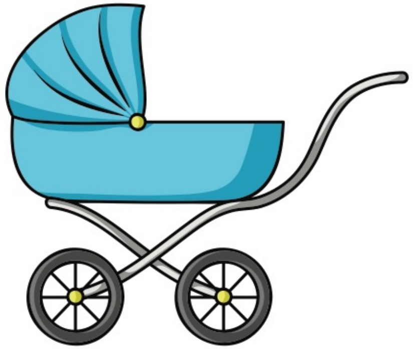
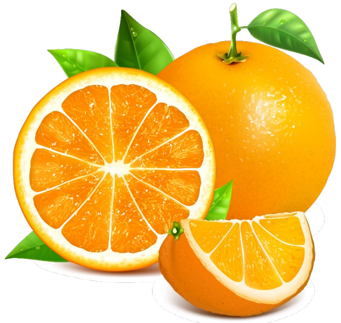
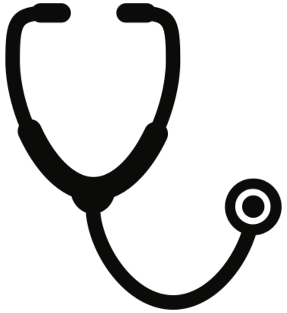
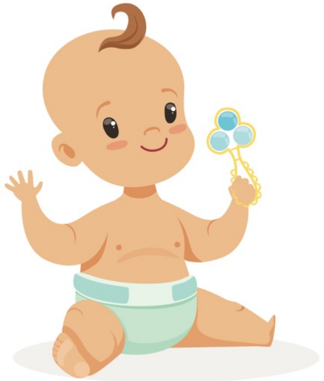

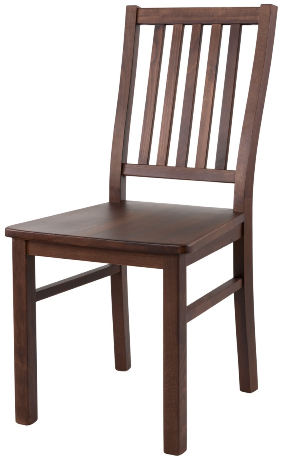

Answer: sponge, baby, bat, chair, boat, cross, battery, orange

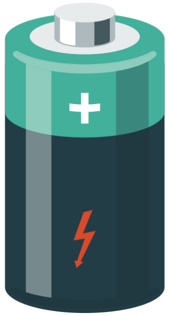
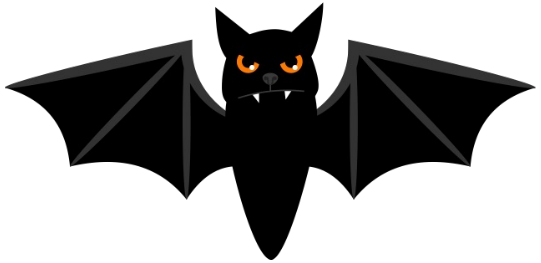
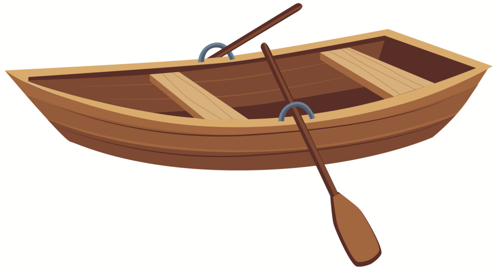
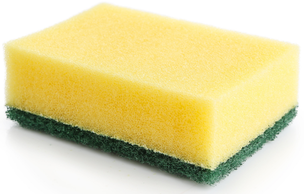

5. **Instruction:** Memorize the pictures in box A

BOX A

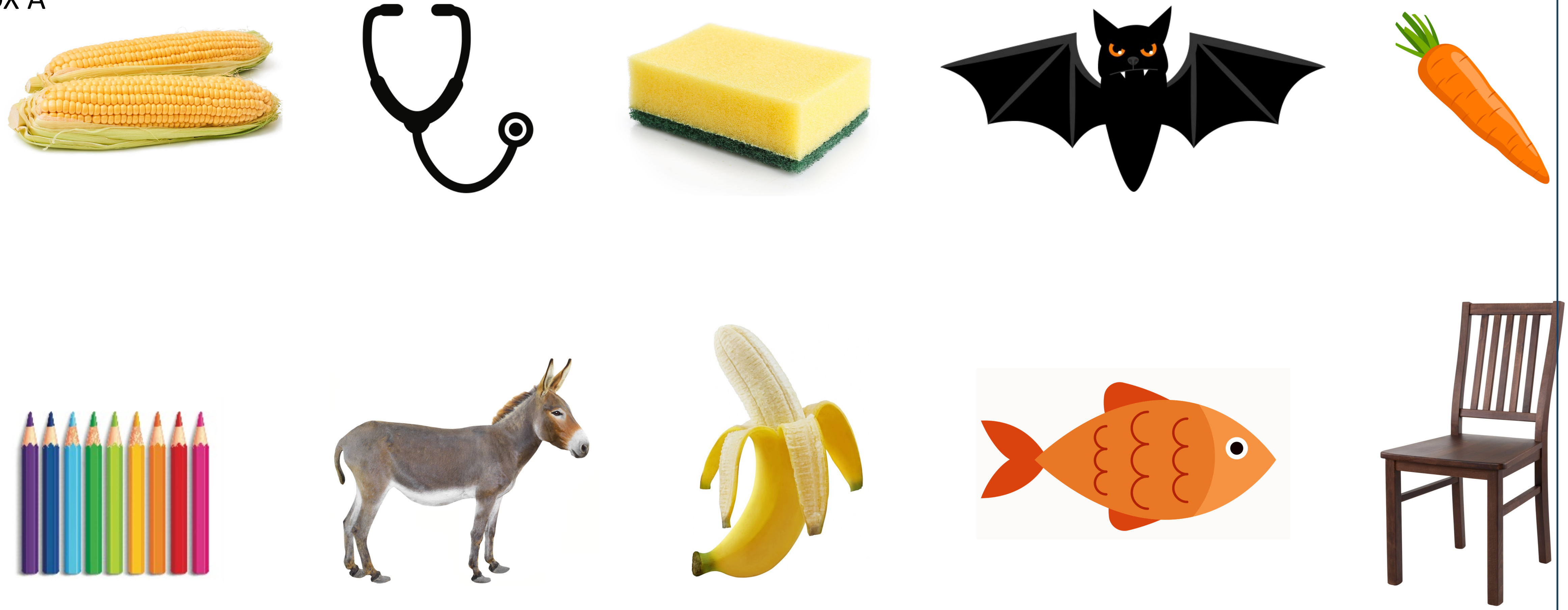

5. **Instruction:** Identify which pictures you recalled from box A and which are found in box B.

BOX B

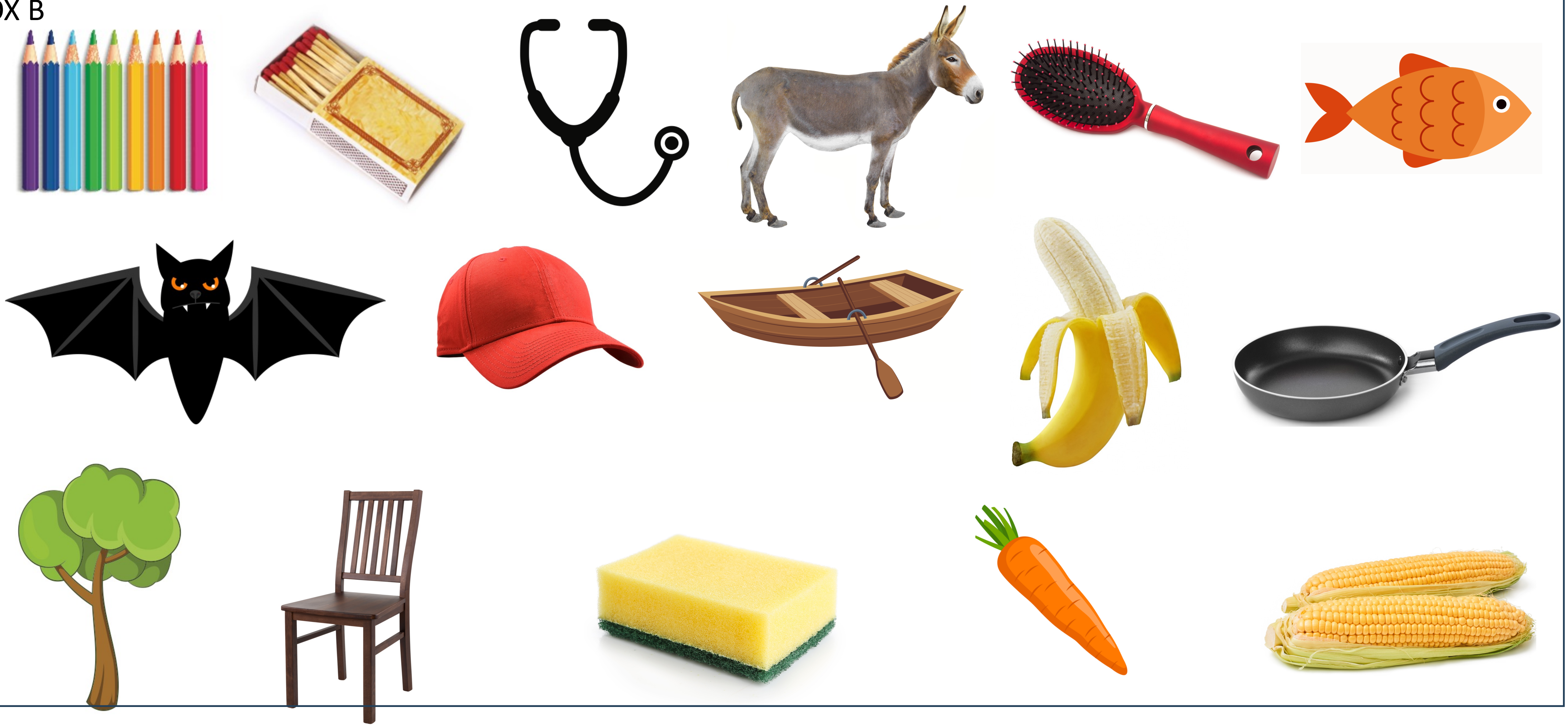

5. **Instruction:** Identify which pictures you recalled from box A and which are found in box B.

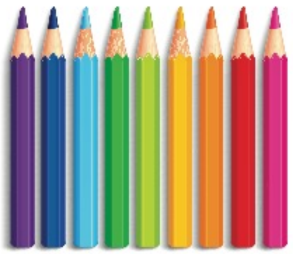
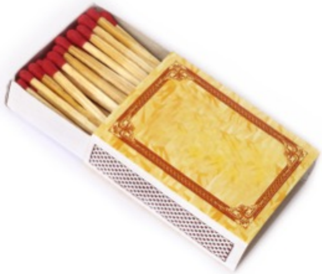
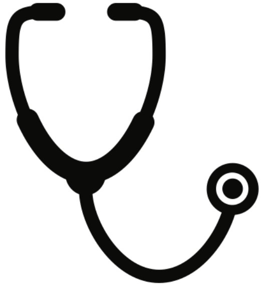
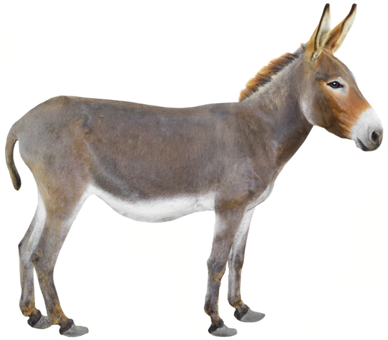
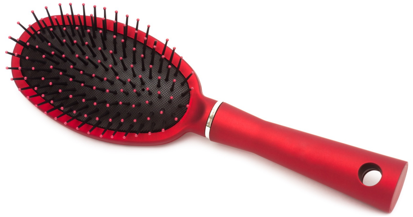
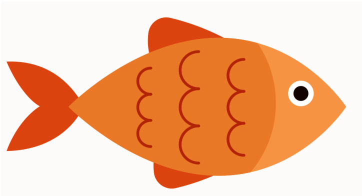
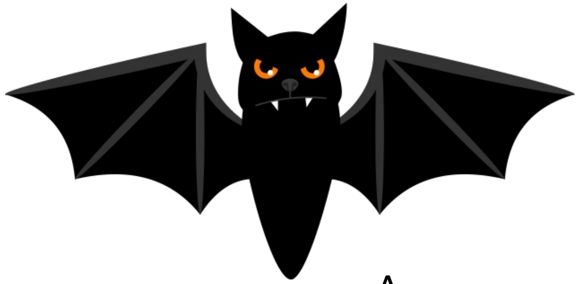
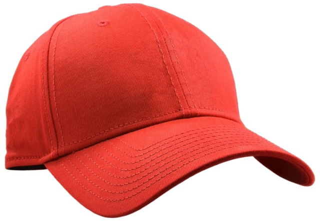
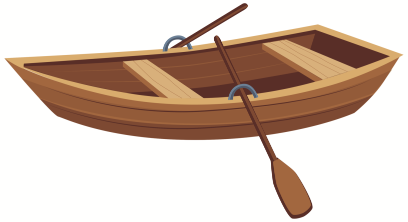
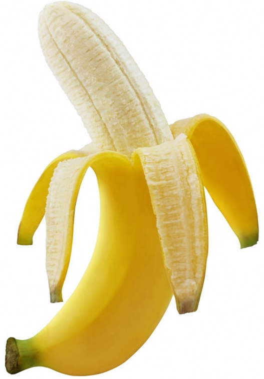
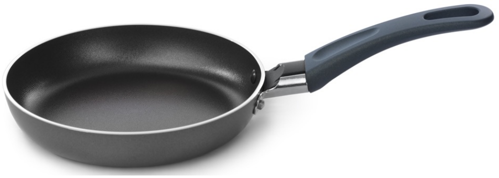

Answer: corn, stethoscope, sponge, bat, carrot, color pencil, donkey, banana, fish, chair

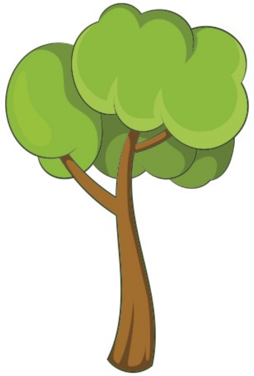
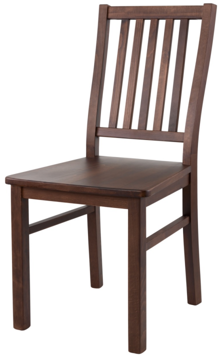
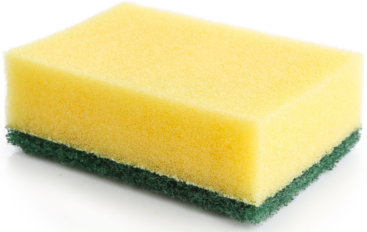
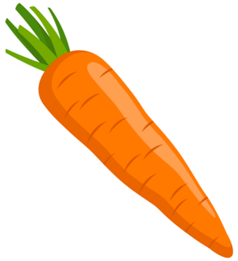
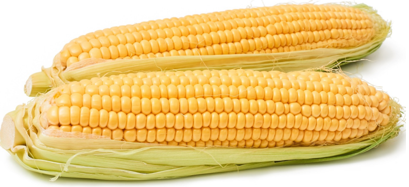

READING

Instruction: Read the following sentences

Instruction: Read the following sentences

1. She sells seashells by the sea shore
2. Fresh fried fish, fish fresh fried, fried fish fresh, fish fried fresh.
3. I scream, you scream, we all scream for ice cream!
4. I saw a kitten eating chicken in the kitchen.
5. If two witches were watching two watches, which witch would watch which watch.

Word Formation

Instruction: Form the word correctly

1. NET – PLA
2. PU – TER – COM
3. VI – TE – SI - ON – LE

Word Formation

Instruction: Form the word correctly

1. NET – PLA
2. PU – TER – COM
3. VI – TE – SI - ON – LE

Answer: planet, computer, television

4. **Instruction:** The word COLOR, just doesn't fit in:

a) in b)ful c)less

5. **Instruction:** Where could you buy a PAPER?

4. **Instruction:** The word COLOR, just doesn't fit in:

a) in b)ful c)less

Answer: a

5. **Instruction:** Where could you buy a PAPER?

Answer: stationery shop

DRAWING

Instructions: Draw the following shapes on the answer sheet

Instructions: Draw the following shapes on the answer sheet

1. Circle
2. Triangle
3. Cube

Instructions: Copy the following forms on the answer sheet

4.

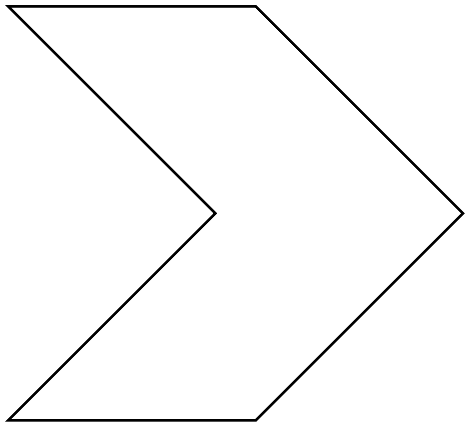

5.

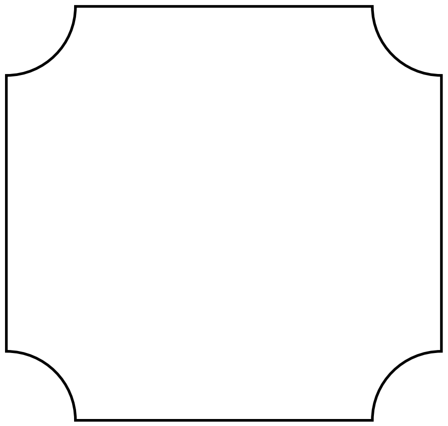

SPELLING

Instruction: Spell the words in the forward or the reverse order

Spell in the forward order

1. ARC
2. WHALE
3. SANDWICH

Spell in the reverse order

4. ADORE
5. CONTRACT
