# Supplementary material for: Filling the gap: brief neuropsychological assessment protocol for glioma patients undergoing awake surgeries
Source: Front Psychol. 2024 Aug 9;15:1417947. doi: 10.3389/fpsyg.2024.1417947 (PMC11342098; doi:10.3389/fpsyg.2024.1417947)
Supplement: Supplementary file 6 [file Data_Sheet_6.PDF]

OMFTCT
Ohy-Maldaun Fast Track Cognitive Test

AWAKE SURGERY– *POST OPERATIVE - After 30 days*

NAMING

Instructions: Say the name of this figure

1. **Instructions:** Say the name of this figure

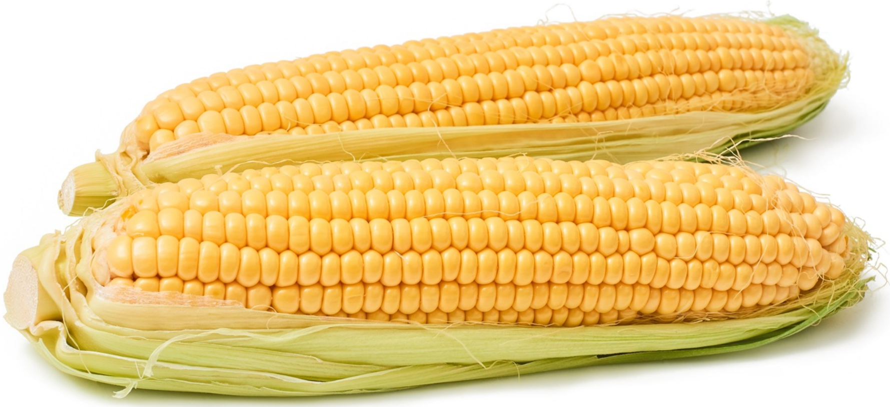

Answer: steering wheel

2. **Instructions:** Say the name of this figure

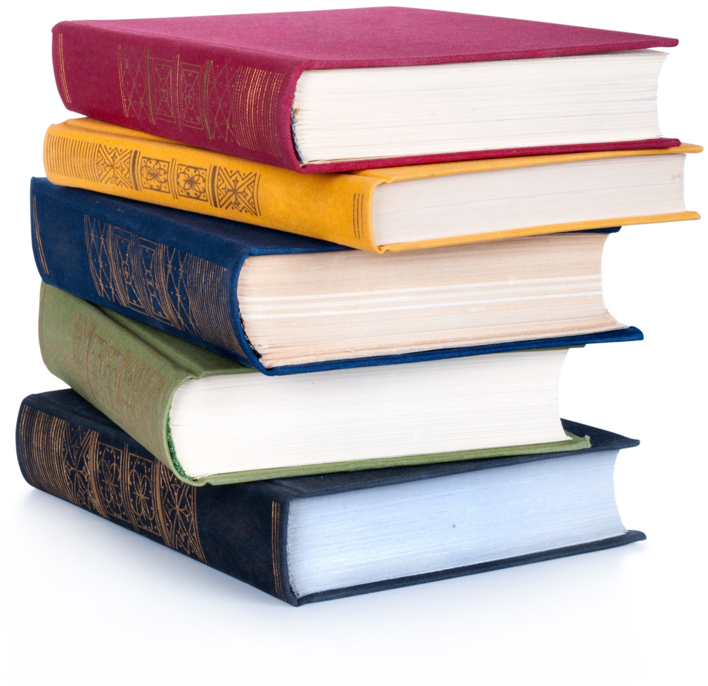

Answer: books

3. **Instructions:** Say the name of this figure

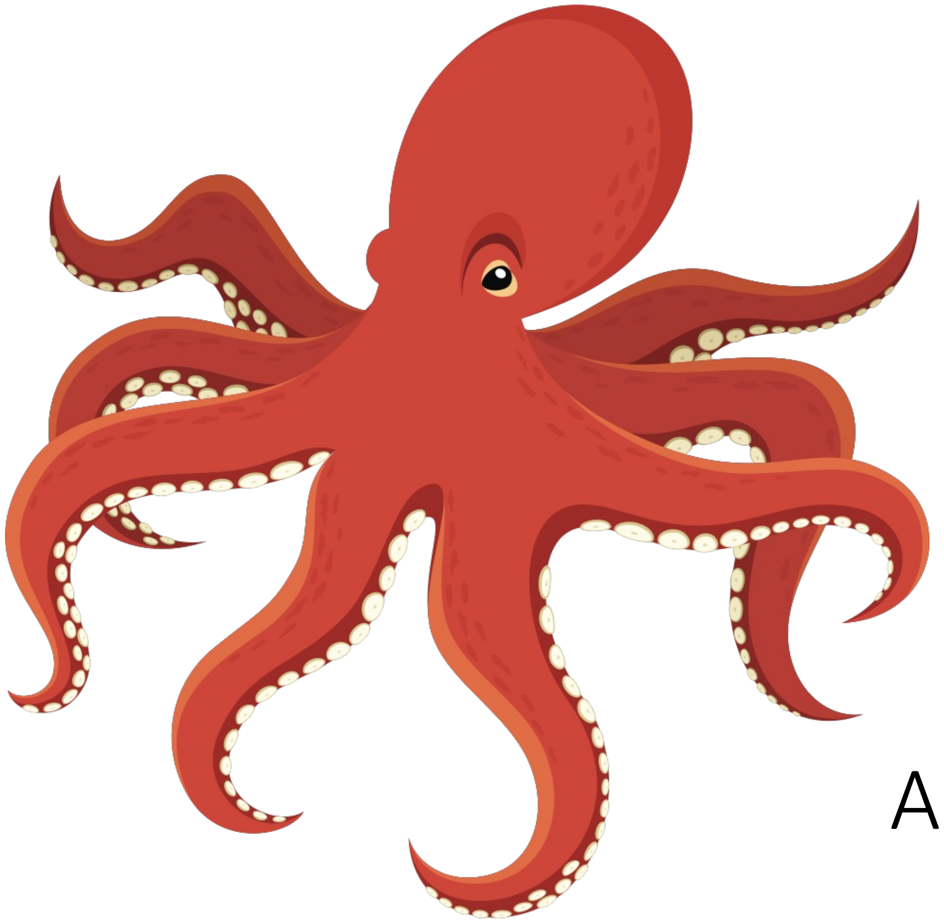

Answer: octopus

Dual task naming
(Open and close the hands, alternately while naming the figure)

4. **Instructions:** Say the name of this figure while making the movement with your hands

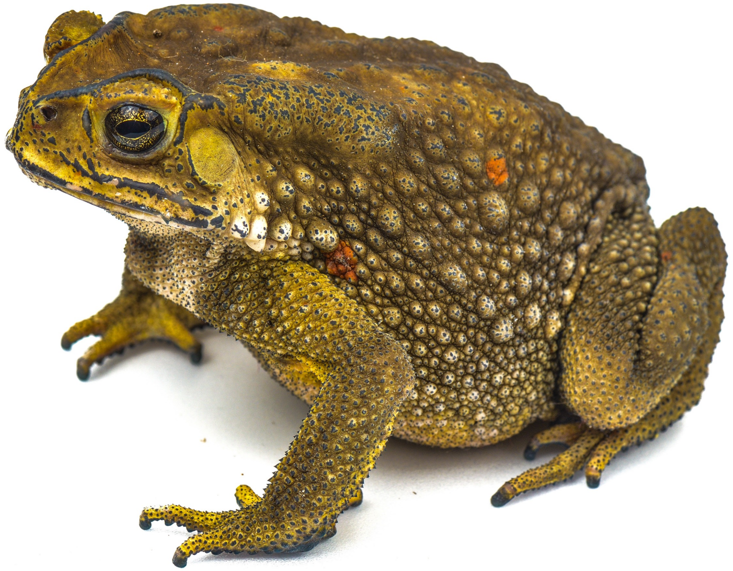

Answer: frog

5. **Instructions:** Say the name of this figure while making the movement with your hands

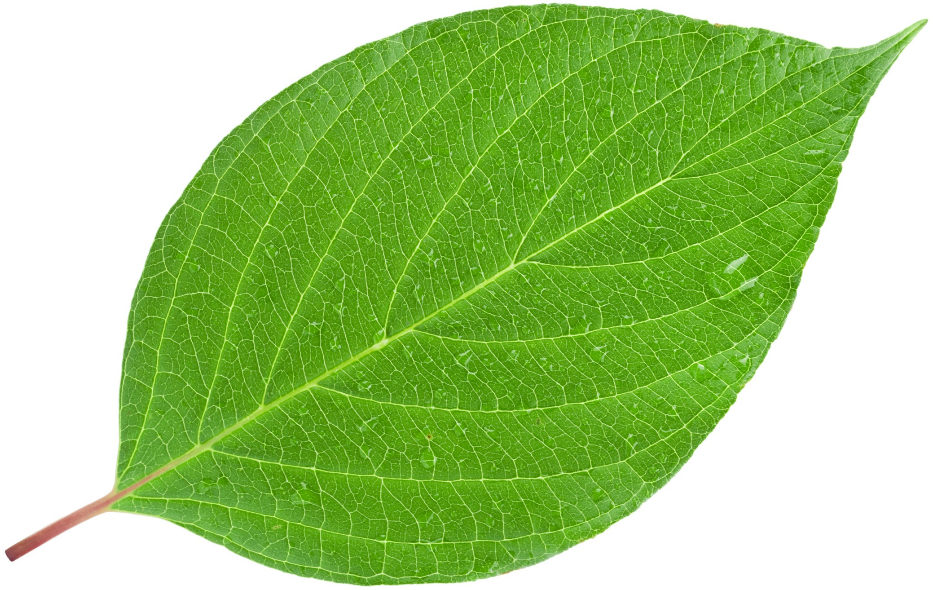

Answer: leaf

VERBAL MEMORY

Instructions: Memorize and repeat what is said

VERBAL MEMORY

Instructions: Memorize and repeat what is said:

1. Gray is the color of the rat that entered the yellow house.
2. The gray rat entered the yellow house that had a door.
3. Shoes – swallow – plum – glove
4. 4 – 0 – 1 – 9 – 7
5. Q – J – D – I – V – O

SEMANTIC

Instructions: Say which figure at the bottom relates to the main stimulus at the top.

1. **Instructions:** Say which figure at the bottom relates to the main stimulus at the top.

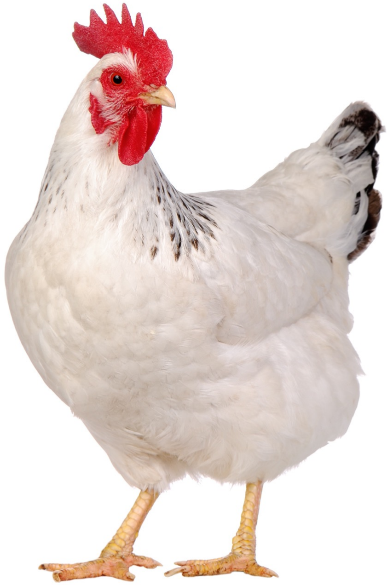

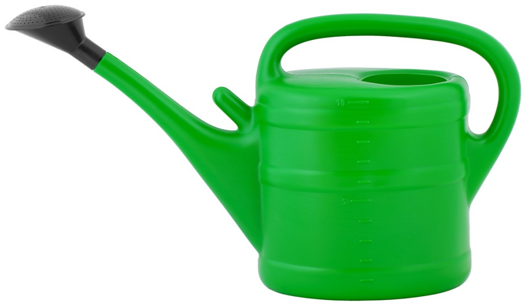

Answer: egg

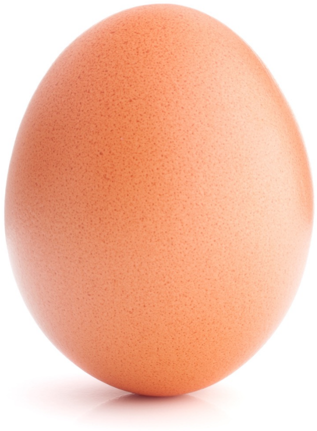

2. **Instructions:** Say which figure at the bottom relates to the main stimulus at the top.

fireman

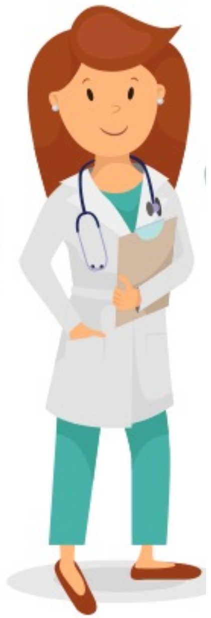

Answer: house on fire

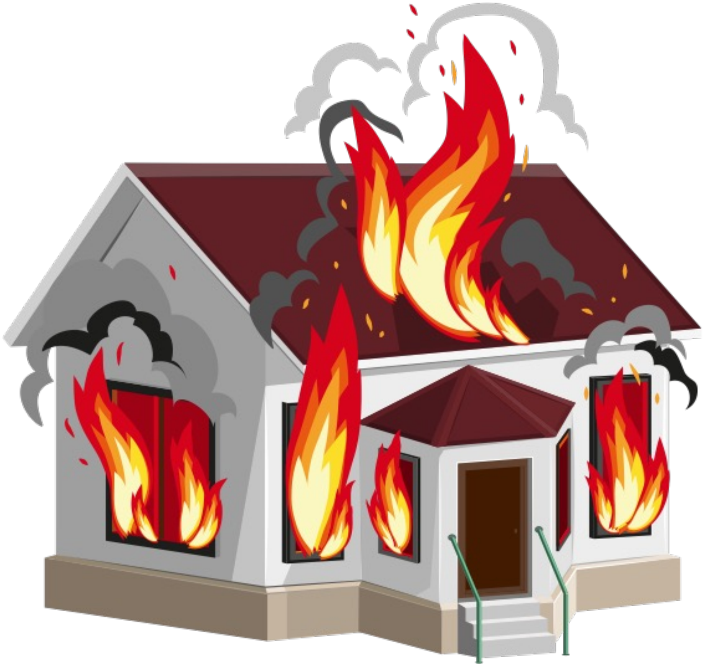

Dual task semantics
(Touch the right ear with the left hand, alternately, while answering the exercise)

3. **Instructions:** Answer the questions while moving your hands.

What is it for?

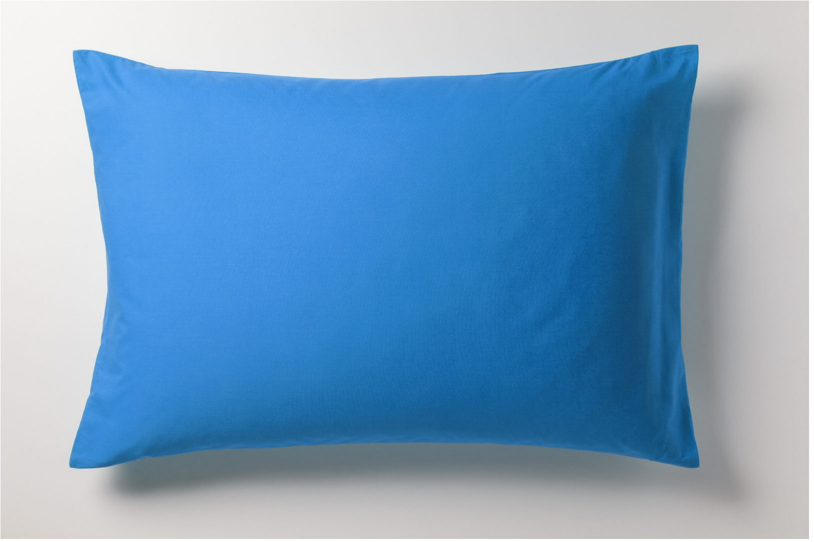

Answer: lie down / rest / sleep

4. **Instructions:** Answer the questions while moving your hands.

What material is it made of?

KEY

Answer: metal

5. **Instructions:** Answer the questions while moving your hands.

What shape does it have?

STRAW

Answer: cilinder

CALCULATION

Instructions: Calculate and tell the result

Instructions: Calculate and tell the result

1. $7 + 4 = 11$

2. $15 – 8 = 7$

3. $23 \times 2 = 46$

4. **Instructions:** Add ONLY the non-repeating numbers and subtract 3 from the final result.

1 – 7 – 3 – 2 – 1 – 8

Answer: 17

5. **Instructions:** Subtract 3 from the number 20 and continue subtracting 3 from the results found 4 more times in a row.

Answer: 17, 14, 11, 8

WRITING

Intructions: Copy and write the words and sentences

Instructions: Copy the words:

1. Analogy
2. Career

Instructions: Copy the sentence:

3. He carried a ton of oranges in his large bag.

Instructions: Write the dictated word

4. Maintenance

Instructions: Write the dictated sentence

5. There was no alternative but to give it as a gift.

VISUAL MEMORY

Instructions: Look at the pictures in box A and then say which images from box B appeared in box A.

1. **Instruction:** Memorize the pictures in box A

BOX A

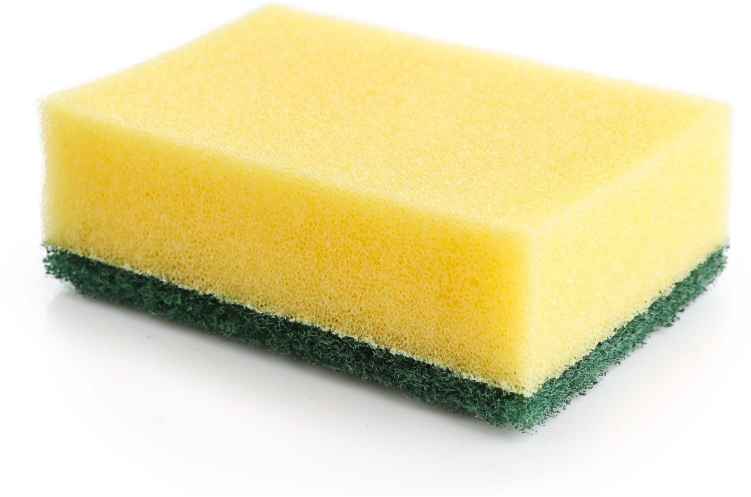

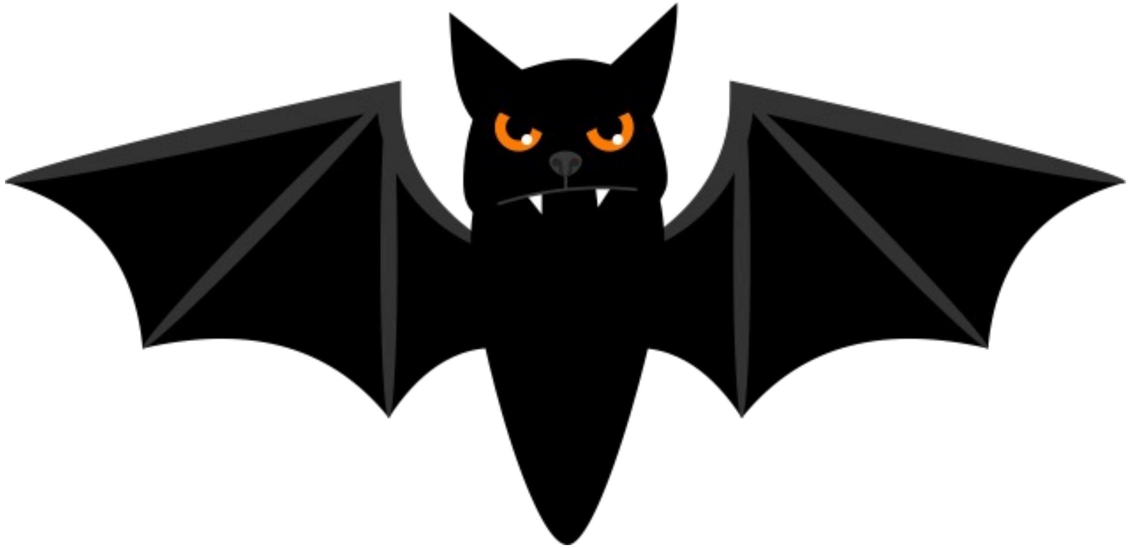

1. **Instruction:** Identify which pictures you recalled from box A and which are found in box B.

BOX B

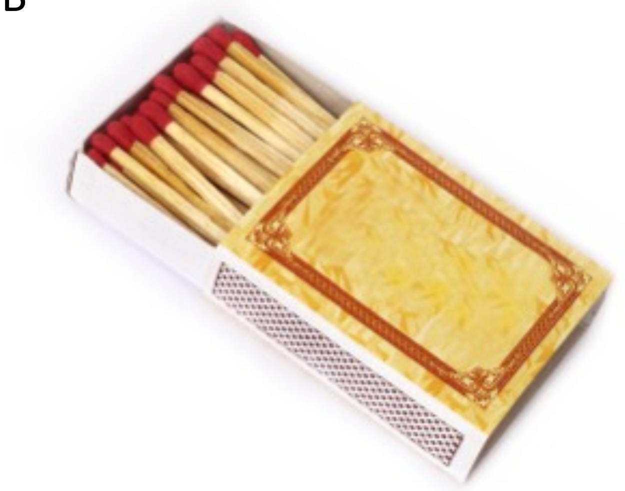

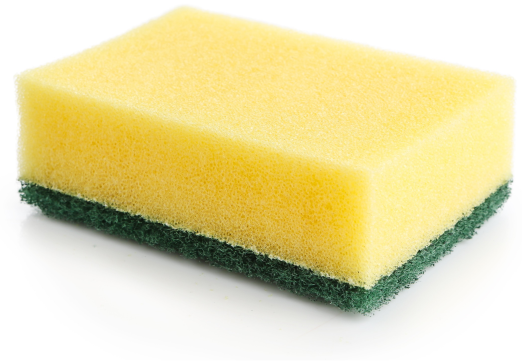

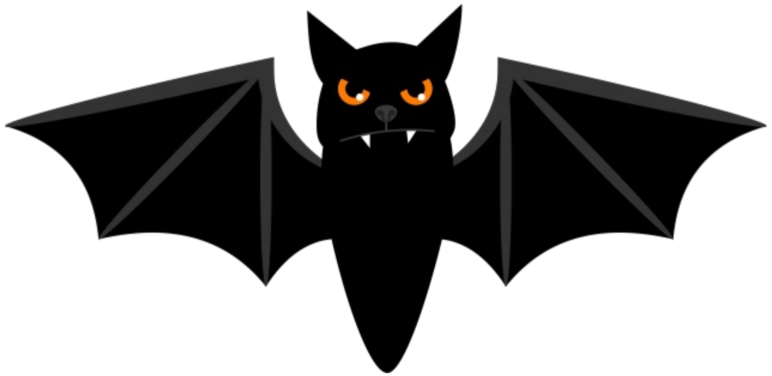

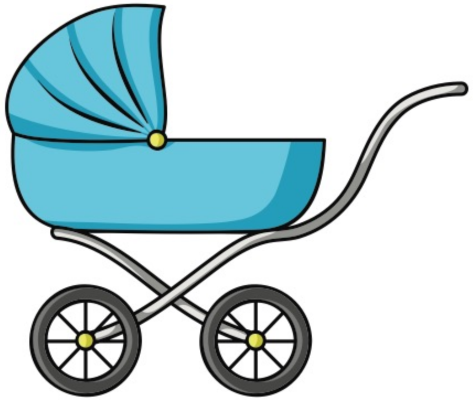

1. **Instruction:** Identify which pictures you recalled from box A and which are found in box B.

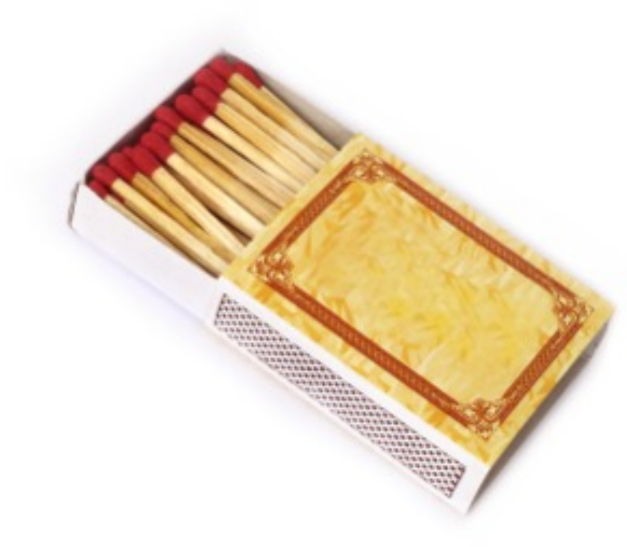

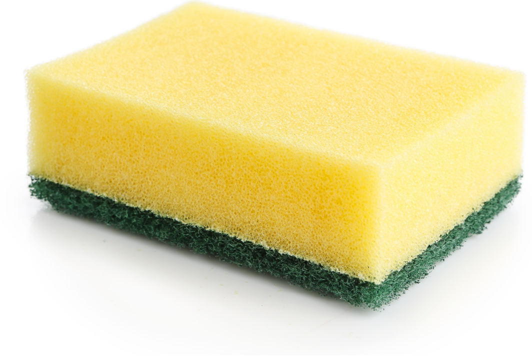

Answer: bat and sponge

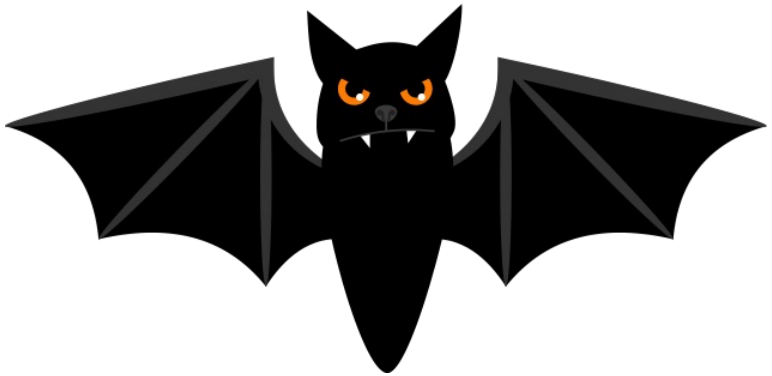

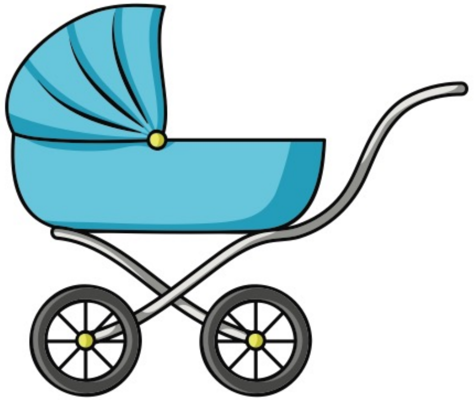

2. **Instruction:** Memorize the pictures in box A

BOX A

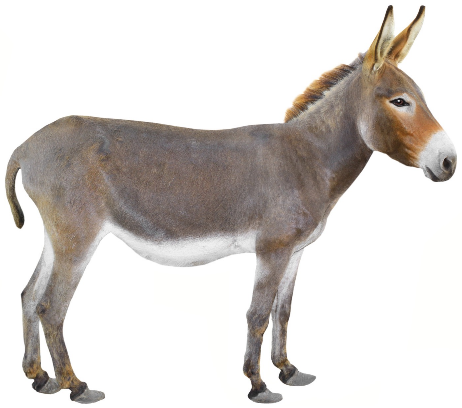

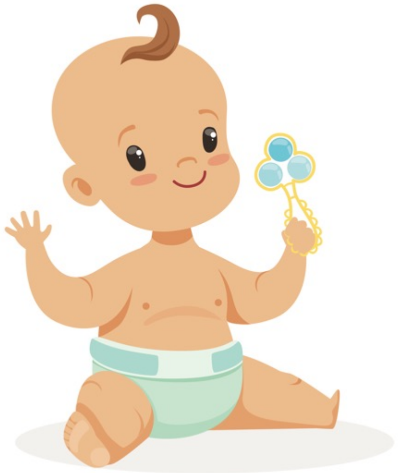

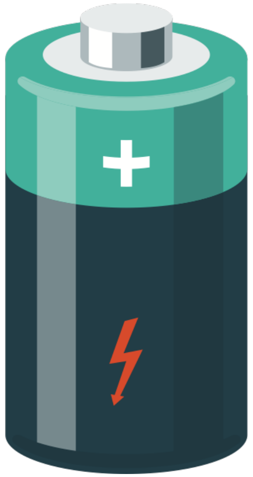

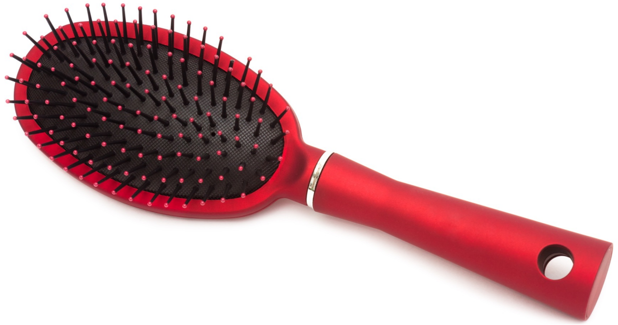

2. **Instruction:** Identify which pictures you recalled from box A and which are found in box B.

BOX B

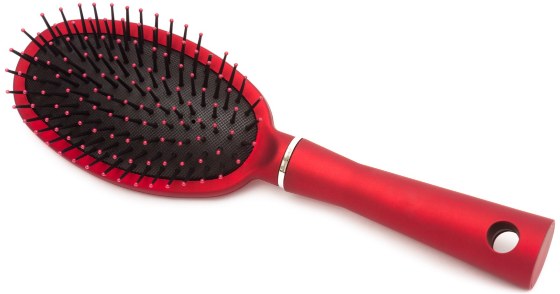
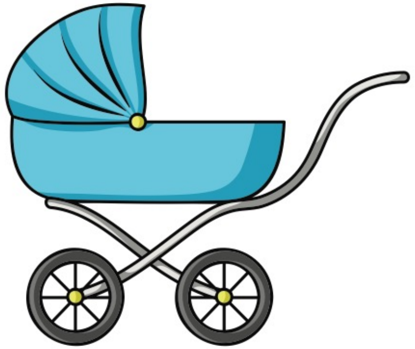
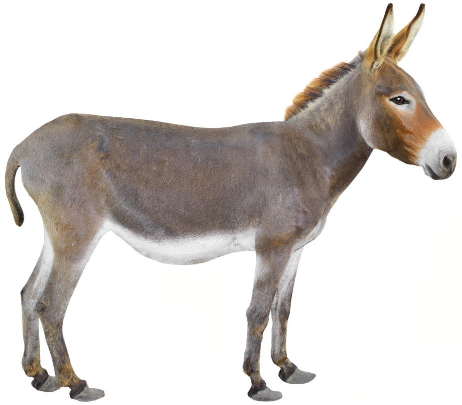
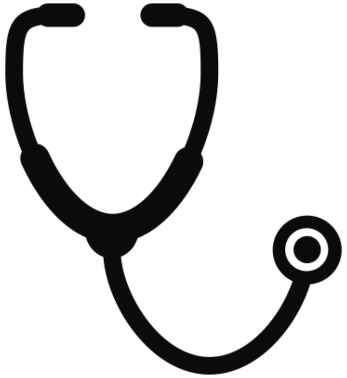
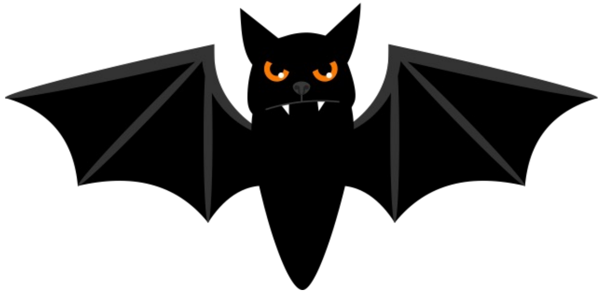
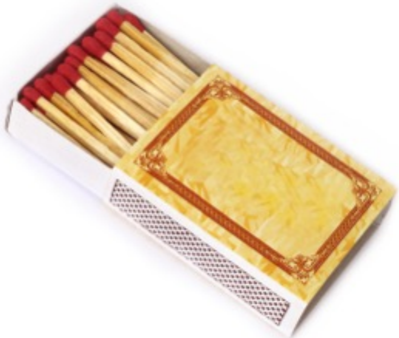
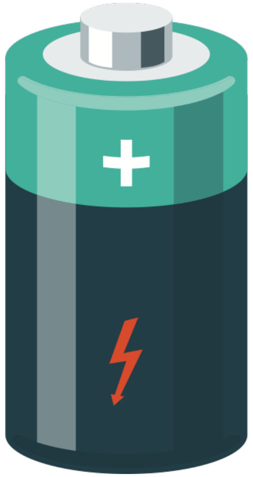
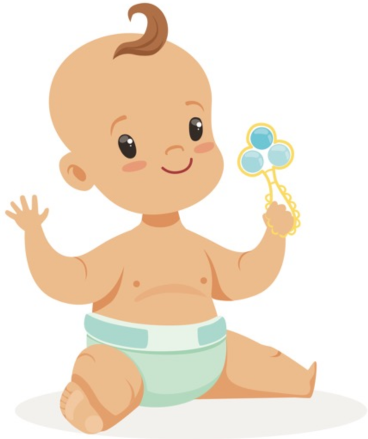

2. **Instruction:** Identify which pictures you recalled from box A and which are found in box B.

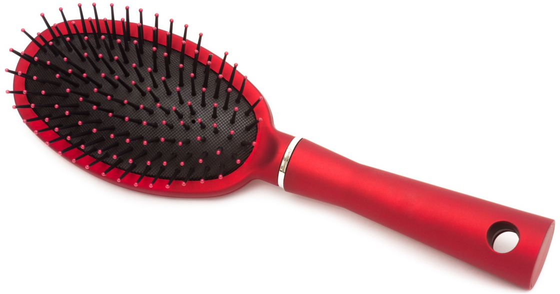
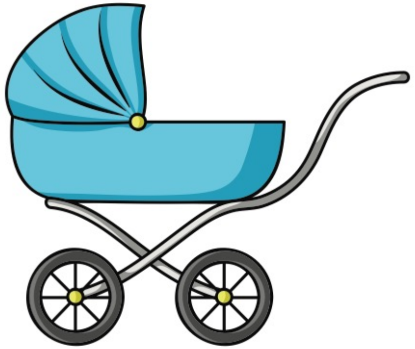
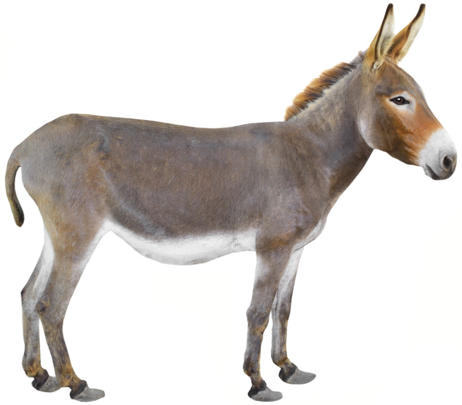
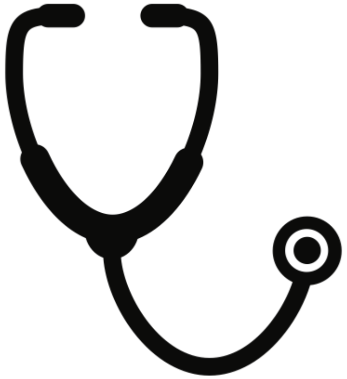

Answer: donkey, baby, battery, brush

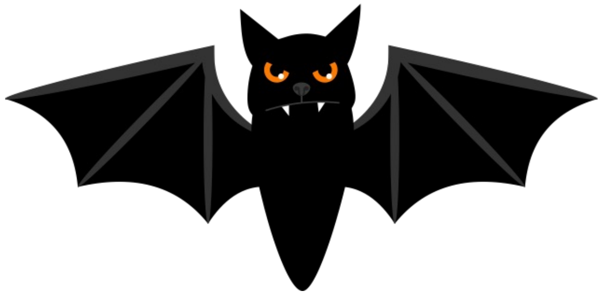
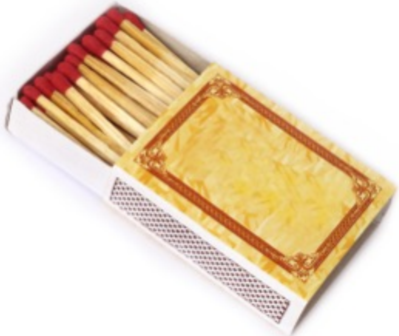
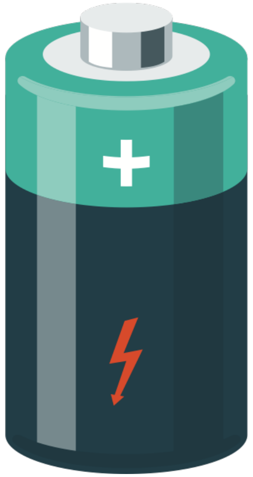

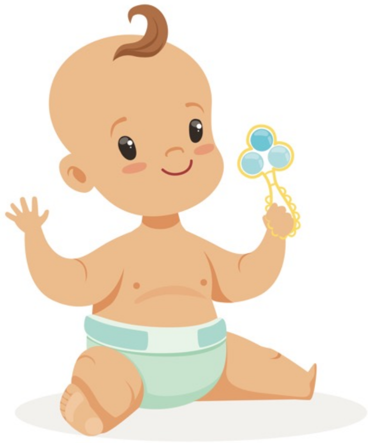

3. **Instruction:** Memorize the pictures in box A

BOX A

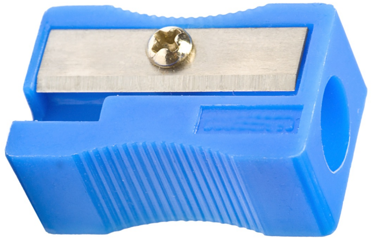
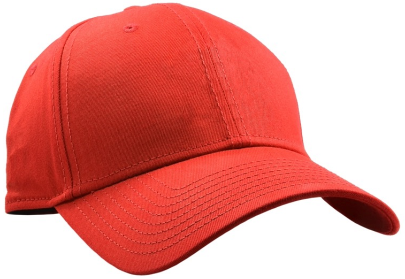
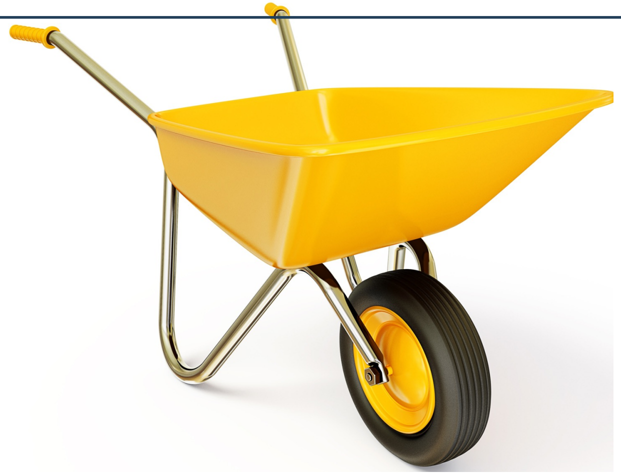
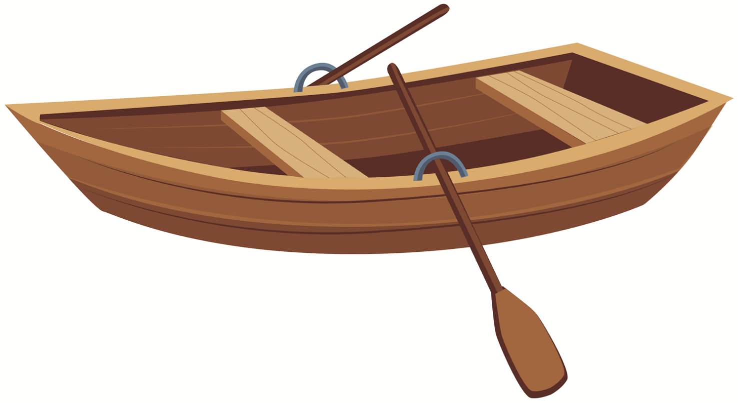
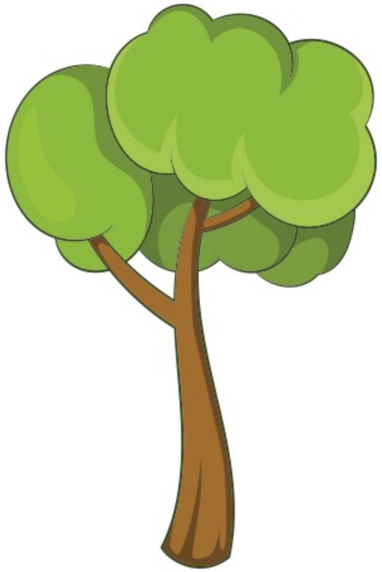
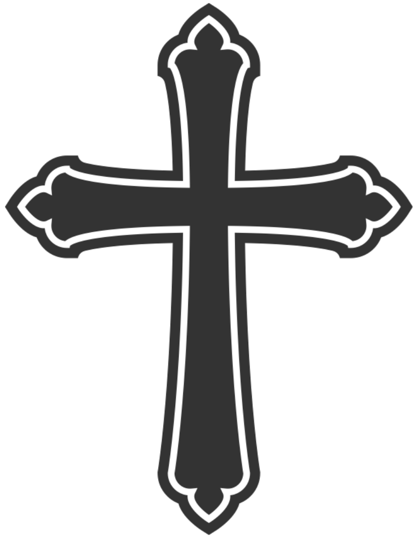

3. **Instruction:** Identify which pictures you recalled from box A and which are found in box B.

BOX B

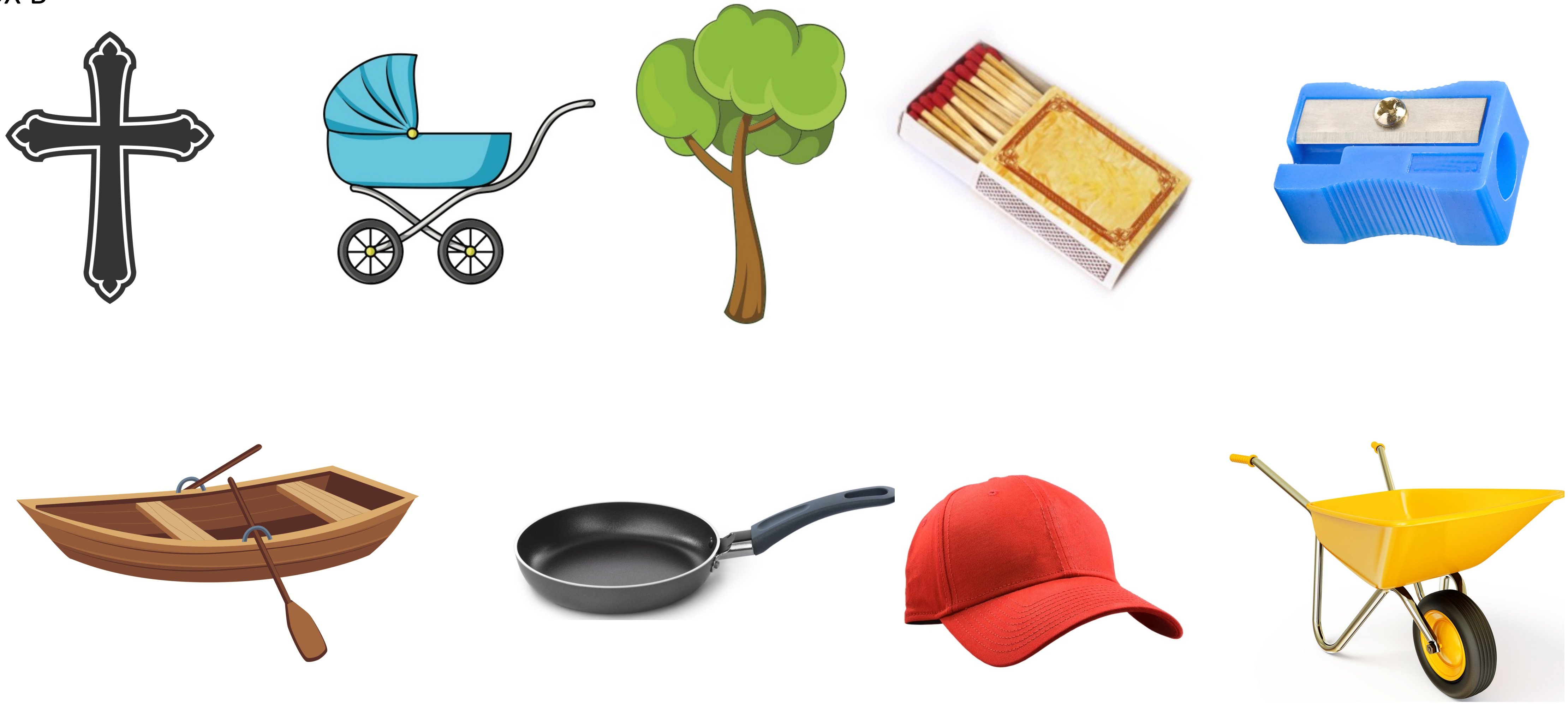

3. **Instruction:** Identify which pictures you recalled from box A and which are found in box B.

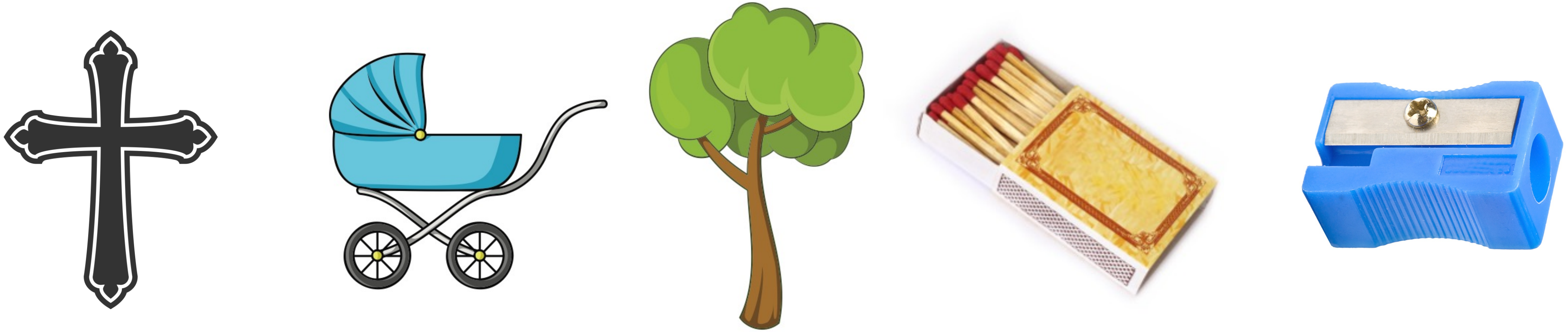

Answer: pencil sharpener, cap, pushcart/wheel barrow, boat, tree, cross

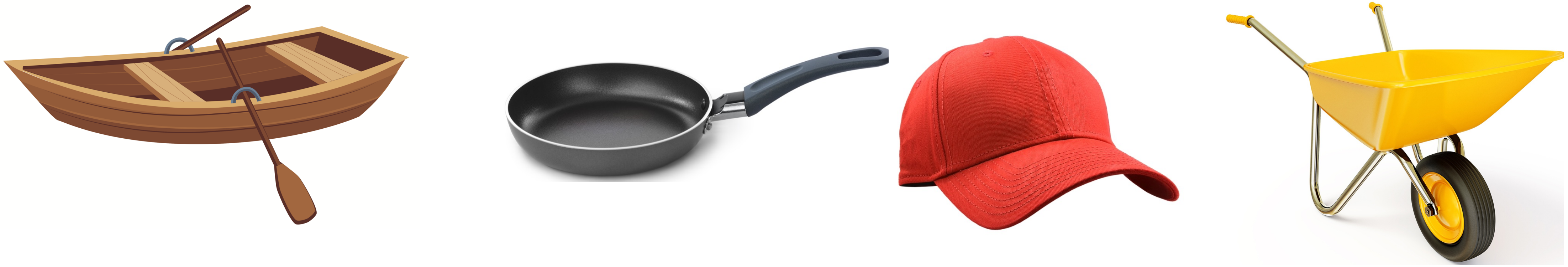

4. **Instruction:** Memorize the pictures in box A

BOX A

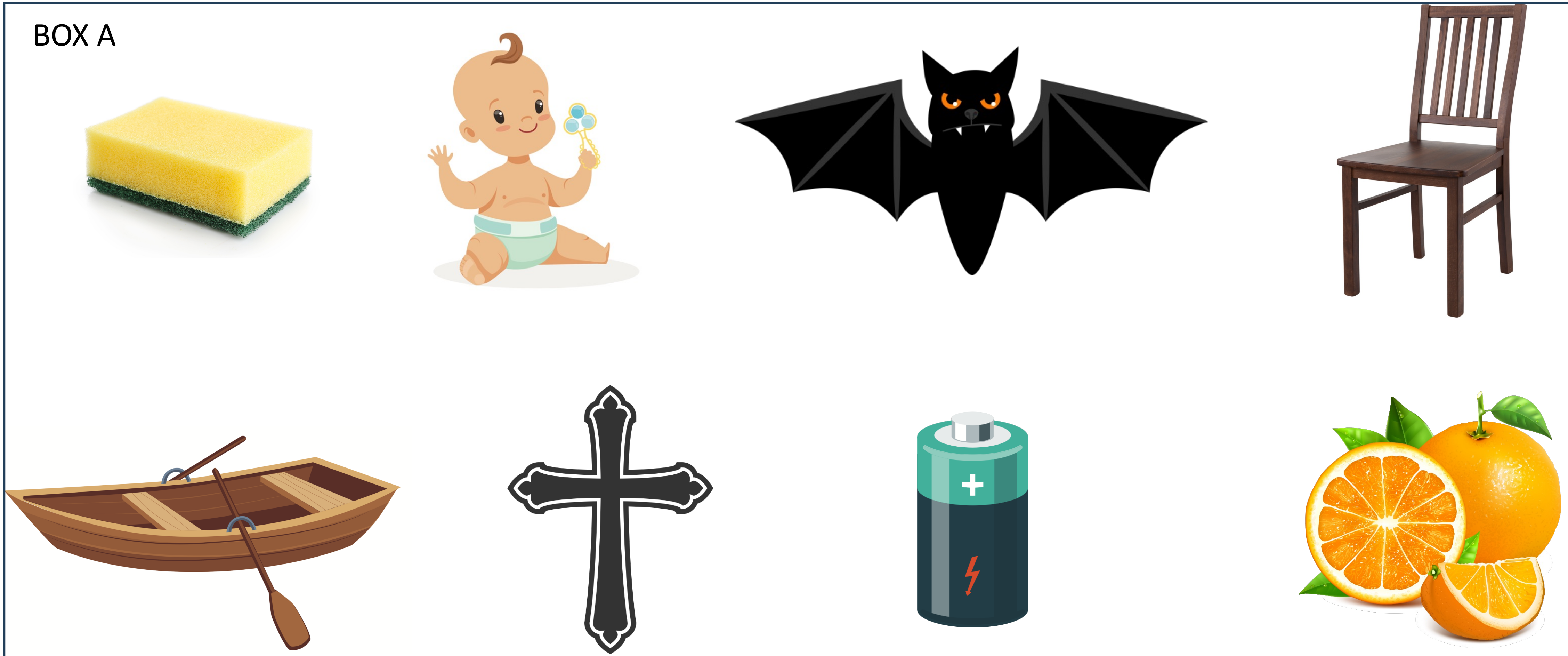

4. **Instruction:** Identify which pictures you recalled from box A and which are found in box B.

BOX B

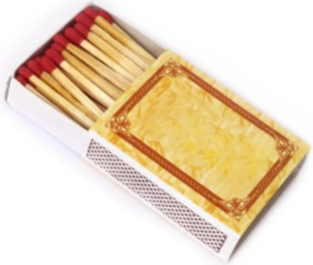
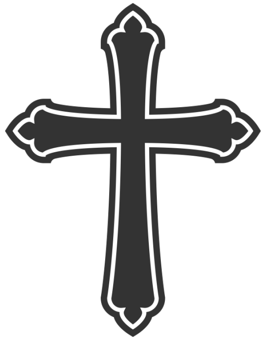
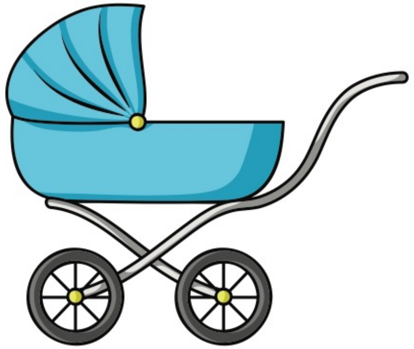
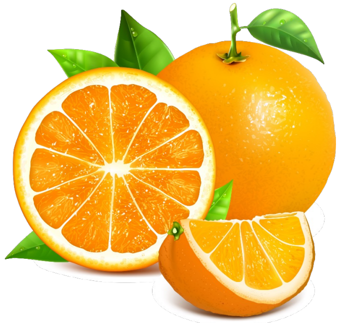
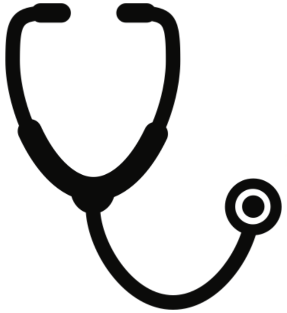
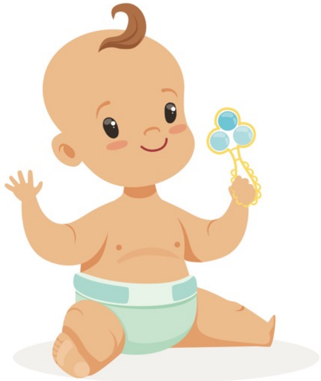
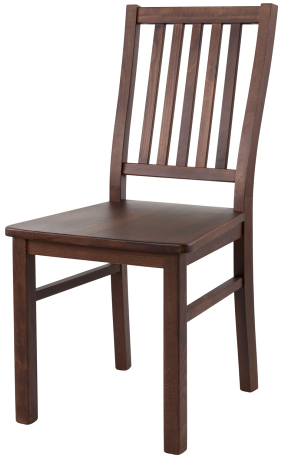
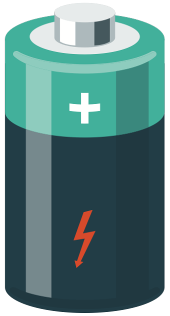
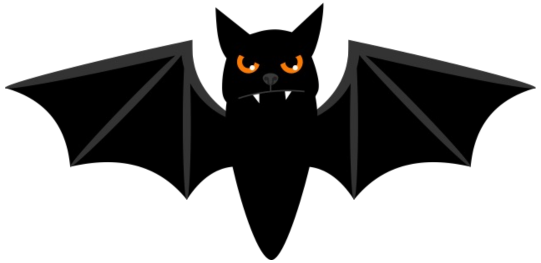
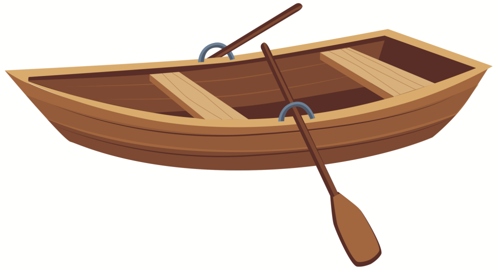
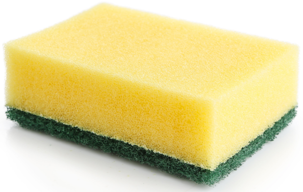

4. **Instruction:** Identify which pictures you recalled from box A and which are found in box B.

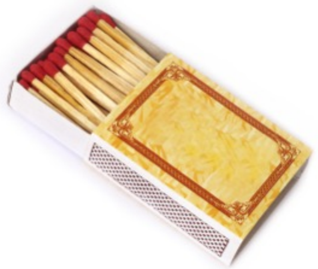
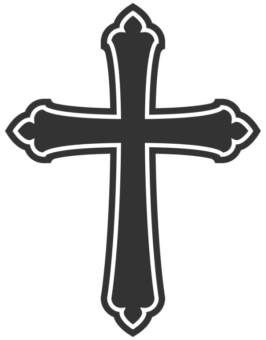
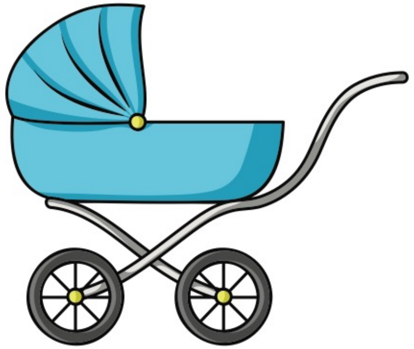
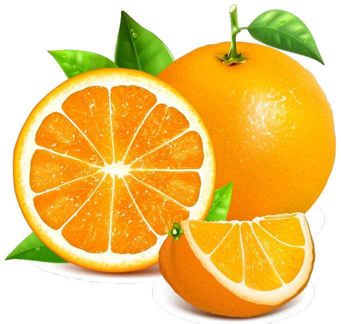
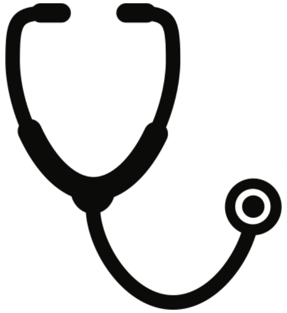
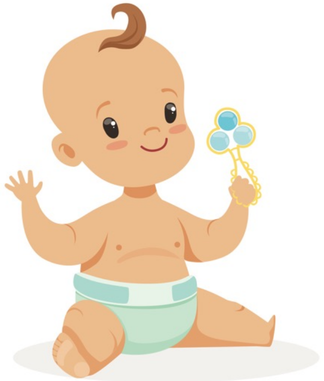

Answer: sponge, baby, bat, chair, boat, cross, battery, orange

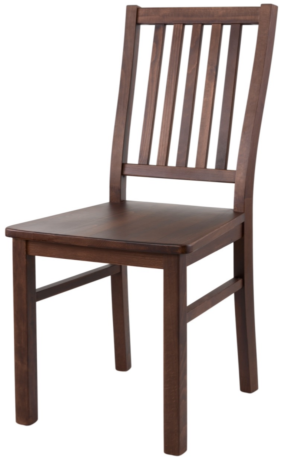
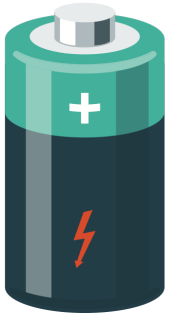
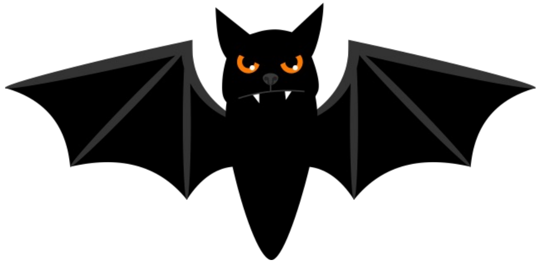
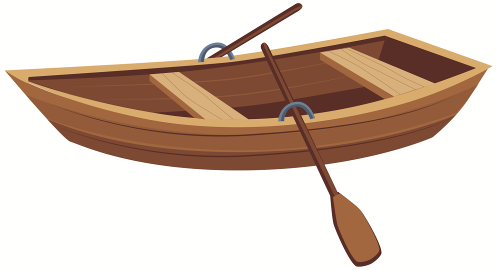
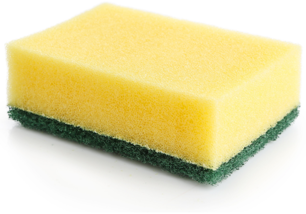

5. **Instruction:** Memorize the pictures in box A

BOX A

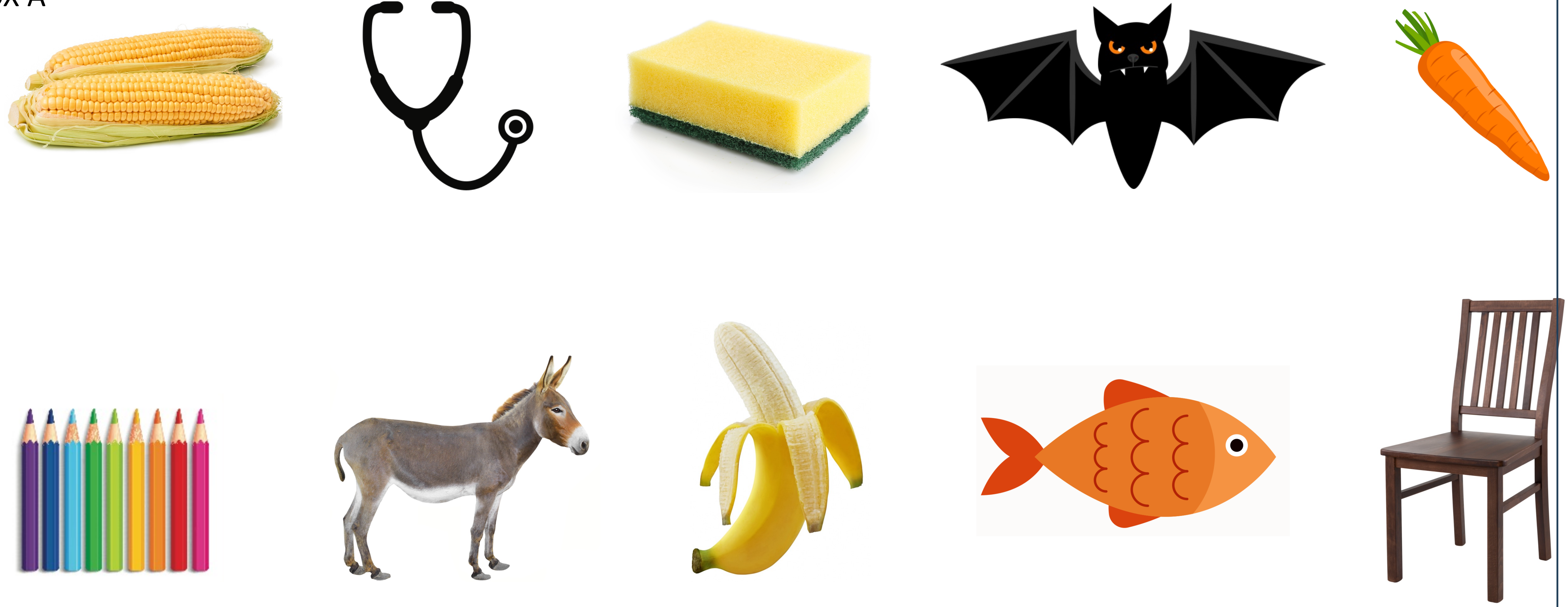

5. **Instruction:** Identify which pictures you recalled from box A and which are found in box B.

BOX B

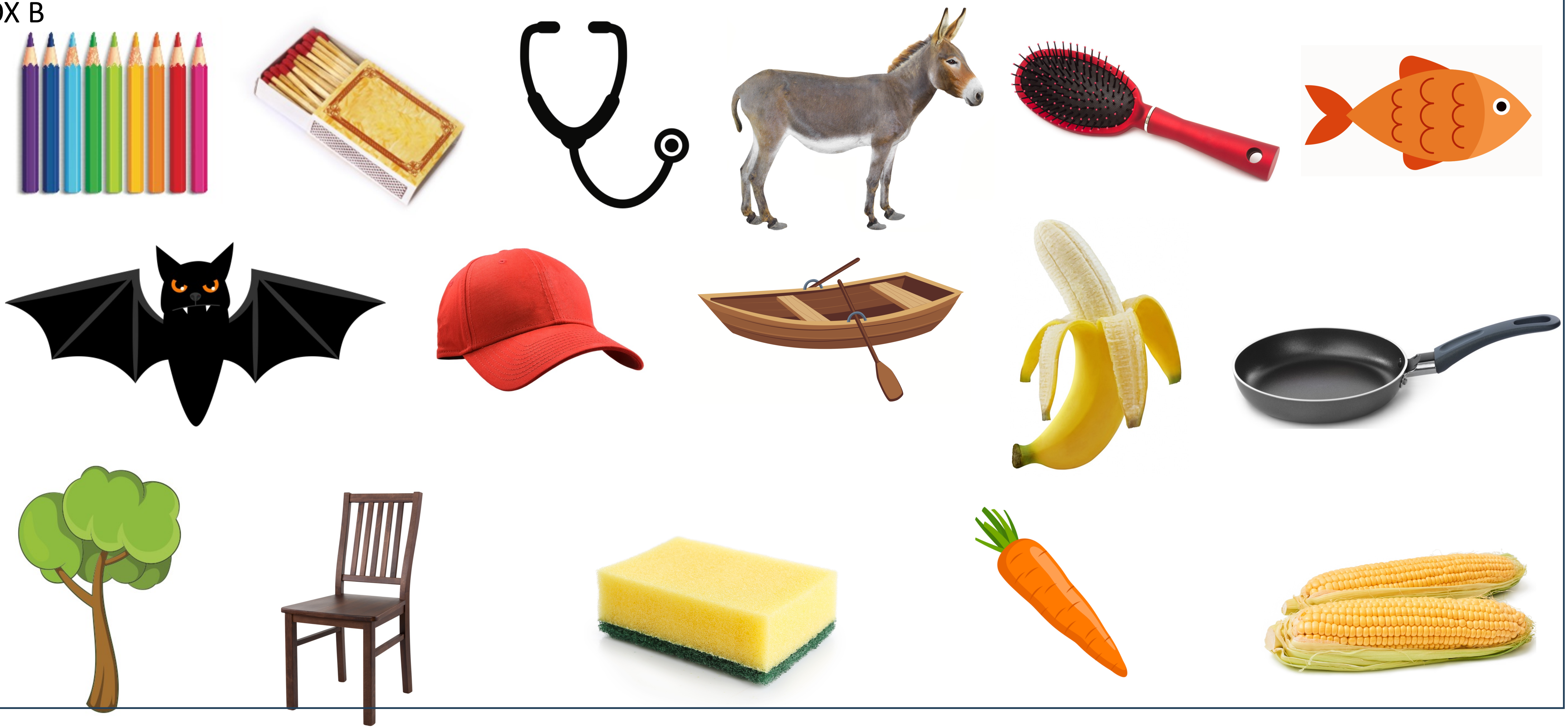

5. **Instruction:** Identify which pictures you recalled from box A and which are found in box B.

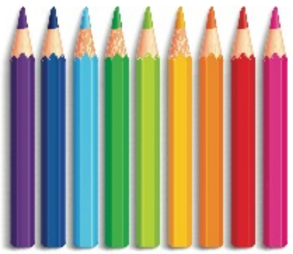
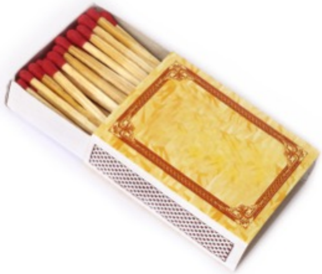
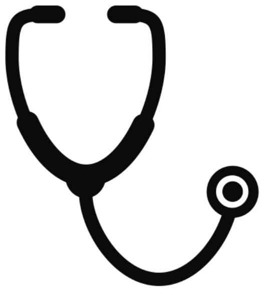
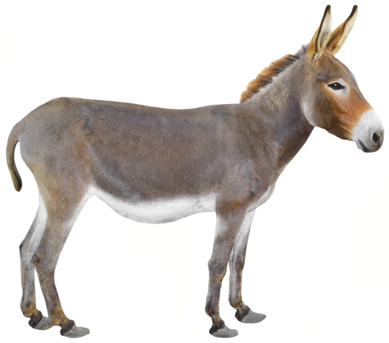
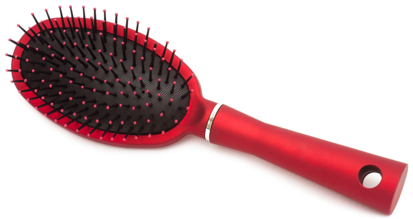
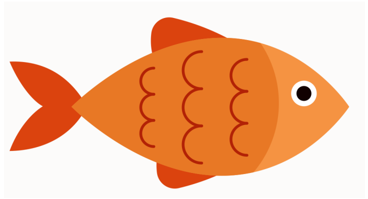
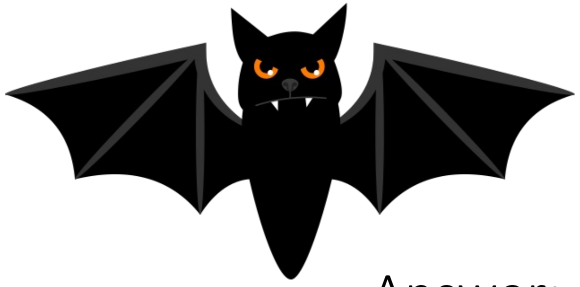
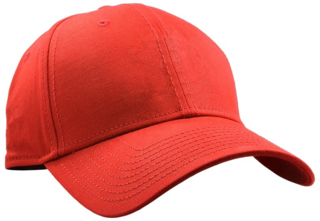
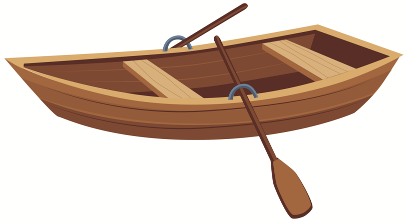
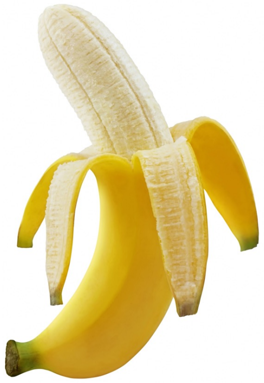
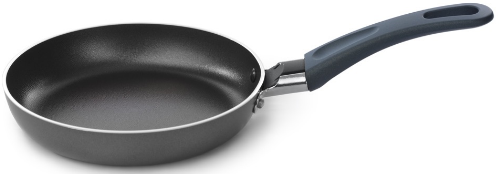

Answer: corn, stethoscope, sponge, bat, carrot, color pencil, donkey, banana, fish, chair

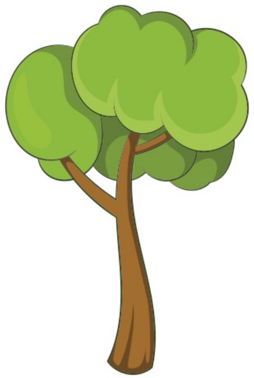
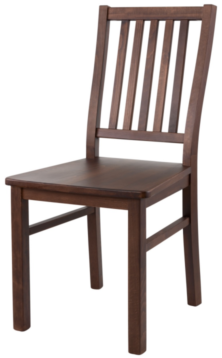
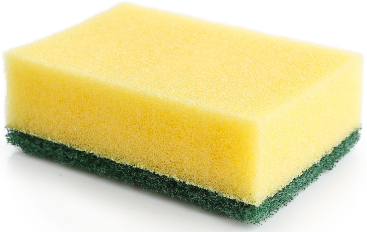
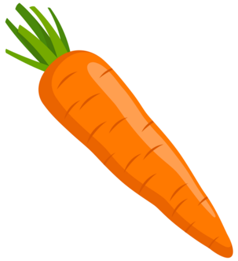
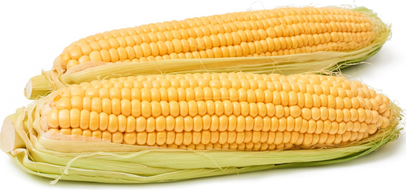

READING

Instructions: Read the following sentences

Instructions: Read the following sentences

1. She sells seashells by the sea shore
2. Fresh fried fish, fish fresh fried, fried fish fresh, fish fried fresh.
3. I scream, you scream, we all scream for ice cream!
4. I saw a kitten eating chicken in the kitchen.
5. If two witches were watching two watches, which witch would watch which watch.

Word Formation

Instructions: Discover the word:

1. NET – PLA
2. PU – TER – COM
3. VI – TE – SI - ON – LE

Word Formation

Instructions: Discover the word:

1. NET – PLA
2. PU – TER – COM
3. VI – TE – SI - ON – LE

Answer: planet, computer, television

4. **Instructions:** The word COLOR, just doesn't fit in:

a) in b)ful c)less

Answer: a

5. **Instructions:** Where could you buy a PAPER:

Answer: stationery shop

DRAWING

Instructions: Draw the following shapes on the answer sheet

Instructions: Draw the following shapes on the answer sheet

1. Circle
2. Triangle
3. Cube

Instructions: Copy the following forms on the answer sheet

4.

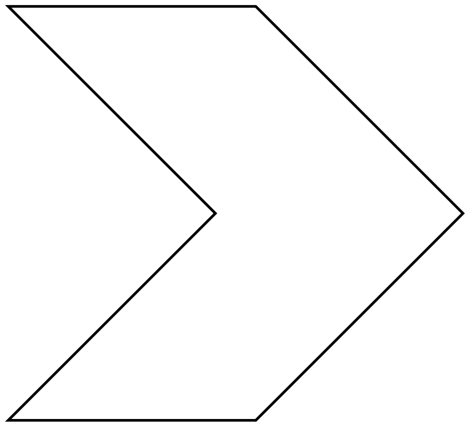

5.

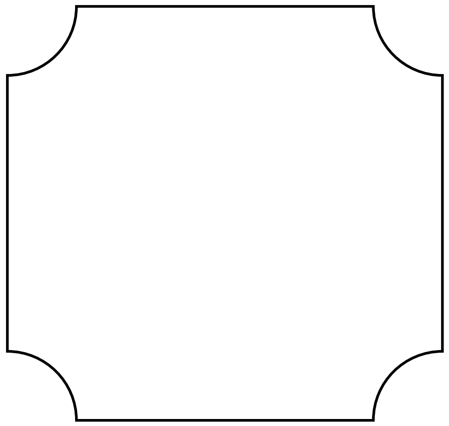

SPELLING

Instructions: Spell in the direct and reverse order

Spell in the direct order

1. ARC
2. WHALE
3. SANDWICH

Spell in reverse order

4. ADORE
5. CONTRACT
